# A germanium µ-oxo chloro dimer en route to a heavier phosgene analogue and 1,3-digermadioxetane dications

**DOI:** 10.1039/d6sc04935d

**Published:** 2026-09-15

**Authors:** Manoj Kumar, Julian Göbel, Prasenjit Palui, Viktoria H. Gessner

**Affiliations:** a Faculty of Chemistry and Biochemistry, Ruhr-University Bochum Universitätsstrasse 150 44801 Bochum Germany viktoria.gessner@rub.de

## Abstract

Heavier analogues of carbonyl compounds are challenging to isolate due to the intrinsic instability of E=O double bonds (E = Si–Pb). Herein, we demonstrate that a germanium(iv) µ-oxo chloro dimer, [YGe(Cl)O]_2_ (2), supported by an iminophosphinoyl-tethered yldiide ligand (Y = [Ph_2_P(NMes)-C-PPh_3_]^−^), serves as a versatile platform for the generation of unprecedented germanium oxo species. Treatment of the dimer with H_2_O·B(C_6_F_5_)_3_ enables the isolation of the first example of a Lewis acid–base stabilized germaphosgene, YH → Cl_2_GeO → B(C_6_F_5_)_3_ (3), through conversion of the yldiide into a carbodiphosphorane ligand upon protonation. Furthermore, halide abstraction from dimer [YGe(Cl)O]_2_ provides access to a series of unprecedented 1,3-digermadioxetane dications (4a–e), featuring a planar, square-shaped Ge_2_O_2_ core. Structural and computational analyses reveal highly polarized Ge–O single bonds in both systems, with no significant π-character. The strong donor ability of the ylide ligands is key to accessing compounds 3 and 4. While the newly formed carbodiphosphorane in 3 stabilizes the germaphosgene through its strong donor ability and the resulting Ge–O bond polarization, the yldiide in dimer 2 facilitates chloride abstraction and stabilizes the cationic charge in the dioxetane dications through charge delocalization into the entire ligand backbone.

## Abstract 2

The isolation, structural characterization, and bonding analysis of germanium analogues of phosgene and 1,3‑dioxetane dications stabilized by ylide ligands are reported.

## Graphical abstract

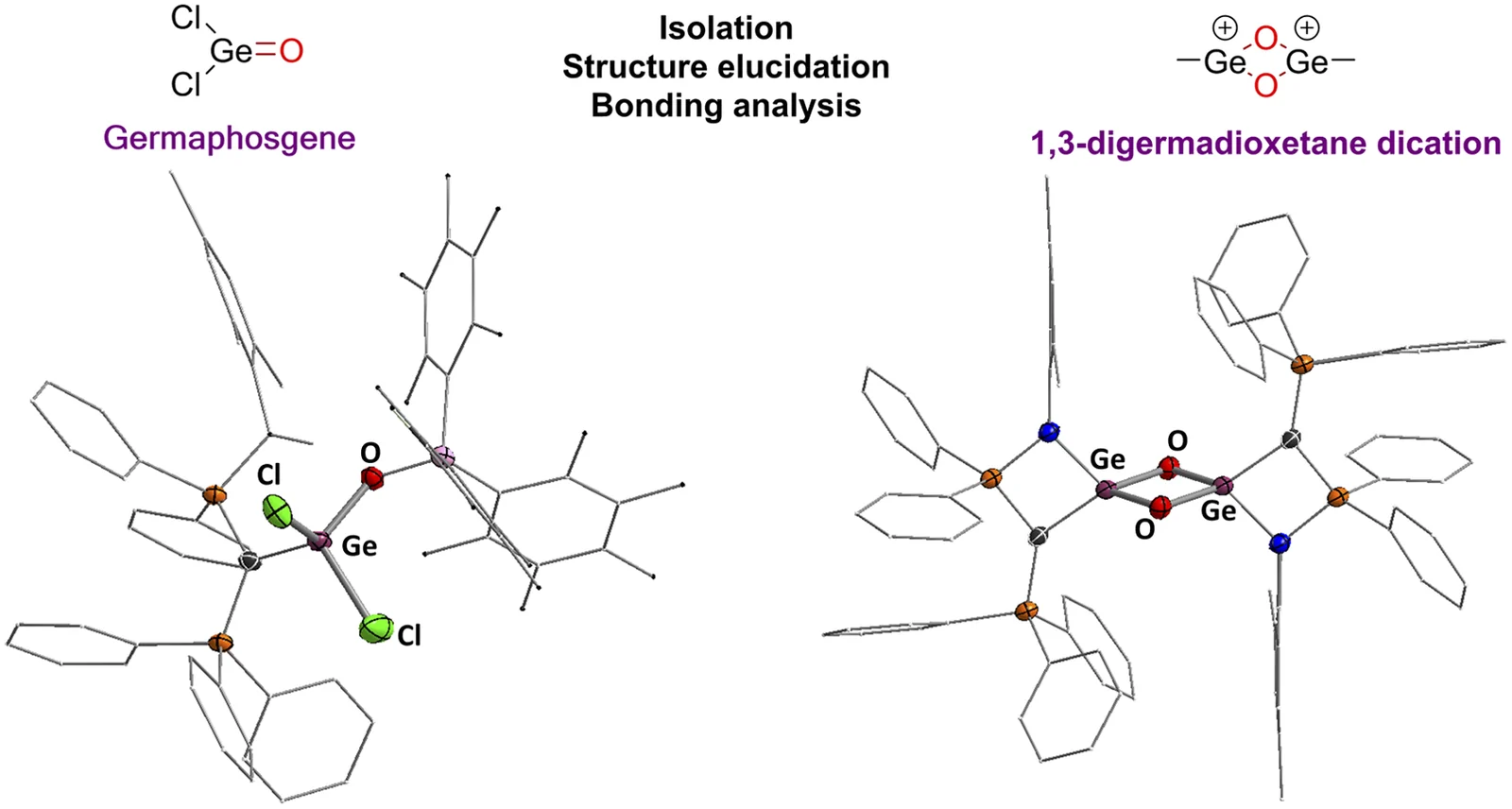

## Introduction

The carbonyl function (R_2_C=O) underpins the rich chemistry of important compound classes, including ketones, esters, amides, and carboxylic acids. The diverse reactivity of carbonyl compounds has driven extensive investigations into the chemistry of their heavier analogues, with the aim of reproducing and further elucidating their properties as carbonyl mimics.^1–10^ However, stable heavier carbonyl analogues remain rare due to the inherent weakness and high reactivity of the E=O bond (E = Si, Ge, Sn, Pb), arising from the significant electronegativity difference and poor p_π_–p_π_ overlap between the element and oxygen atom. This instability increases down the group of the periodic table and typically leads to rapid oligomerization or polymerization.^11–21^ While several silanones (Si=O) and germanones (Ge=O) have been reported, stannanones (Sn=O) are very rare, and a “true” plumbanone (Pb=O) is still elusive.^8–10^

To isolate heavier analogues, researchers have developed tailored ligands and donor–acceptor systems to electronically stabilize the E=O bond. The first preparative-scale synthesis of heavier ketones was reported by Driess and coworkers in 2007, employing a donor–acceptor strategy to tame the reactive Si=O moiety.^22^ Later, the same approach was applied to isolate germanones.^23^ Notable examples include Driess's and Iwamoto's Lewis base-stabilized, and Nagendran's Lewis acid–base stabilized germanones I, II, and III, supported by a β-diketiminato, a dialkyl, and an amino-troponiminate ligand, respectively (Fig. 1A).^24–26^ Recently, our group also succeeded in the isolation of a Lewis acid–base stabilized stannanone using a thiophosphinoyl-tethered ylide in combination with SnCl_2_ as Lewis acid.^27^ While multiple donor–acceptor stabilized silanones and germanones have been reported,^1–10^ the isolation of acid- and base-free heavier carbonyls has been more challenging and only achieved through additional kinetic and electronic stabilization. The use of extremely bulky substituents enabled the isolation of “genuine” silanones and germanones (IV, Fig. 1B), as well as the crystallographic identification of a stannanone, which, however, could not be isolated due to the extreme reactivity of the Sn=O bond.^28–32^ Additionally, silanones and germanones have also been accessed through electronic stabilization using strong internal σ-/π-donor groups. For example, in 2021, Inoue reported the three coordinated germanone V (Fig. 1B) stabilized by bulky N-heterocyclic imines (NHI),^33^ and more recently, Aldridge and coworkers applied the same approach to synthesize a stable boryl-substituted amidinato-silanone and its germanium analogue.^34^

**Fig. 1:**
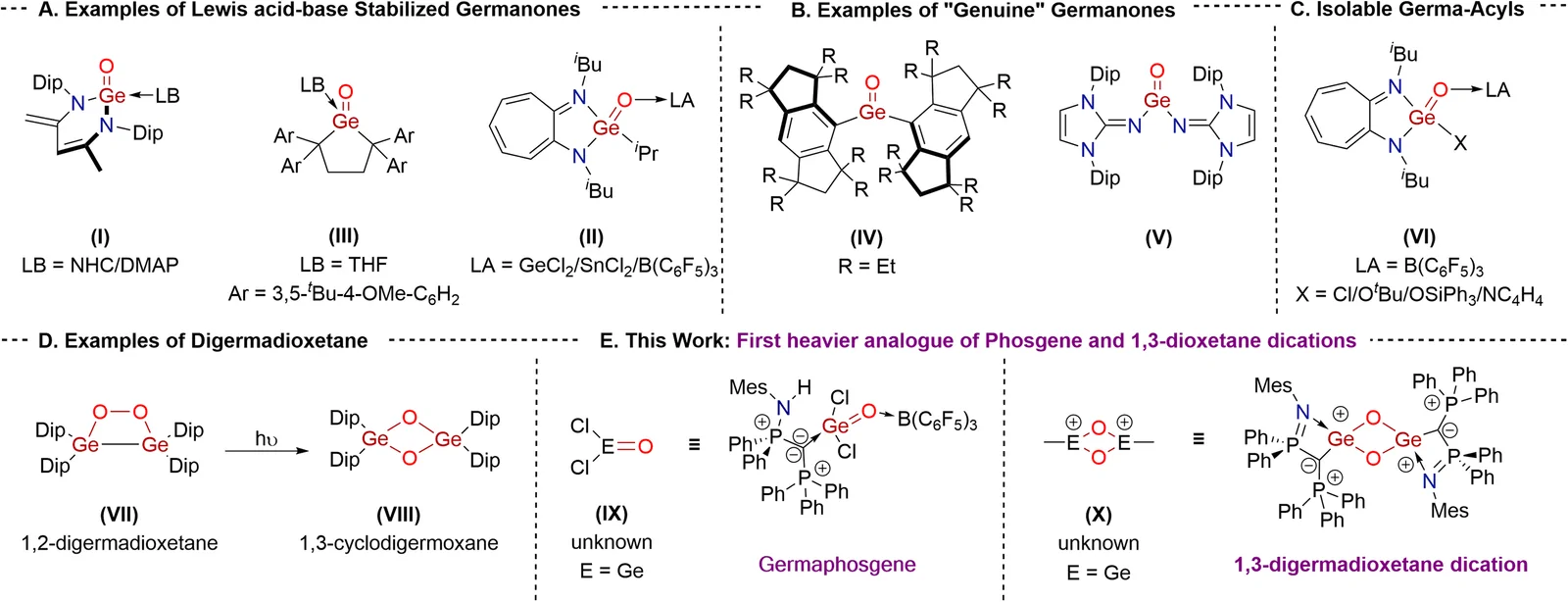
(A) Examples of isolated acid/base and germanones (LB = Lewis base, LA = Lewis acid, Dip = 2,6-diisopropylphenyl, DMAP = 4-dimethylaminopyridine), (B) reported “true” germanones, (C) synthesized and characterized Lewis-acid-stabilized germaacyl compounds, (D) reported heavier 1,2- and 1,3-digermadioxetanes, (E) donor–acceptor stabilized heavier analogue of phosgene and 1,3-digermadioxetane dication reported in this work.

These breakthroughs in heavier ketone synthesis also paved the way for the isolation of Si and Ge analogues of aldehydes, esters, amides, and other carbonyl compounds.^35–42^ For instance, Aldridge and coworkers reported the first Lewis-acid-free sila-acyl chloride, which served as a precursor to sila-aldehyde and sila-ester derivatives.^41^ In 2011, Ghadwal *et al.* demonstrated the utility of donor–acceptor stabilization for isolating the first example of a sila-acid anhydride, followed by a coordinatively trapped sila-formyl chloride in 2012.^38,39^ Germanium analogues of carboxylic acid derivatives have also been described. For example, in 2019, Nagendran's group systematically varied the X substituent (X = Cl, O^*t*^Bu, OSiPh_3_, NC_4_H_4_) on a germylene precursor, affording a series of isolable, Lewis acid–base stabilized germacarbonyls (VI, Fig. 1C) after oxidation.^43^

Phosgene (Cl_2_C=O) represents a particularly interesting member of the family of carbonyl compounds, serving as a highly reactive and versatile C1 building block with broad industrial utility,^44,45^ as reflected in its multimillion-ton annual production *via* the catalyzed reaction between carbon monoxide and chlorine gas.^46–49^ Its extreme reactivity stems from its highly electrophilic carbonyl carbon atom paired with chlorides as leaving groups, rendering it indispensable for numerous key transformations, particularly in polymer and pharmaceutical synthesis. In contrast, the synthesis and isolation of a heavier phosgene analogue (Cl_2_E=O, where E = Si, Ge, Sn, and Pb) remain a formidable challenge (IX, Fig. 1E).

Our group has recently demonstrated the potential of ylide groups to stabilize reactive main-group compounds and to promote the activation of small molecules.^50–53^ Building on these findings, we hypothesized that a tailored ylide ligand could enable the stabilization of germanium analogues of different carbonyl compounds. Herein, we report the successful isolation of the first Lewis acid–base-stabilized germaphosgene CDP → Cl_2_Ge=O → B(C_6_F_5_)_3_ (CDP = carbodiphosphorane). Its synthesis is accomplished from a Ge(iv) µ-oxo chloro dimer through conversion of an iminophosphinoyl-substituted yldiide into a CDP ligand upon reaction with H_2_O·B(C_6_F_5_)_3_. In addition, we demonstrate that this µ-oxo chloro dimer serves as a precursor to a heavier 1,3-digermadioxetane dication (X, Fig. 1E) through halide abstraction. In contrast to neutral heavier 1,3-digermadioxetanes (*e.g.*VIII, Fig. 1D) – which are accessible from the isomeric 1,2-digermadioxetane^54–57^ – the corresponding dications have remained unexplored to date, and only been investigated *in silico*.^58,59^

## Results and discussion

Recently, our group reported the use of a mesityl-substituted iminophosphinoyl yldiide in the isolation of a unique aromatic Ge_4_ cycle through the reduction of chlorogermylene 1 (Fig. 2).^60,61^ Given the readily availability of 1, we set out to explore its utility for accessing compounds featuring a formal Ge=O double bond through oxidation. Exposure of a solution of compound 1 in THF to 1 bar of nitrous oxide resulted in the discoloration of the bright yellow solution within one hour at room temperature and led to the formation of germanium µ-oxo chloro dimer 2, which could be isolated as a colorless solid in 82% yield. The facile isolation of 2 is noteworthy because earlier oxidation attempts on the analogous tolyl-substituted congener of 1, [Y*_N_*_-Tol_-GeCl], did not afford any stable Ge-oxo product. This suggests that the greater steric bulk of the *N*-Mes substituent, compared with the *N*-Tol group, is essential for stabilizing the oxo species.

**Fig. 2:**
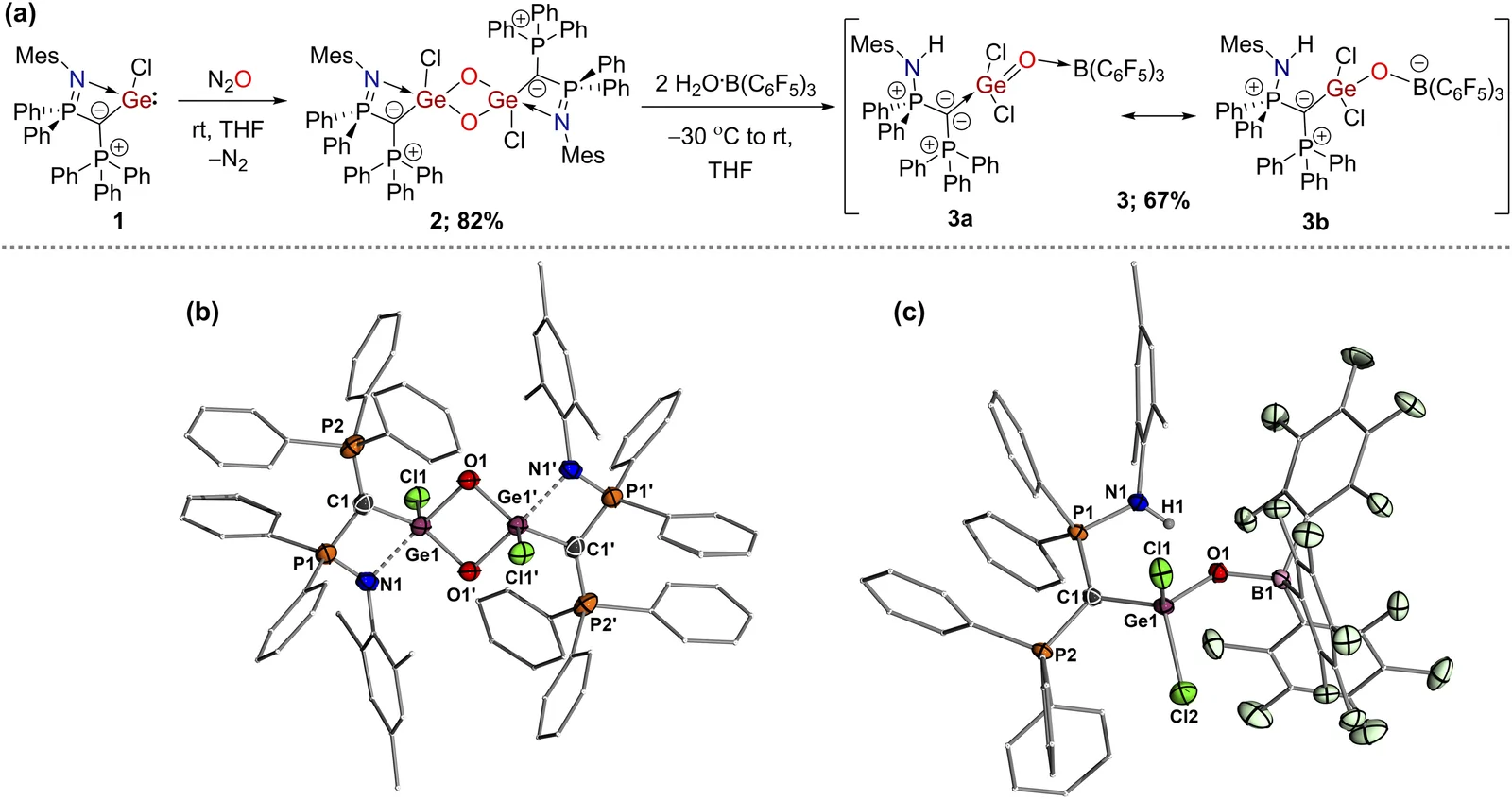
(a) Synthesis of germanium µ-oxo chloro dimer 2 through oxidation of chlorogermylene 1 and isolation of the germanium analogue of phosgene 3a. (b) Molecular structure of 2 and (c) molecular structure of 3a (below). Thermal ellipsoids are drawn at 50% probability level. Hydrogen atoms are omitted for clarity. Important bond lengths [Å] and angles [°] are given in Table 1 and in the SI Fig. S39 and S40.

The germanium µ-oxo chloro dimer 2 is highly soluble in dichloromethane, acetonitrile, and chloroform but poorly soluble in toluene and ethereal solvents. Compound 2 was characterized by multinuclear NMR spectroscopy and single-crystal X-ray diffraction (XRD) analysis (see below and SI for details). In the ^31^P{^1^H} NMR spectrum, the iminophosphinoyl group appears as a broad signal between 38.3 and 35.6 ppm, while the phosphonium group gives rise to a sharp doublet at 11.4 ppm. Single crystals of compound 2 were obtained by diffusing hexane into a CH_2_Cl_2_ solution of 2 at room temperature. The dimer (Fig. 2 and Table 1) features a central, planar Ge_2_O_2_ ring with noticeably unequal Ge–O bond lengths of 1.788(2) and 1.857(2) Å. These values fall within the range reported for other µ-oxo dimers, such as Nagendran's amino-troponiminate system (1.818(3), 1.823(3) Å) and Xie's carboranyl-substituted µ-oxo chloro dimer (1.836(2), 1.764(3) Å).^20,26^ The Ge–N distance of 2.073(2) Å lies between the corresponding distances in these two systems (1.984(4)/1.998(4) and 2.125(4) Å, respectively), indicating a strong dative interaction. Accordingly, the Ge–Cl bond (2.2403(6) Å) in 2 is significantly longer than that observed in Xie's carboranyl µ-oxo chloro dimer (2.132(1) Å), indicating a weakening of this bond and enhanced reactivity towards bond cleavage.

**Table 1:** Selected bond lengths and angles for compounds 2, 3, and 4a

	2	3	4a
P1–C1 [Å]	1.714(2)	1.737(2)	1.735(3)
P2–C1 [Å]	1.694(2)	1.746(2)	1.707(3)
C1–Ge1 [Å]	1.939(2)	1.911(2)	1.874(3)
Ge1–O1 [Å]	1.788(2)	1.728(1)	1.795(2)
Ge1–O1′ [Å]	1.857(2)	—	1.799(2)
Ge1–N1 [Å]	2.073(2)	—	1.860(2)
O1–B1 [Å]	—	1.502(2)	—
P1–C1–P2 [°]	133.4(1)	120.9(1)	131.2(2)
O1–Ge1–O1′ [°]	95.2(7)	—	92.3(9)
O1–Ge1–O1′ [°]	84.7(7)	—	87.7(9)

Given the distinctive structural features of dimer 2, we turned our attention toward investigating its reactivity, focusing on cleavage of a Ge–O bond and removal of the chloride ligand. Our initial experiments aimed to cleave the dimer and generate the corresponding Lewis-acid-stabilized germaacyl chloride. To this end, compound 2 was treated with two equiv. of B(C_6_F_5_)_3_ in dry toluene at −30 °C. Monitoring the reaction by ^31^P{^1^H} NMR spectroscopy showed the consumption of 2 and the emergence of two new doublets at 38.0 (Ph_2_PN) and 21.3 (Ph_3_P) ppm, slightly downfield from the starting material, along with the phosphonium salt of the ligand as the major product (*ca.* 89%), characterized by doublets at 32.1 and 19.3 ppm. Surprisingly, crystallization of the crude reaction mixture afforded single crystals of a minor product, which was identified by XRD analysis as compound 3, representing a Lewis acid–base-stabilized germanium analogue of phosgene, rather than the targeted Lewis acid-stabilized germaacyl chloride.

The formation of 3 can be attributed to trace amounts of water present in B(C_6_F_5_)_3_, which results in the protonation of the iminophosphinoyl moiety of the ylide ligand, thus converting the anionic yldiide into a neutral carbodiphosphorane ligand. Repeating the reaction with two equivalents of separately prepared H_2_O·B(C_6_F_5_)_3_ in dry THF at −30 °C enabled the quantitative formation of 3 and its isolation in 67% yield. Protonation at the imine nitrogen was unambiguously confirmed by ^1^H NMR spectroscopy, showing a doublet at 7.76 ppm for the NH proton with a ^2^*J*_PH_ coupling constant of 10.1 Hz. The ^11^B{^1^H} NMR spectrum of compound 3 displays a single resonance at *δ* = −3.4 ppm, shifted slightly upfield relative to that of the free Lewis acid B(C_6_F_5_)_3_ (*δ* = −2.3 ppm).^62^^19^F NMR spectroscopy revealed marked differences in the chemical shifts between compound 3 (*δ* = −133.0, −162.7, and −166.8 ppm) and B(C_6_F_5_)_3_ (*δ* = −128, −143, and −160 ppm).^63^

While the synthesis of germaphosgene proved reproducible, its formation mechanism could not be unambiguously determined. Reaction monitoring of the formation of compound 3 revealed, in addition to 3, the formation of the corresponding phosphonium salt of the ligand [*δ*_P_ = 32.0 and 19.3 ppm] (see SI). This phosphonium salt presumably arises from the formation of HCl, as previously observed in reactions of chlorosilylenes with H_2_O·B(C_6_F_5_)_3_.^38,39^ The formed phosphonium salt suggests that the reaction of 2 with H_2_O·B(C_6_F_5_)_3_ proceeds *via* ligand protonation and HCl generation, which ultimately results in the protonative cleavage of one ligand from the dimer, presumably releasing an NMR-silent germanium oxo species.

In the molecular structure of 3, the germanium(iv) center adopts a distorted tetrahedral geometry, coordinated by the two chlorine atoms, the ylidic carbon and the oxygen atom. The Ge1–O1 bond length of 1.728(1) Å is shorter than the Ge–O single bonds in dimer 2 (1.788(2) and 1.857(2) Å, Table 1), but longer than those reported for tricoordinate germanones and most Lewis acid/base stabilized germanones [1.646(2)–1.720(2) Å] (I–V, Fig. 1A).^24–26,28,33^ Notably, the Ge1–O1 (1.728(1) *vs.* 1.698(2) Å), and the O1–B1 (1.502(2) Å *vs.* 1.493(5) Å) bond distances in 3 are comparable to those reported by Nagendran for Lewis acid-stabilized germaacyl chloride VI. The slight elongation in 3 presumably can be explained by the strong electron donation from the carbodiphosphorane ligand.^43^ Accordingly, the Ge1–C1 bond in 3 of 1.911(2) Å is shortened compared to 1.939(2) Å in compound 2, while the P1–C1–P2 angle decreased from 133.4(1)° to 120.9(1)°, in line with the decreased ^2^*J*_PP_ coupling constant in 3. These observations can be attributed to changes in the bonding of the ligand upon protonation. Whereas the germanium center in oxo dimer 2 accepts electron density from both the ylidic carbon and the imine nitrogen, protonation of the imine in 3 disrupts this N → Ge interaction, rendering the germanium center more electrophilic. Consequently, the ylidic carbon center of the CDP donates more strongly to germanium, resulting in the shortened Ge–C bond. Notably, this bond length is also shorter than those in the CDP–GeCl_2_ and CDP–GeCl_2_–GeCl_2_ complexes (2.063 Å each) reported by Alcarazo and coworkers, which is in line with the higher oxidation state of Ge in 3.^64^

To gain further insights into the bonding situation, density functional theory (DFT) calculations on the PBE0/def2-TZVPP/D3BJ/CPCM(THF)//ωB97M-V/def2-TZVPP/SMD(THF) level of theory were performed for 3 and its Lewis acid-free analogue 3′ (see SI for details). The optimized structure of 3 reproduces the experimentally observed structural parameters, including the elongated Ge–O bond relative to typical tricoordinated germanones (IV and V), and the Lewis-acid-stabilized germaacyl chloride VI (with X = Cl). In the Lewis acid-free analogue 3′, the Ge–O bond shortens from 1.736 in calculated 3a to 1.688 Å, consistent with an increased bond order (Table S5). However, analysis of the electronic structure of 3 and 3′ showed no π-symmetric molecular orbital indicative of a classical Ge=O double bond. Instead, only σ-type bonding orbitals are found for all bonds around germanium, with the σ-orbital associated with the Ge–O linkage being strongly polarized toward the oxygen center. Natural bond orbital (NBO) analysis of compound 3 reveals single bonds between germanium and the adjacent atoms, with the exception of the Ge–O interaction, which is only described as a strong donor–acceptor interaction from oxygen to germanium (*E*(2) = 190.75 kcal mol^−1^) in the second-order perturbation theory analysis, consistent with a dative O → Ge bond. In contrast, the O–B interaction is best described as a polarized covalent bond with a significant ionic contribution (81.1% at oxygen). This ionic bonding picture obtained from NBO analysis for the E=O linkage is in line with previous reports on heavier carbonyl compounds.^27^ Intrinsic bond orbital (IBO) analysis of 3 further supports the description of polarized σ-bonding orbitals for all bonds, with no π-bonding orbital for the Ge–O interaction. In contrast, the Mayer bond index (MBIs) of 1.11 for the Ge–O bond is higher than the values typically calculated for Ge–O single bonds. This value further increases for 3′ (MBI = 1.48), consistent with the shorter bond length and an increased double bond character in the Lewis acid-free germaphosgene (Fig. 3).

**Fig. 3:**
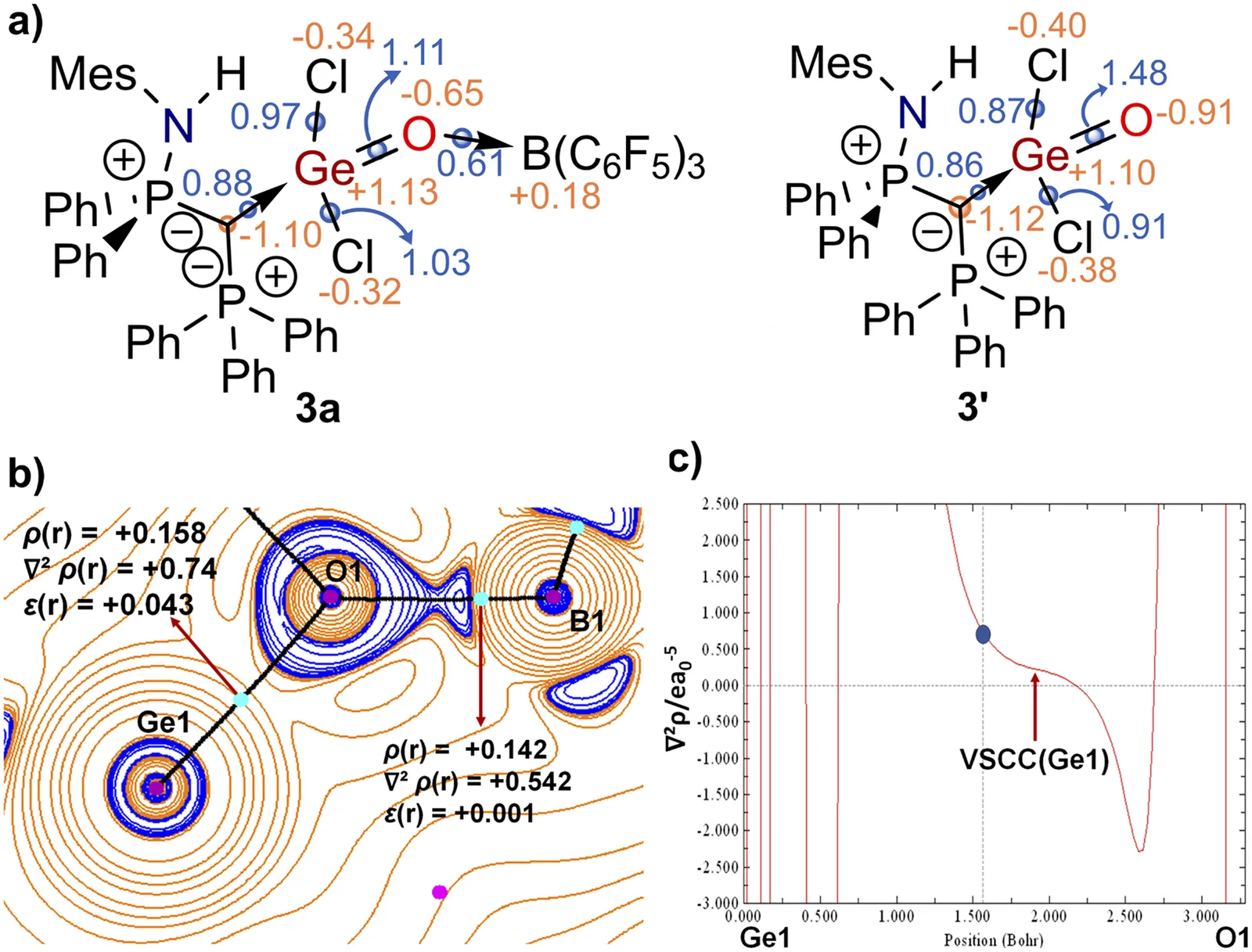
(a) Display of the IPA charges (orange) and Mayer bond indices (blue) for 3a and 3′; (b) contour plot of the Laplacian of electron density in 3a. BCPs of the Ge1–O1–B1 linkage are shown as cyan dots with the respective values for the charge density *ρ* and ellipticity *ε* of the electron density. The charge density is given in a.u. (c) 1D Laplacian profiles of the Ge1–O1 bond-path in 3a.

Quantum theory of atoms in molecules (QTAIM) analysis revealed bond critical points (BCPs) for all bonds to germanium. The electron density at the BCP of the Ge–O linkage exhibits low ellipticity values (*ε* = 0.043 for 3a and *ε* = 0.014 for 3′), further confirming the σ-type bonding with negligible π-character (Fig. 3c). Analysis of the Laplacian profile along the bond path shows a pronounced valence-shell charge concentration (VSCC) near oxygen and only a weak feature near germanium. Both VSCCs reside within the oxygen basin, supporting a dative O → Ge interaction, in agreement with the NBO analysis (Fig. 3d). Therefore, the germaphosgene 3a can also be described by resonance structure 3b.

In the solid state, compound 3 is stable for several weeks under inert conditions but decomposes upon prolonged standing in DCM solution (Fig. S8) and immediately in acetonitrile, DMSO, and chloroform. Given the presence of two chloride groups at germanium, we examined a possible chloride abstraction to access the corresponding acylium ion. However, no reaction was observed upon treatment with 2 equiv. of NaBAr^F^_4_, whereas with AgOTf the reaction proceeded in an unselective manner, precluding isolation of a Lewis-acid-stabilized germa-acylium ion. Furthermore, the reaction of 3 with amines or alcohols resulted in its decomposition, yielding the corresponding protonated ylide.

Having established the possible cleavage of dimer 2, we also examined its use as a precursor for the synthesis of a Ge(iv) µ-oxo dication *via* chloride abstraction. To this end, we carried out metathesis reactions with salts of different weakly coordinating anions (Fig. 4a). To our delight, reaction with 2 equiv. of a silver or sodium salt (*e.g.*, NaBAr^F^_4_, AgSbF_6_, and AgOTf) in DCM afforded the targeted dications 4a–c in high yields. Likewise, treatment of 2 with two equivalents of the strong Lewis acid B(C_6_F_5_)_3_ in DCM at room temperature yielded dication 4d with chloroborate B(C_6_F_5_)_3_Cl as a counter anion. This contrasts with the reactivity of Nagendran's amino-troponiminate-stabilized germanium(iv) µ-oxo chloro dimer, which yielded the donor–acceptor-stabilized germaacyl chloride VI upon treatment with 2 equiv. of B(C_6_F_5_)_3_.^43^ This difference can presumably be attributed to the stronger donor ability of the ylide ligand in our system, which enhances bond polarization, weakens the Ge–Cl bond, and thus facilitates chloride abstraction. Likewise, treatment of the triflate salt 4c with two equiv. of B(C_6_F_5_)_3_ led to the anion exchange, yielding borate 4e, which can also be synthesized *via* Cl/OTf exchange from 4d using AgOTf.

**Fig. 4:**
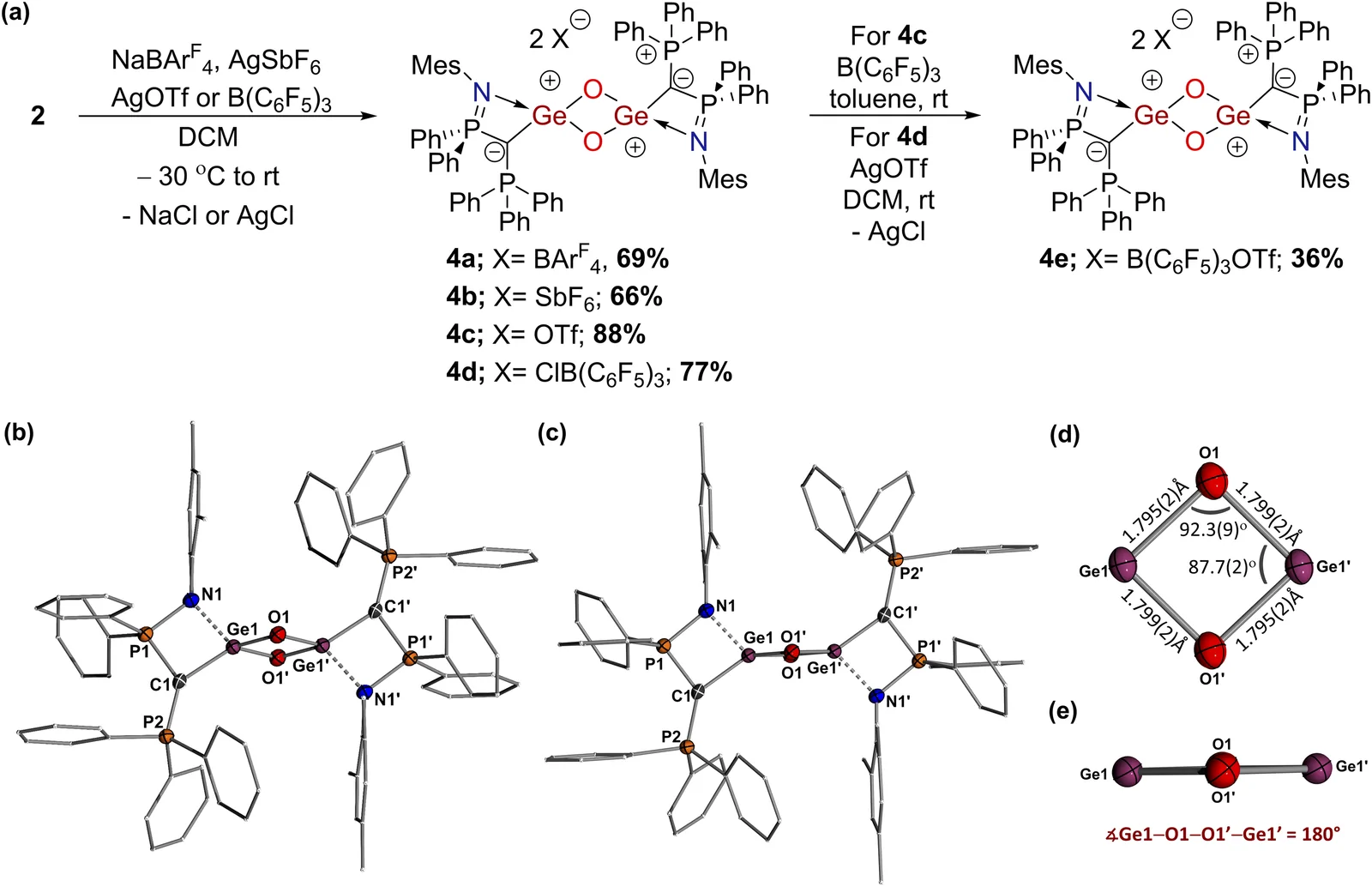
(a) Synthesis of 1,3-digermadioxetane dications 4a–e, (b) solid-state structure of 4a with thermal ellipsoids drawn at 50% probability level. Hydrogen and the counter anion (BAr^F^_4_) are omitted for clarity. (c) Side view of 4a showing the planarity of the Ge_2_O_2_ core and the perpendicular arrangement of the ylide ligands relative to the Ge_2_O_2_ ring. (d and e) Top and side view of 4a with ligand omitted for clarity.

Compounds 4 represent the first examples of 1,3-digermadioxetane dications. ^31^P{^1^H} NMR spectroscopy of the salts 4a, 4b, and 4d revealed two sets of doublets in an approx. 60 : 40 ratio. For 4a, the two doublets corresponding to the iminophosphinoyl group appear at 46.1 and 45.6 ppm, while the two doublets for the Ph_3_P group appear at 13.7 and 13.4 ppm with ^2^*J*_PP_ coupling constants of 27.0 and 25.5 Hz, respectively (see SI). Similar shifts and coupling constants are observed for the salts 4b and 4d. These two sets of signals can be attributed to the formation of mixtures of *syn*- and *anti*-isomers in solution (Scheme 1). Both isomers were observed directly in the reaction mixture as well as in a DCM solution obtained from isolated crystals of 4, which were found to contain exclusively the *anti*-isomers (see below). Attempts to enrich either isomer by fractional crystallization were unsuccessful, suggesting that the two forms are in equilibrium with each other in solution. Indeed, DFT calculations revealed a small energy difference of only 1.2 kcal mol^−1^ in favour of the *anti*-isomer, supporting that both isomers are energetically accessible.

**Scheme 1:**
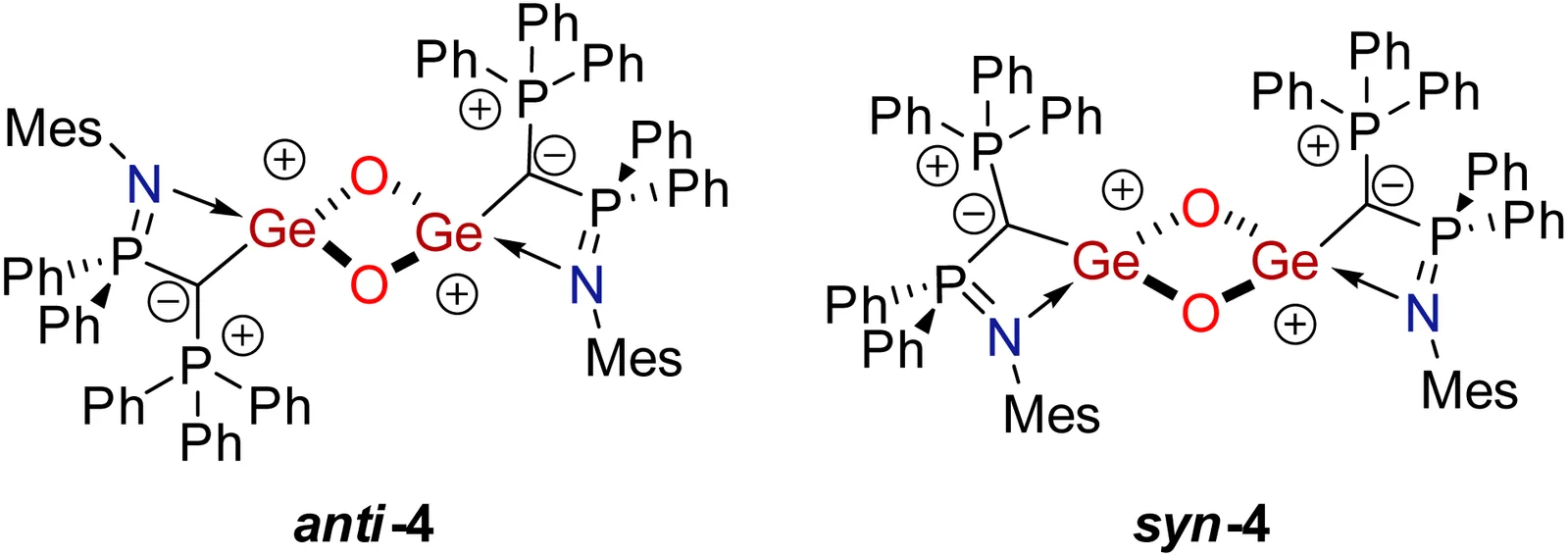
Proposed isomers *anti* and *syn* for 1,3-digermadioxetane dications 4.

In contrast to compounds 4a, 4b, and 4d, triflate salt 4c exhibits only a single set of doublets at 45.9 ppm (Ph_2_P=N) and 13.5 ppm (Ph_3_P) in the ^31^P{^1^H} NMR spectrum. The same observation was made for compound 4e, indicating that 4c and 4e exist as a single isomer in the solution state. However, the ^2^*J*_PP_ coupling constant of approx. 26.4 Hz in both salts lies between the values found for the *syn*- and *anti*-isomers of 4a, 4b, and 4d, suggesting that the single set of signals for 4c and 4e arises from slow interconversion (relative to 4a, 4b, and 4d) between the two isomers on the NMR timescale at room temperature. Indeed, variable-temperature (VT) ^31^P{^1^H} NMR experiments of compound 4c showed a broadening of the resonances at 5 °C, followed by splitting into two sets of doublets at −15 °C and below (Fig. S22). This demonstrates that the dioxetanes exist in solution as an equilibrium mixture of *syn-* and *anti*-isomers, with the barrier for their interconversion depending on the counter-anion. Notably, the triflate and triflate-based anions in 4c and 4e appear to increase the activation barrier, likely due to stronger anion–cation interactions, thereby impeding interconversion between the *syn*- and *anti*-isomers.

The digermadioxetane dications 4a–e could be characterized by single-crystal XRD analysis. In all crystal structures, only the *anti* isomer was present, independent of the counter-anion (see SI). All structures exhibit inversion symmetry, with only half of the molecule in the asymmetric unit (Fig. 4b and Table 1). As all structures exhibit nearly identical bonding parameters, only those of the BAr^F^_4_ salt 4a are discussed in the following. The central structural motif of 4a consists of a perfectly planar, four-membered Ge_2_O_2_ ring (∡Ge1–O1–O1′–Ge1′ = 180°), to which the ylide ligands coordinate in an almost perpendicular manner. The Ge–O distances of 1.795(2) Å and 1.799(2) Å are nearly equivalent, indicating a more uniform bonding situation compared to the neutral precursor 2, whose Ge_2_O_2_ core is markedly unsymmetrical (Ge–O: 1.857(2) Å and 1.788(2) Å). This asymmetry is alleviated upon chloride abstraction, also resulting in the ring angles (Ge–O–Ge–O) approaching an ideal square structure. As such, the Ge–O–Ge angle decreases from 95.2(7)° to 92.3(9)°, while the O–Ge–O angle widens from 84.7(7)° to 87.7(9)°. As a consequence of the cationic charge, the Ge1–C1 bonds contract from 1.939(2) Å in 2 to 1.874(3) Å in 4a, reflecting the increased electron donation from the ylide ligand to the more electron-deficient germanium center. This shift in electron density also results in an elongation of the adjacent ylidic P2–C1 bond from 1.714(2) Å in compound 2 to 1.735(3) Å in 4a due to reduced electrostatic attraction. In contrast, the P1–C1 bond slightly shortens, presumably due to the strengthened Ge–N interaction.

To gain further insights into the bonding situation, DFT calculations on the 1,3-digermadioxetane dication 4 in comparison to its chloride precursor 2 were performed. The calculations confirm that the transition from 2 to 4 is accompanied by a pronounced redistribution of electron density, as evidenced by the intrinsic atomic orbital (IAO) charges and Mayer bond indices (Fig. 5a). The IAO partial charge on germanium increases slightly from +1.23 in 2 to +1.34 in 4, reflecting the increased electrophilicity of Ge upon chloride abstraction. However, the majority of the positive charge is delocalized into the ylide ligand, with the most pronounced change involving the partial charge at the C1 carbon and the MBI of the N–Ge bond. The charge at C1 increases from −1.15 in 2 to −0.81 in 4. This shift of electron density is accompanied by an increase in the Ge–C bond strength, as reflected in the increased MBI (0.99 *vs.* 0.90) and the shortened bond found in experiment. Likewise, the N → Ge donation strengthens substantially to alleviate the positive charge at Ge, which is evidenced by the increased N–Ge bond index (from 0.6 in 2 to 1.1 in 4) as well as the markedly increased second-order perturbation energy from 92 kcal mol^−1^ in 2 (Table S9) to 110 kcal mol^−1^ in 4 (Table S15). The charges at the nitrogen and phosphorus centers change only slightly (<0.05*e*). Given the increase of positive charge at germanium, compound 4 can be described as a digermadioxetane, with its stability arising from contributions of other canonical structures, including that of a phosphonium cation.

**Fig. 5:**
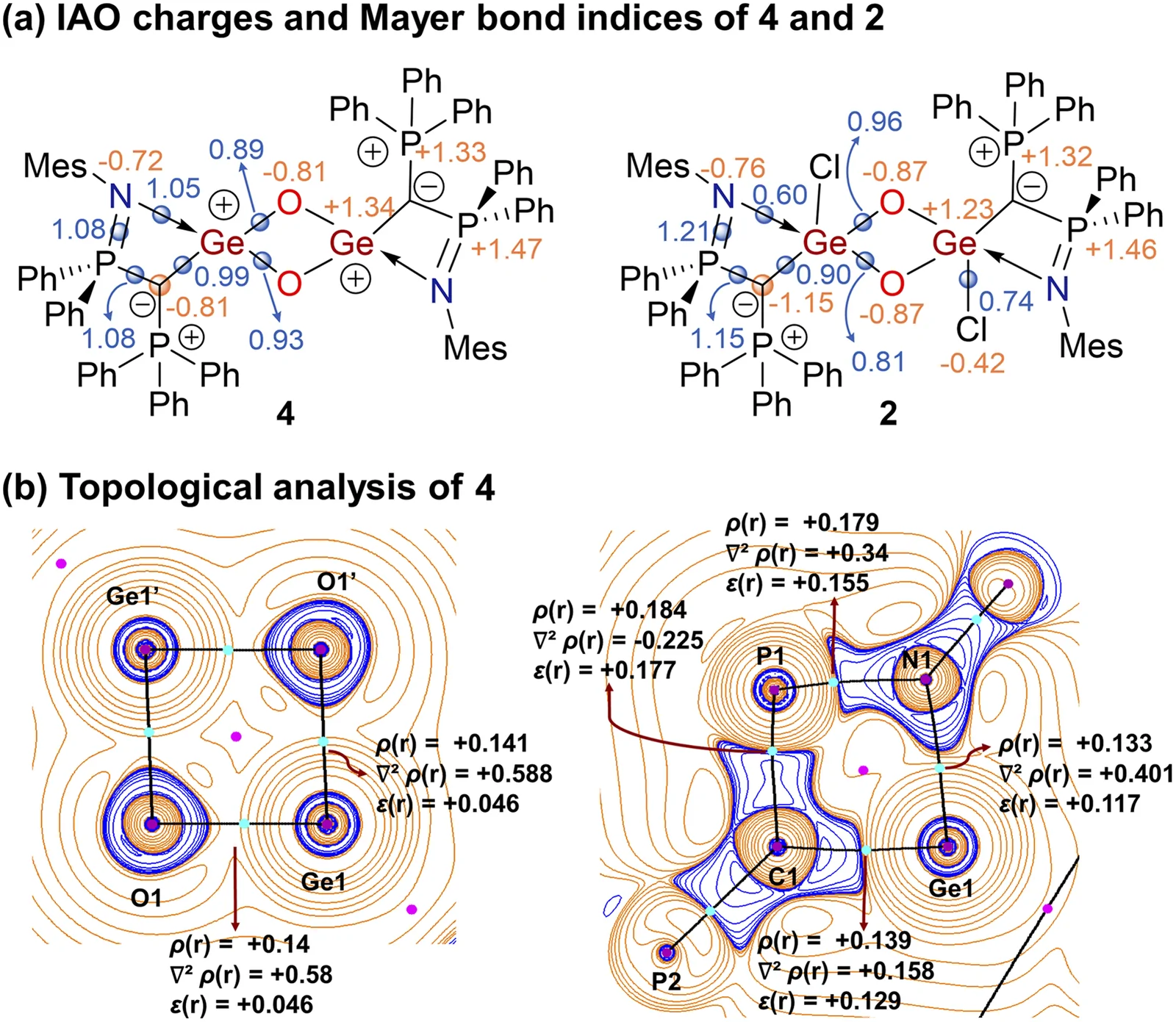
(a) Display of the IAO charges (orange) and Mayer bond indices (blue) of compounds 4 and 2, respectively. (b) Display of the Laplacian of electron density of 4. BCPs of the Ge1–O1–Ge′–O1′ ring and C1–P1–N1–Ge1 linkage of 4 are shown as cyan dots with the respective values for the charge density *ρ* and ellipticity *ε* of the electron density. For 4, average values for the *syn*- and *anti*-isomer are given. The charge density is given in a.u.

The charge redistribution within the Ge_2_O_2_ ring of the dication also results in increased and nearly equal Ge–O bond indices and a slightly more positive charge at the oxygen centers. Nonetheless, Natural Bond Orbital (NBO) analysis yields an ionic bonding situation in the Ge_2_O_2_ ring for both 2 and 4a, whereas IBO analysis reveals σ-type Ge–O bonding orbitals, strongly polarized towards the oxygen centers (72–89%) (Tables S16 and S18). QTAIM analysis corroborates these findings (Fig. 5b). As such, the BCPs of the Ge–O bonds exhibit low ellipticity values of *ε* = 0.046, confirming the purely σ-bonding nature in the Ge_2_O_2_ scaffold. In contrast, the BCPs of the Ge–N and Ge–C bonds display ellipticities of *ε* = 0.12 and 0.13, respectively, consistent with partial π-character and the charge delocalization into the ligand backbone.

In line with the absence of any π-bonding interaction in the Ge_2_O_2_ ring no delocalized π-symmetric molecular orbitals were observed within the four-membered ring. Nonetheless, the computed nucleus-independent chemical shifts NICS(0) and NICS(1) exhibit markedly negative values (−8.68 and −2.76 ppm, respectively), indicative of a weak magnetic shielding within the ring system. However, the relatively low NICS(1) value and the absence of a diatropic ring current indicate that compound 4a is best described as non-aromatic.

The isolated Ge(iv) µ-oxo dications 4a–e are thermally stable in the solid state at room temperature under argon atmosphere for several weeks but slowly decompose to the corresponding phosphonium salts in DCM solution over a few hours and immediately in acetonitrile, chloroform or DMSO. Attempts to cleave the dimeric structure of 4a–d by the addition of Lewis acids (GeCl_2_, ZnCl_2_, and Sc(OTf)_3_) and bases (DMAP and NH_3_) resulted in unselective reactions and the formation of multiple products. Reduction of the 1,3-digermadioxetane dications was also explored, as formation of radical species could be anticipated; however, treatment with two equivalents of cobaltocene led to uncontrolled reactions. In contrast, reduction of compound 2 with excess magnesium cleanly afforded a digermylene, which was recently reported to be formed by reduction of chlorogermylene 1 with elemental magnesium (see SI).^61^

## Conclusions

In conclusion, we have demonstrated that an ylide-substituted germanium(iv) µ-oxo chloro dimer (2) serves as a versatile platform for accessing a Lewis acid–base stabilized germaphosgene (3) and 1,3-digermadioxetane dications 4. Both compounds could be characterized in solution and in the solid state, but exhibit limited stability in solution. The dications were found to exist in solution as *syn*- and *anti*-isomers that interconvert into each other, with the equilibrium being dependent on the counter-anion. Structural analysis of germaphosgene 3 reveals a shorter Ge–O bond (1.728(1) Å) compared to the Ge–O distances in the µ-oxo dimer 2 (1.788(2)–1.857(2) Å), with a relatively large Mayer bond index of 1.11. Nonetheless, bonding analysis using DFT methods indicates an exclusive σ-bonding character of the Ge–O linkage. The same holds true for the 1,3-digermadioxetane dications, which exhibit a planar Ge_2_O_2_ square as a central structural motif with almost identical Ge–O bond lengths of *ca.* 1.79 Å.

For both compounds, the ylide ligand plays a pivotal role in accessing these reactive germanium species. While it acts as a Brønsted base that, upon protonation, converts into a neutral carbodiphosphorane ligand, which effectively binds the reactive Cl_2_Ge=O moiety in 3, it functions as a potent electron donor in the dications 4. This electron donation mitigates the increased positive charge through charge delocalization from the dicationic Ge_2_O_2_ core into the ligand backbone, resulting in a strengthening of the bonding interactions from the ylide ligand to germanium. These findings demonstrate the challenges of stabilizing multiple bonds between oxygen and the heavier group-14 elements and highlight the importance of ligand design to effectively bind and stabilize the reactive group 14 species and to enable charge delocalization.

## Author contributions

V. H. G. and M. K. conceived the project idea. M. K. performed the experimental work, spectroscopic studies, and crystallographic measurements and solved the structures. J. G. conducted the theoretical calculations and wrote the computational part. P. P. and V. H. G. helped to analyse experimental data. V. H. G. has supervised the project. The manuscript was finalized by M. K. and V. H. G. All authors have approved the final version of the manuscript.

## Conflicts of interest

There are no conflicts to declare.

## Supplementary Material

SC-OLF-D6SC04935D-s001

SC-OLF-D6SC04935D-s002

## Data Availability

CCDC 2536593–2536601 (2, 4a, 4d, 4e, 4c, 4b, 5, Y*_N_*_-Tol_-GeCl and 3) contain the supplementary crystallographic data for this paper.^65*a*–*i*^ The data that support the findings of this study are available in the supplementary information (SI). Supplementary information: experimental procedures, NMR, as well as crystallographic and computational details. See DOI: https://doi.org/10.1039/d6sc04935d. Furthermore, the raw data corresponding to the spectroscopic methods has been uploaded to an online repository and can be accessed *via*: https://doi.org/10.17877/RESOLV-2026-JJOGE4.

## References

[cit1] Barrau J., Rima G. (1998). Coord. Chem. Rev..

[cit2] Okazaki R., Tokitoh N. (2000). Acc. Chem. Res..

[cit3] Pu L., Hardman N. J., Power P. P. (2001). Organometallics.

[cit4] Nagendran S., Roesky H. W. (2008). Organometallics.

[cit5] Mizuhata Y., Sasamori T., Tokitoh N. (2009). Chem. Rev..

[cit6] Fischer R. C., Power P. P. (2010). Chem. Rev..

[cit7] Asay M., Jones C., Driess M. (2011). Chem. Rev..

[cit8] Xiong Y., Yao S., Driess M. (2013). Angew. Chem., Int. Ed..

[cit9] Loh Y. K., Aldridge S. (2021). Angew. Chem., Int. Ed..

[cit10] Sun T., Li J., Wang H. (2022). Chem. – Asian J..

[cit11] Kudo T., Nagase S. (1984). J. Phys. Chem..

[cit12] Kudo T., Nagase S. (1985). J. Am. Chem. Soc..

[cit13] Kimura M., Nagase S. (2001). Chem. Lett..

[cit14] Ellis D., Hitchcock P. B., Lappert M. F. (1992). J. Chem. Soc., Dalton Trans..

[cit15] Jissy A. K., Meena S. K., Datta A. (2013). RSC Adv..

[cit16] Tacke R., Kobelt C., Baus J. A., Bertermann R., Burschka C. (2015). Dalton Trans..

[cit17] Sen S. S., Tavčar G., Roesky H. W., Kratzert D., Hey J., Stalke D. (2010). Organometallics.

[cit18] Azhakar R., Ghadwal R. S., Roesky H. W., Wolf H., Stalke D. (2012). Chem. Commun..

[cit19] Junold K., Nutz M., Baus J. A., Burschka C., Fonseca Guerra C., Bickelhaupt F. M., Tacke R. (2014). Chem. – Asian J..

[cit20] Wang H., Xie Z. (2017). Eur. J. Inorg. Chem..

[cit21] Chlupatý T., Padělková Z., DeProft F., Willem R., Růžička A. (2012). Organometallics.

[cit22] Yao S., Brym M., van Wüllen C., Driess M. (2007). Angew. Chem., Int. Ed..

[cit23] Oshima K., Kobayashi R., Sakamoto K., Yoza K., Ishida S., Iwamoto T. (2024). Chem. – Asian J..

[cit24] Yao S., Xiong Y., Driess M. (2009). Chem. Commun..

[cit25] Yao S., Xiong Y., Wang W., Driess M. (2011). Chem. – Eur. J..

[cit26] Sinhababu S., Yadav D., Karwasara S., Sharma M. K., Mukherjee G., Rajaraman G., Nagendran S. (2016). Angew. Chem., Int. Ed..

[cit27] Jörges M., Knyszek D., Kumar M., Swamy V. S. V. S. N., Gessner V. H. (2025). Chem. Sci..

[cit28] Li L., Fukawa T., Matsuo T., Hashizume D., Fueno H., Tanaka K., Tamao K. (2012). Nat. Chem..

[cit29] Power P. P. (2012). Nat. Chem..

[cit30] Kobayashi R., Ishida S., Iwamoto T. (2019). Angew. Chem., Int. Ed..

[cit31] Tanikawa T., Ueno Y., Konaka S., Numata Y., Imai M., Ota K., Fueno H., Tanaka K., Hashizume D., Matsuo T. (2025). Chem. – Asian J..

[cit32] Ueno Y., Imai M., Maehara Y., Matsui K., Kawase Y., Ota K., Hashizume D., Matsuo T. (2025). Bull. Chem. Soc. Jpn..

[cit33] Zhao X.-X., Szilvási T., Hanusch F., Inoue S. (2021). Chem. – Eur. J..

[cit34] Wang Y., Crumpton A. E., Ellwanger M. A., McManus C., Aldridge S. (2024). Angew. Chem., Int. Ed..

[cit35] Yao S., Xiong Y., Brym M., Driess M. (2007). J. Am. Chem. Soc..

[cit36] Xiong Y., Yao S., Driess M. (2010). Angew. Chem., Int. Ed..

[cit37] Xiong Y., Yao S., Müller R., Kaupp M., Driess M. (2010). J. Am. Chem. Soc..

[cit38] Ghadwal R. S., Azhakar R., Roesky H. W., Pröpper K., Dittrich B., Klein S., Frenking G. (2011). J. Am. Chem. Soc..

[cit39] Ghadwal R. S., Azhakar R., Roesky H. W., Pröpper K., Dittrich B., Goedecke C., Frenking G. (2012). Chem. Commun..

[cit40] Rodriguez R., Gau D., Troadec T., Saffon-Merceron N., Branchadell V., Baceiredo A., Kato T. (2013). Angew. Chem., Int. Ed..

[cit41] Do D. C. H., Protchenko A. V., Ángeles Fuentes M., Hicks J., Kolychev E. L., Vasko P., Aldridge S. (2018). Angew. Chem., Int. Ed..

[cit42] Wang Y., Crumpton A. E., Ellwanger M. A., McManus C., Aldridge S. (2024). Angew. Chem., Int. Ed..

[cit43] Sharma M. K., Sinhababu S., Mahawar P., Mukherjee G., Pandey B., Rajaraman G., Nagendran S. (2019). Chem. Sci..

[cit44] Cotarca L. and Eckert H., Phosgenations – A Handbook, Wiley-VCH, 2003

[cit45] Dunlap K. L. , in Kirk-Othmer Encyclopedia of Chemical Technology, John Wiley & Sons, Ltd, 2010, pp. 1–13

[cit46] Ryan T. A. , Seddon E. A., Seddon K. R. and Ryan C., Phosgene: And Related Carbonyl Halides, Elsevier, 1996

[cit47] Lin R., Amrute A. P., Pérez-Ramírez J. (2017). Chem. Rev..

[cit48] Schneider W. and Diller W., in Ullmann's Encyclopedia of Industrial Chemistry, John Wiley & Sons, Ltd, 2000

[cit49] Babad H., Zeiler A. G. (1973). Chem. Rev..

[cit50] Scherpf T., Feichtner K.-S., Gessner V. H. (2017). Angew. Chem., Int. Ed..

[cit51] Mohapatra C., Scharf L. T., Scherpf T., Mallick B., Feichtner K.-S., Schwarz C., Gessner V. H. (2019). Angew. Chem., Int. Ed..

[cit52] Swamy V. S. V. S. N., Kumar M., Krischer F., Feichtner K.-S., Mallick B., Gessner V. H. (2024). Z. Anorg. Allg. Chem..

[cit53] Krischer F., Mayer S., Hensle L., Knyszek D., Darmandeh H., Gessner V. H. (2025). Chem. Sci..

[cit54] Lappert M. F. (1994). Main Group Met. Chem..

[cit55] Masamune S., Batcheller S. A., Park J., Davis W. M., Yamashita O., Ohta Y., Kabe Y. (1989). J. Am. Chem. Soc..

[cit56] Ellis D., Hitchcock P. B., Lappert M. F. (1992). J. Chem. Soc., Dalton Trans..

[cit57] Wang X., Peng Y., Olmstead M. M., Fettinger J. C., Power P. P. (2009). J. Am. Chem. Soc..

[cit58] Thomas H. G., Gabriel J., Fleischhauer J., Raabe G. (1983). Chem. Ber..

[cit59] Kakkar R., Chadha P., Rajni (2003). J. Mol. Struct.:THEOCHEM.

[cit60] Jörges M., Mondal S., Kumar M., Duari P., Krischer F., Löffler J., Gessner V. H. (2024). Organometallics.

[cit61] Kumar M., Remm H., Gessner V. H. (2026). J. Am. Chem. Soc..

[cit62] Holschumacher D., Bannenberg T., Hrib C. G., Jones P. G., Tamm M. (2008). Angew. Chem., Int. Ed..

[cit63] Gazis T. A., Thaker B. A. J. M., Willcox D., Ould D. M. C., Wenz J., Rawson J. M., Hill M. S., Wirth T., Melen R. L. (2020). Chem. Commun..

[cit64] Khan S., Gopakumar G., Thiel W., Alcarazo M. (2013). Angew. Chem., Int. Ed..

[cit65] (a) CCDC 2536593: Experimental Crystal Structure Determination, 2026, 10.5517/ccdc.csd.cc2r4jl1

