# Supplementary material for: A germanium µ-oxo chloro dimer en route to a heavier phosgene analogue and 1,3-digermadioxetane dications
Source: Chem Sci. 2026 Sep 15. Online ahead of print. doi: 10.1039/d6sc04935d (PMC13618331; doi:10.1039/d6sc04935d)
Supplement: Supplementary file 1 [file SC-OLF-D6SC04935D-s001.pdf]

Supplementary Information (SI) for Chemical Science.
This journal is © The Royal Society of Chemistry 2026
Supplementary Information (SI) for Chemical Science.
This journal is © The Royal Society of Chemistry

A Germanium µ-Oxo Chloro Dimer en route to a Heavier Phosgene Analogue and 1,3-Digermadioxetane Dications

Manoj Kumar, Julian Göbel, Prasenjit Palui, Viktoria H. Gessner*

Ruhr University Bochum, Faculty of Chemistry and Biochemistry, Chair of Inorganic Chemistry II, Universitätsstrasse 150, 44780 Bochum, Germany
E-mail: viktoria.gessner@rub.de

Table of Contents

1. Experimental Details **3**
1.1. General Experimental Information 3
1.2 Synthesis of **[$Y_{N\text{-}Tol}$–GeCl]** 4
1.3 Synthesis and Reduction of **2** 4
1.4 Synthesis of **3** 5
1.5 General synthetic route for the isolation of 1,3-digermadioxetane dications **(4a-e)** 8
1.6 Synthesis of **5** 13
2 NMR spectra of the isolated compounds **15**
2.1 NMR spectra of compound **[$Y_{N\text{-}Tol}$–GeCl]** 15
2.2 NMR spectra of compound **2** 16
2.3 NMR spectra of compound **3** 18
2.4 NMR spectra of compounds **4a**–**e** 21
2.5 NMR spectra of compound **5** 32
3. Crystal structure determination. **34**
3.1 General information 34
3.2. Crystal structure analysis of the chlorogermylene, **2**, and **6** 38
3.3. Crystal structure analyses of the 1,3-digermadioxetane dications **4a-e** 41
3.4. Crystal structure analysis of compound **5** 46
4. Computational results **47**
4.1 General information 47
4.2 Energy optimization 47
4.3 Molecular Orbitals 50
4.4 NBO analysis 52
4.5 IBO analysis 55
4.6 QTAIM analysis 58
4.7 XYZ coordinates 60
5. References **71**

The original data obtained from all analysis methods have been deposited in a repository and can be downloaded via the following link. The only exceptions are the data from the single-crystal X-ray diffraction experiments. The CIF files for these experiments have been deposited with the Cambridge Crystallographic Data Centre (CCDC) (see chapter 3 for details).

Link: https://doi.org/10.17877/RESOLV-2026-JJOGE4

1. Experimental Details

1.1. General Experimental Information

Chemicals and conditions. All experiments (if not stated otherwise) were carried out under a dry, oxygen-free argon atmosphere using standard Schlenk techniques. Argon (99.999%) was purchased from Air Liquide. Solvents and chemicals: Involved solvents were dried using a MBraun SPS **7** (THF, toluene, diethyl ether, n-hexane, n-pentane, acetonitrile) or dried in accordance with standard procedures and stored under an argon atmosphere over 3 Å or 4 Å molecular sieves. Reagents were purchased from Sigma-Aldrich, ABCR, Acros Organics, or TCI Chemicals and used without further purification if not stated otherwise. BnK, $B(C_6F_5)_3$, $H_2O{\cdot}B(C_6F_5)_3$, [$Y_{N\text{-}Tol}$-H], [$Y_{N\text{-}Tol}$-K], [$Y_{N\text{-}Mes}$-H], and [$Y_{N\text{-}Mes}$-GeCl] were synthesized following literature procedures.[1–5]

NMR spectroscopy: ^{1}H, $^{13}C\{^{1}H\}$, $^{19}F\{^{1}H\}$, $^{11}B\{^{1}H\}$, and $^{31}P\{^{1}H\}$ NMR spectra were recorded on Avance-III-400 spectrometers at 22 ° to –45 °C if not stated otherwise. All values of the chemical shift are in ppm regarding the δ-scale. All spin-spin coupling constants (*J*) are printed in Hertz (Hz). To display multiplicities and signal forms correctly the following abbreviations were used: s = singlet, d = doublet, t = triplet, m = multiplet, dd = doublet of doublet, br = broad signal. Signal assignment was supported by, HSQC (^{1}H / ^{13}C), HMBC (^{1}H / ^{13}C, ^{1}H / ^{31}P) correlation experiments.

Elemental analyses were performed in the in-house facility on an Elementar vario MICRO cube elemental analyzer.

For details about the single-crystal X-ray diffraction analyses, see Chapter 3.

Caution! Strong bases such as organolithium bases, especially as neat compounds, are severely air-moisture-sensitive and pyrophoric organometallic compounds. These compounds need to be handled under an inert gas atmosphere to exclude reactions with oxygen and water. Guidelines for their handling can be found in literature: T. L. Rathman, J. A. Schwindeman, *Org. Process Res. Dev.* **2014**, *18*, 1192.

1.2 Synthesis of [$Y_{N\text{-}Tol}$–GeCl]

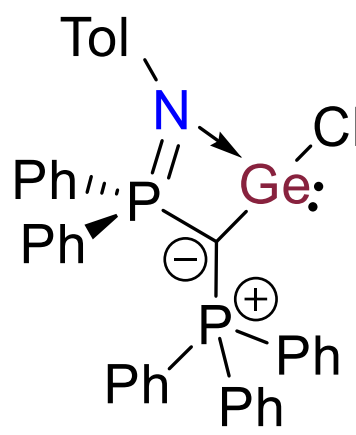

To a mixture of **$Y_{N\text{-}Tol}$-H** (200 mg,1 equiv, 0.35 mmol) and benzyl potassium (46.1 mg, 1 equiv, 0.35 mmol) was added 3 mL dry toluene at room temperature. The suspension turned dark red over 5 min and was stirred for another 10 min. A pre-cooled (–30 °C) solution of $GeCl_2$·dioxane (73 mg, 0.35 mmol, 1.0 equiv) in thf (1 mL) was added The color of the reaction mixture changed immediately to yellow from dark red. After stirring for 10 min, solvents were removed under reduced pressure. The yellow solid was extracted with toluene (10 mL). Single crystals suitable for X-ray diffraction analyses were grown by slow vapor diffusion of hexane into a solution of the compound in toluene at room temperature. After drying the crystals under *vacuo*, the yield was 80% (190 mg, 0.28 mmol).

$^{31}P\{^1H\}$-NMR (162 MHz, Benzene-d_6): δ = 37.97 (d, $^2J_{PP}$ = 43.0 Hz, ***P***Ph_2N), 13.48 (d, $^2J_{PP}$ = 43.0 Hz, ***P***Ph_3) ppm.

^1H-NMR (400 MHz, Benzene-d_6): δ = 8.08 (br, 2H. $NPPh_2$***H***$_{Ph,\ para}$), 7.53 (dd, J =12.8 Hz, 7.2 Hz, 8H, $NPPh_2$***H***$_{Ph,\ ortho,\ meta}$), 7.14 (s, 2H, *p*-Tol***H***$_{Ph,\ ortho}$) 6.94 – 6.80 (m, 17H, PPh_3***H***$_{Ph,\ ortho,\ meta,\ para}$; *p*-Tol***H***$_{Ph,\ meta}$), 2.00 (s, 3H, *p*-Tol-C***H***$_3$) ppm.

$^{13}C\{^1H\}$-NMR (101 MHz, Benzene-d_6): δ = 143.13 (*p*-Tol***C***$H_{Ph,\ ipso}$), 133.33 (d, $^2J_{CP}$ = 10.6 Hz, $NPPh_2$***C***$_{Ph,\ ortho}$), 133.07 (br, $NPPh_2$***C***$_{Ph,\ ipso}$), 132.03 (br, $NPPh_2$***C***$_{Ph,\ para}$), 131.49 (d, $^3J_{CP}$ = 2.8 Hz, PPh_3***C***$_{Ph,\ meta}$), 130.28 (d, $^3J_{CP}$ = 5.7 Hz, $NPPh_2$***C***$_{Ph,\ meta}$), 129.83 (s, *p*-Tol***C***$H_{Ph,\ ortho}$), 129.42 (s, *p*-Tol***C***$H_{Ph,\ para}$), 128.71 (d, $^3J_{CP}$ = 12.2 Hz, PPh_3***C***$_{Ph,\ ortho}$), 128.57 (s, PPh_3***C***$_{Ph,\ para}$), 118.82 (d, $^2J_{CP}$ = 11.8 Hz, *p*-Tol***C***$H_{Ph,\ ortho}$), 20.85 (s, *p*-Tol-***C***H_3) ppm; P***C***P and PPh_3***C***$_{Ph,\ ipso}$ not observed.

Anal. Calcd for $C_{38}H_{32}Cl_1Ge_1N_1P_2$ × (KCl): C, 61.07; H, 4.31; N, 1.87. Found: C, 61.95; H, 4.76; N, 1.84.

1.3 Synthesis and Reduction of 2

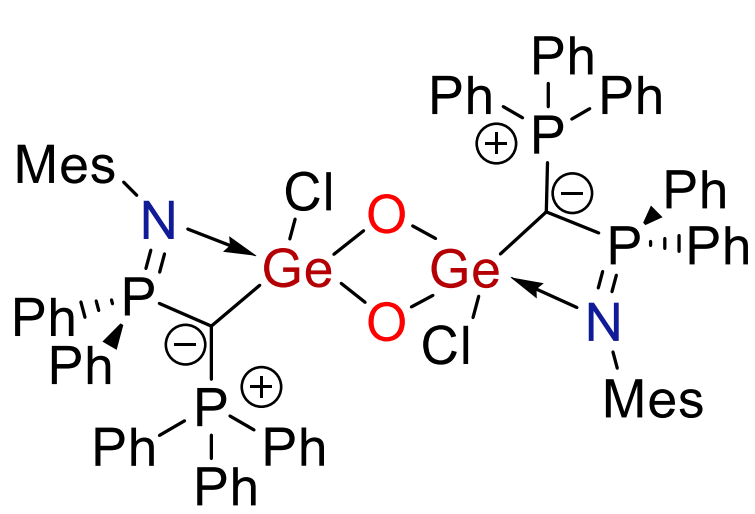

[{$Y_{N\text{-}Mes}$–GeOCl}$_2$]: 603 mg (1 eq., 1.02 mmol) **$Y_{N\text{-}Mes}$–H** and 199 mg benzyl potassium (1.5 eq., 1.53 mmol) were dissolved in 10 mL toluene and were stirred for 30 min. Then the cold solution at –30 °C of 236 mg (1 eq., 0.33 mmol) $GeCl_2$·dioxane in thf (5 mL) was added. The color of the reaction mixture changed immediately to yellow from dark red. The solution was stirred for another 30 min. Afterwards, the reaction mixture was filtered. The solution was freeze-pump-thawed and degassed before being exposed to 1 bar of N_2O at room temperature. The reaction mixture was stirred for overnight. Next day, the white residue was observed of the desired compound. All volatiles were removed under reduced pressure, and the resulting off-white solid was washed with 2 × 5 mL of hexane and dried. Yield: 1.2 g, 0.83 mmol, 82 %. Single crystals suitable for X-ray diffraction analyses were grown by slow vapor diffusion of hexane into a solution of the compound in DCM at room temperature.

^{31}P{^{1}H}-NMR (162 MHz, DCM-d$_2$): δ = 38.31 – 35.68 (br, ***P***Ph$_2$N), 11.46 (d, $^2J_{PP}$ = 36.4 Hz, ***P***Ph$_3$) ppm.

^{1}H-NMR (400 MHz, DCM-d$_2$): δ = 7.53 – 7.46 (m, 8H, NPPh$_2$***H***$_{Ph, ortho,}$ PPh$_3$***H***$_{Ph, ortho}$), 7.44 – 7.40 (m, 4H, NPPh$_2$***H***$_{Ph, meta}$), 7.26 – 7.20 (m, 4H, NPPh$_2$***H***$_{Ph, para,}$ PPh$_3$***H***$_{Ph, ortho}$) 7.17 – 7.12 (m, 9H, PPh$_3$***H***$_{Ph, meta, para}$), 6.67 (s, 2H, mes-***H***$_{Ph, meta}$), 2.27 (s, 3H, mes-C***H***$_{3\ Ph, para}$), 1.62 (s, 6H, mes-C***H***$_{3\ Ph, ortho}$) ppm.

^{13}C{^{1}H}-NMR (101 MHz, DCM-d$_2$): δ = 138.80 (d, $^2J_{CP}$ = 4.5 Hz mes-***C***$_{Ph, ortho}$), 136.43 (br, mes-***C***$_{Ph, ipso}$), 135.23 (s, mes-***C***$_{Ph, para}$), 133.74 (d, $^2J_{CP}$ = 10.8 Hz, NPPh$_2$***C***$_{Ph, ortho}$), 133.32 (d, $^2J_{CP}$ = 10.7 Hz, PPh$_3$***C***$_{Ph, ortho}$),132.86 (s, PPh$_3$***C***$_{Ph, para}$) 132.59 (s, NPPh$_2$***C***$_{Ph, ipso}$), 129.34 (d, $^3J_{CP}$ = 2.7 Hz mes-***C***$_{Ph, meta}$), 129.15 (d, $^3J_{CP}$ = 12.8 Hz, NPPh$_2$***C***$_{Ph, meta}$), 128.71 (s, NPPh$_2$***C***$_{Ph, para}$), 128.40 (d, $^3J_{CP}$ = 12.5 Hz, PPh$_3$***C***$_{Ph, meta}$), 126.80 (dd, $^1J_{CP}$ = 93.5, 3.5Hz, PPh$_3$***C***$_{Ph, ipso}$) 22.12 (s,mes-*C*H$_{3\ Ph, para}$), 21.07 (s,mes-*C*H$_{3\ Ph, ortho}$) ppm; P***C***P not observed.

Anal. Calcd for $C_{80}H_{72}Cl_2Ge_2N_2O_2P_4$ × (CH_2Cl_2): C, 64.07; H, 4.91; N, 1.84. Found: C, 64.00; H, 4.91; N, 2.03.

Reduction Experiment: Mg turnings (2.5 mg, 3 eq., 0.10 mmol) were added to thee solution of **2** (50 mg, 1 eq., 0.03 mmol) in 2 mL THF and sonicated for 14 hours. The reaction mixture gradually darkened to form a dark red suspension. The ^{31}P{^{1}H} NMR spectrum of the reaction mixture confirmed the complete consumption of **2** and formation of product along with magnesium yldiide species. Removal of all volatiles under reduced pressure, led to a red sloid, which was dissolved in 1mL of diethylether. Unreacted Mg turnings was removed *via* filteration through celite. Concentration of the resulting dark red solution at room temperature for 1 hour, yielded few deep red crystals of digermylene. The spectroscopic data of the isolated product are in full agreement with those previously reported.[3]

1.4 Synthesis of 3

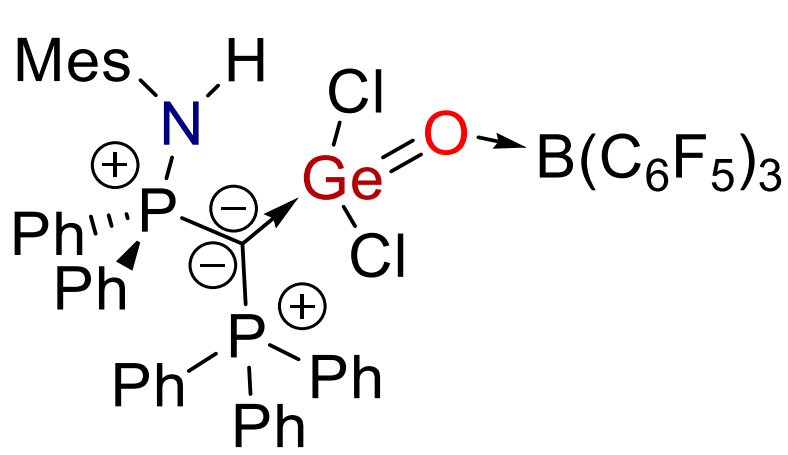

A pre-cooled solution (–30 °C) of $H_2O{\cdot}B(C_6F_5)_3$ (132 mg, 0.25 mmol, 2.0 equiv.) in thf (1 mL) was added dropwise to a suspension of compound **2** (178.6 mg, 0.12 mmol, 1.0 equiv.) in thf (3 mL). The resulting mixture was stirred for 30 minutes at room temperature. Removal of the solvent under reduced pressure from the reaction mixture resulted in a white solid. The white solid was washed with cold thf (2 × 2 mL) and dried under *vacuo*. Yield: 67% (105 mg, 0.08 mmol). This yield is calculated based on 1 equivalent of **3** per equivalent of dimer **2** (the theoretical maximum, since only one germanium center per dimer can retain both chlorine atoms required to form **3**); expressed relative to the total Ge content of **2**, this corresponds to ca. 33% Ge-atom yield.

Single crystals suitable for X-ray diffraction analyses could be grown by two methods: 1) slow vapor diffusion of hexane into a solution of the compound in toluene at room temperature. 2) by layering the solution of the compound in thf with pentane at room temperature.
After isolation, analytically pure compound **3** was found to be insoluble in ethereal solvents and decomposed immediately upon dissolution in acetonitrile, chloroform, or DMSO. Although the compound is shortly stable for handling in DCM, it decomposes slowly during NMR characterization in DCM-d_2 at room temperature to several compounds, amongst the protonated ligand [δ = 32.1 (d, $^2J_{PP}$ = 31.4 Hz, **P**Ph_2N) and 19.3 (d, $^2J_{PP}$ = 31.4 Hz, **P**Ph_3)] at room temperature (see Figure S11). In the solid state, compound **3** is stable for weeks when stored under an argon atmosphere.

$^{31}P\{^1H\}$-NMR (162 MHz, DCM-d_2): δ = 38.04 (d,$^2J_{PP}$ = 33.5 Hz, ***P***Ph_2N), 21.38 (d, $^2J_{PP}$ = 33.4 Hz, ***P***Ph_3) ppm.
^1H-NMR (400 MHz, DCM-d_2): δ = 7.76 (d, $^2J_{NP}$ = 10.1Hz, 1H, mes-N***H***) 7.58 – 7.48j (m, 12H), 7.43 (t, J = 7.1Hz, 3H), 7.36 – 7.34 (m, 5H), 7.29 – 7.24 (m, 3H), 7.21 – 7.17 (m, 2H), 6.45 (s, 2H, mes***H***$_{Ph,\ meta}$), 2.08 (s, 3H, mes-C***H***$_{3\ Ph,\ para}$), 1.40 (s, 6H, mes-C***H***$_{3\ Ph,\ ortho}$) ppm.

$^{13}C\{^1H\}$-NMR (101 MHz, DCM-d_2): δ = 147.14 (dm, $^1J_{CF}$ = 235.2 Hz, $B(C_6F_5)_3$-***C***$_{Ph,\ ortho}$),138.07 (dm, $^1J_{CF}$ = 235.26 Hz, $B(C_6F_5)_3$-***C***$_{Ph,\ para}$), 135.79 (dm, $^1J_{CF}$ = 235.2 Hz, $B(C_6F_5)_3$-***C***$_{Ph,\ meta}$), 137.13 (d, $^5J_{CP}$ = 3.1 Hz mes-***C***$_{Ph,\ para}$), 135.22 (d, J = 10.1 Hz, $NPPh_2$***C***$_{Ph,\ meta}$), 134.73 (d, $^4J_{CP}$ = 10.1 Hz, PPh_3***C***$_{Ph,\ meta}$), 134.44 (d, $^2J_{CP}$ = 10.7 Hz, $NPPh_2$***C***$_{Ph,\ ortho}$), 134.29 (d, J = 10.2 Hz, PPh_3***C***$_{Ph,\ orthokj}$), 133.28 (br, s, PPh_3***C***$_{Ph,\ para}$), 131.18 (br, s, mes-***C***$_{Ph,\ ipso}$), 129.40 (d, $^3J_{CP}$ = 7.4 Hz mes-***C***$_{Ph,\ ortho}$), 129.57 (br, s, $NPPh_2$***C***$_{Ph,\ para}$), 129.12 (d, $^4J_{CP}$ = 12.7 Hz mes-***C***$_{Ph,\ meta}$), 128.57 – 128.23 (m, PPh_3***C***$_{Ph,\ ipso}$), 127.86 – 126.50 (m, $NPPh_2$***C***$_{Ph,\ ipso}$), 20.67 (s, mes-***C***$H_{3\ Ph,\ para}$), 19.25 (s, mes-***C***$H_{3\ Ph,\ ortho}$) ppm; [$B(C_6F_5)_3$-***C***$_{Ph,\ ipso}$], and P***C***P not observed.

$^{19}F\{^1H\}$-NMR (377 MHz, DCM-d_2): δ = –133.34 (d, $^3J_{FF}$ = 21.0 Hz, 6F, C_6***F***$_{5,\ ortho}$), –163.10 (t, $^3J_{FF}$ = 20.3 Hz, 6F, C_6***F***$_{5,\ para}$), –167.23 (t, $^3J_{FF}$ = 19.2 Hz, 6F, $B(C_6$***F***$_5)_{3,\ meta}$) ppm.

$^{11}B\{^1H\}$-NMR (128 MHz, DCM-d_2): δ = –3.91 (br, ***B***$(C_6F_5)_3$) ppm.

Anal. Calcd for $C_{58}H_{37}B_1Cl_2F_{15}Ge_1N_1O_1P_2$: C, 55.06; H, 2.95; N, 1.11. Found: C, 55.22; H, 3.02; N, 1.16.

Proposed mechanism for the formation of **3**

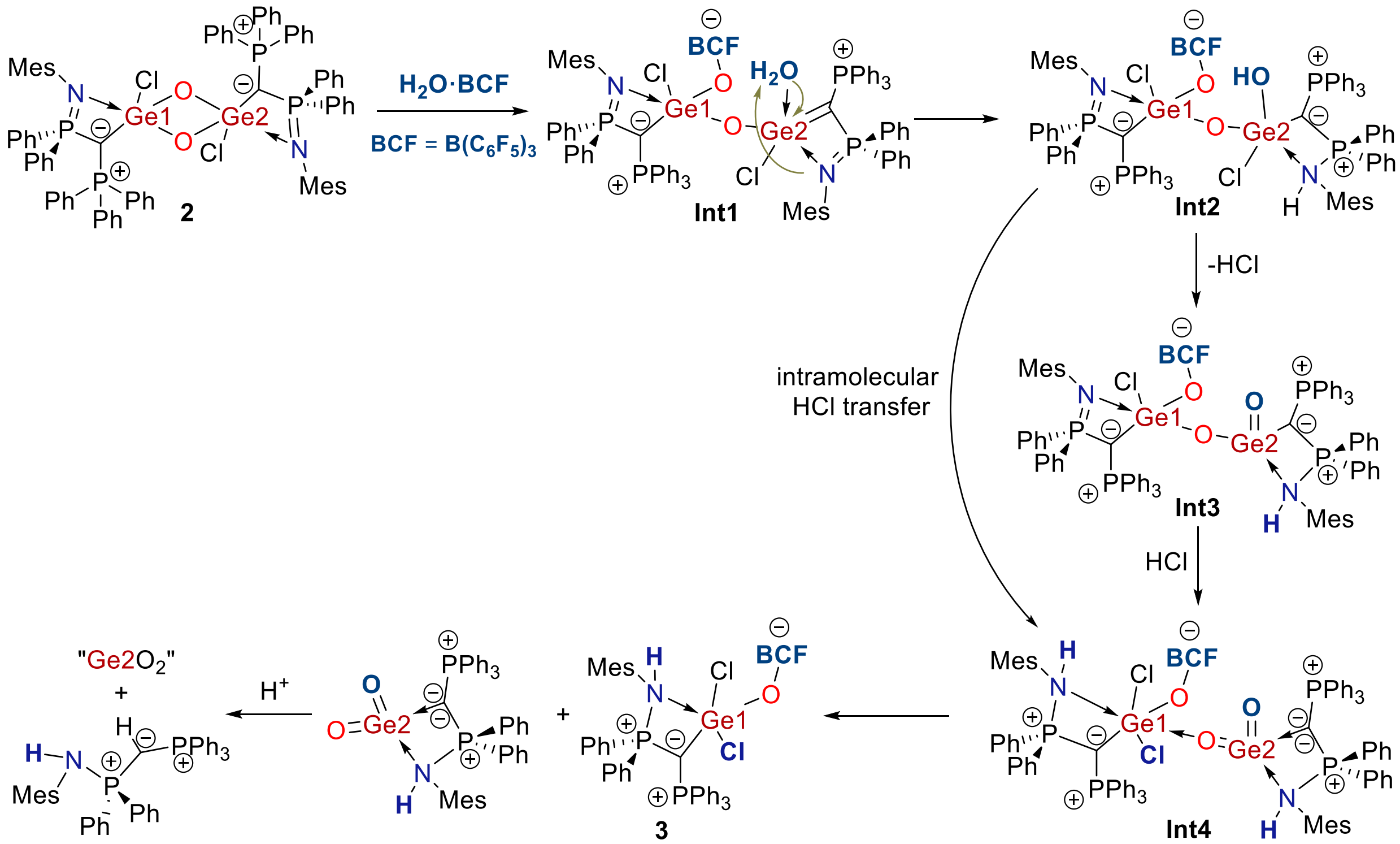

Scheme 1. Proposed reaction mechanism for the formation of germaphosgene **3** (BCF = $B(C_6F_5)_3$).

The formation of compound **3** is proposed to begin with the reaction of precursor **2** with the H_2O·BCF adduct. Formal addition of H_2O·BCF to one of the Ge–O bonds generates intermediate **Int1**, which is expected to undergo a proton transfer from the germanium-bound water to the coordinated ligand. The resulting species, **Int2**, can eliminate HCl from the newly formed Cl–Ge2–OH fragment. This elimination may occur intramolecularly, giving either intermediate **Int4** directly or proceeding through a second intermediate, **Int3**, before affording **Int4**.

Intermediate **Int4** already incorporates the final product **3**; it loosely coordinates a germanium-oxo fragment that can readily decoordinate. The germanium-oxo unit is highly labile and can readily polymerise to give oligomeric germanium-oxo species. Residual H_2O·BCF (or HCl) can protonate the ligand, producing the corresponding phosphonium salt, which could be identified by NMR spectroscopy [δ = 32.07 (d, $^{2}J_{PP}$ = 31.4 Hz, PPh_2N) and 19.34 (d, $_{2}J_{PP}$ = 31.4 Hz, PPh_3)] and confirmed by X-ray crystallography as its chloride salt. However, we would like to emphasize that the counter anion of the protonated ligand does not necessarily have to be chloride under the reaction conditions. Because an excess (2 equiv.) H_2O·BCF was found to provide the best conversion in the reaction process, proton transfer from H_2O·BCF to the ligand can also generate hydroxyborate as counteranion.

It is noteworthy that protonation of **Int3** may also take place at the ligand bound to the second germanium centre (Ge2), leading to immediate ligand cleavage and the formal elimination of "GeO_2." It is also worth noting that the simple addition of HCl to **2** does **not** generate a germaphosgene stabilized by $GeCl_2$. Instead, HCl causes complete cleavage of the ylide ligands,

producing the fully protonated ligand as a dicationic bis-phosphonium salt accompanied by $GeCl_3^-$ and Cl^- counter-anions (see the synthesis of compound **5**, page S13). This result demonstrates that water is required to trigger the formation of the germaphosgene species.

1.5 General synthetic route for the isolation of 1,3-digermadioxetane dications (4a-e)

The sodium/silver salts [$NaBAr^F_4$ (for compound **4a**, $AgSbF_6$ (for compound **4b**), and AgOTf (for compound **4c**)] and $B(C_6F_5)_3$ (for compound **4d**) were transferred to a pre-cooled (–30 °C) solution of compound **2** in DCM. The reaction mixture was stirred for 10 minutes at room temperature and then filtered. Removal of the solvent under reduced pressure from the filtrate gave an analytically pure sample of the 1,3-digermadioxetane dications; $[\{Y_{N\text{-}Mes}–GeO\}_2]^{2+}$ $2[BAr^F]^-$ (**4a**), $[\{Y_{N\text{-}Mes}–GeO\}_2]^{2+}$ $2[SbF_6]^-$ (**4b**), $[\{Y_{N\text{-}Mes}–GeO\}_2]^{2+}$ $2[OTf]^-$ (**4c**) and $[\{Y_{N\text{-}Mes}–GeO\}_2]^{2+}$ $2[B(C_6F_5)_3Cl]^-$ (**4d**).

Synthesis of compound (4a); $[\{Y_{N\text{-}Mes}–GeO\}_2]^{2+}$ $2[BAr^F_4]^-$

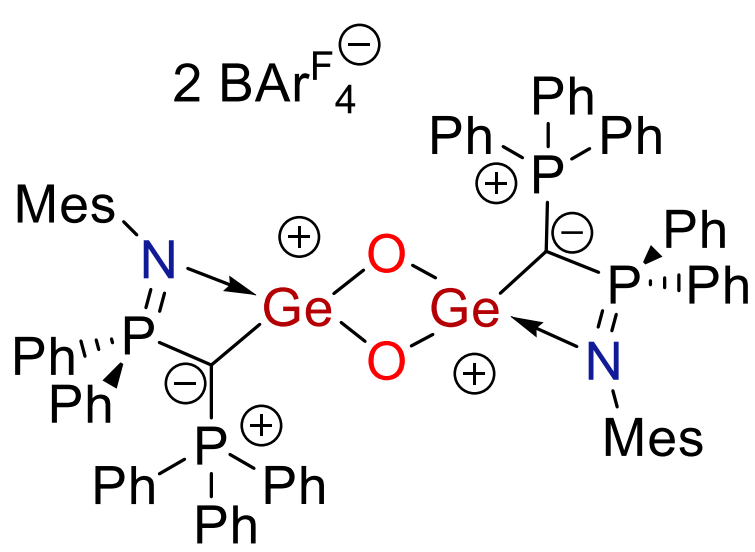

Compound **2** (74 mg, 0.05 mmol, 1 equiv.), $NaBAr^F_4$ (91.5 mg, 0.10 mmol, 2 equiv.). Single crystals suitable for X-ray diffraction analyses were grown by layering hexane into a solution of the compound in DCM at –30 °C. Yield: 69 % (110.2 mg, 0.04 mmol).

*Both the anti and syn isomer are observed by NMR spectroscopy, in a ratio of 55:45, respectively. Over time, crystals of compound **4a** were observed to be unstable in solution and gradually decomposed to the corresponding phosphonium salt, as indicated by the appearance of new resonances in the $^{31}P\{^1H\}$ NMR spectrum δ = [δ_P: 33.74 (d, $^2J_{PP}$ = 31.85 Hz, **P**Ph$_2$N, 19.28 (d, $^2J_{PP}$ = 31.85 Hz, **P**Ph$_3$)]. Due to the close proximity of signals in the aromatic region of the ^1H and ^{13}C NMR spectra, the unambiguous assignment of anti and syn isomers is not possible.*

$^{31}P\{^1H\}$-NMR (162 MHz, DCM-d$_2$) for *anti* isomer: δ = 46.17 (d, $^2J_{PP}$ = 26.9 Hz, **P**Ph$_2$N), 13.44 (d, $^2J_{PP}$ = 27.0 Hz, **P**Ph$_3$, *anti*) ppm.

$^{31}P\{^1H\}$-NMR (162 MHz, DCM-d$_2$) for *syn* isomer: δ = 45.61 (d, $^2J_{PP}$ = 25.5 Hz, **P**Ph$_2$N, *syn*), 13.71 (d, $^2J_{PP}$ = 25.5 Hz, **P**Ph$_3$, *syn*) ppm.

^1H-NMR (400 MHz, DCM-d$_2$) for *anti + syn*: δ = 7.73 (br m, 8H, BAr$^F_4$-**H**$_{, ortho}$), 7.55 (br s, 4H, BAr^F-**H**$_{, para}$), 7.54 – 7.50 (m, 3H, *anti* + *syn*), 7.44 – 7.41 (m, 3H, *anti* + *syn*), 7.36 – 7.34 (m, 6H, *anti* + *syn*), 7.32 – 7.31 (br, 2H, *anti* + *syn*), 7.27 – 7.17 (m, 12H, *anti* + *syn*), 6.30 (s, 2H, mes**H**$_{Ph,}$

$_{meta}$, *anti*), 6.27 (s, 2H, mes$\underline{\boldsymbol{H}}_{Ph,\ meta}$, *syn*), 2.07 (s, 3H, mes-C$\underline{\boldsymbol{H}}_{3\ Ph,\ para}$, *anti*), 1.94 (s, 3H, mes-C$\underline{\boldsymbol{H}}_{3\ Ph,\ para}$, *syn*), 1.53 (s, 6H, mes-C$\underline{\boldsymbol{H}}_{3\ Ph,\ ortho}$, *syn*),1.51 (s, 6H, mes-C$\underline{\boldsymbol{H}}_{3\ Ph,\ ortho}$, *anti*) ppm.

^{13}C{^{1}H}-NMR (101 MHz, DCM-d$_2$) for *anti* + *syn*: δ = 162.19 (q, ^{1}J = 50 Hz, BAr^{F}-$\underline{\boldsymbol{C}}_{Ph,\ ipso}$), 137.15 (d, $^{5}J_{CP}$ = 4.0 Hz mes-$\underline{\boldsymbol{C}}_{Ph,\ para}$, *anti*),137.03 (d, $^{5}J_{CP}$ = 4.0 Hz mes-$\underline{\boldsymbol{C}}_{Ph,\ para}$, *syn*), 135.23 (br m, BAr^{F}-$\underline{\boldsymbol{C}}_{Ph,\ ortho}$),134.88 (d, J = 3.0 Hz, NPPh$_2$$\underline{\boldsymbol{C}}_{Ph,\ meta}$, *syn*), 134.82 (d, J = 3.0 Hz, NPPh$_2$$\underline{\boldsymbol{C}}_{Ph,\ meta}$, *anti*), 134.59 (d, $^{4}J_{CP}$ = 2.9 Hz, PPh$_3$$\underline{\boldsymbol{C}}_{Ph,\ meta}$, *syn*), 134.50 (d, $^{4}J_{CP}$ = 3.3 Hz, PPh$_3$$\underline{\boldsymbol{C}}_{Ph,\ meta}$, *anti*), 130.70 (d, $^{2}J_{CP}$ = 13.2 Hz mes-$\underline{\boldsymbol{C}}_{Ph,\ meta}$), 130.27 (d, $^{3}J_{CP}$ = 12.9 Hz mes-$\underline{\boldsymbol{C}}_{Ph,\ ortho}$, *anti*), 130.07 (d, $^{3}J_{CP}$ = 12.9 Hz mes-$\underline{\boldsymbol{C}}_{Ph,\ ortho}$, *syn*), 129.z75 (br, s, mes-$\underline{\boldsymbol{C}}_{Ph,\ ipso}$), 129.61 (br, s, NPPh$_2$$\underline{\boldsymbol{C}}_{Ph,\ para}$), 129.52 (br, s, PPh$_2$$\underline{\boldsymbol{C}}_{Ph,\ para}$, *anti*), 129.43 (br, s, NPPh$_2$$\underline{\boldsymbol{C}}_{Ph,\ ortho}$), 129.30 (br, s, PPh$_3$$\underline{\boldsymbol{C}}_{Ph,\ ortho}$), 129.16 (m, BAr^{F}-$\underline{\boldsymbol{C}}_{Ph,\ meta}$), 125.01 (d, ^{1}J = 272.4 Hz, BAr^{F}-$\underline{\boldsymbol{C}}F_3$) 124.00 (d, $^{1}J_{CP}$ = 96.8 Hz, PPh$_3$$\underline{\boldsymbol{C}}_{Ph,\ ipso}$, *anti*), 123.96 (d, $^{1}J_{CP}$ = 93.9 Hz, NPPh$_2$$\underline{\boldsymbol{C}}_{Ph,\ ipso}$, *syn*) 123.89 (d, $^{1}J_{CP}$ = 93.9 Hz, NPPh$_2$$\underline{\boldsymbol{C}}_{Ph,\ ipso}$, *anti*), 123.53 (d, $^{1}J_{CP}$ = 94.0, 3.0 Hz, PPh$_3$$\underline{\boldsymbol{C}}_{Ph,\ ipso}$, *syn*), 117.86 (sept, ^{4}J = 3.6 Hz, BAr^{F}-$\underline{\boldsymbol{C}}_{Ph,\ para}$), 21.06 (s, mes-$\underline{\boldsymbol{C}}H_{3\ Ph,\ para}$, *anti*) , 20.63 (s, mes-$\underline{\boldsymbol{C}}H_{3\ Ph,\ para}$, *syn*), 19.79 (s, mes-$\underline{\boldsymbol{C}}H_{3\ Ph,\ ortho}$, *syn*), 19.38 (s, mes-$\underline{\boldsymbol{C}}H_{3\ Ph,\ ortho}$, *anti*) ppm; P$\underline{\boldsymbol{C}}$P not observed.

^{19}F{^{1}H}-NMR (377 MHz, DCM-d$_2$): δ = –62.83 (s, [{(C$\underline{\boldsymbol{F}}_3$)$_2C_6H_3$}$_4$B]) ppm.

^{11}B{^{1}H}-NMR (128 MHz, DCM-d$_2$): δ = –6.54 – 6.63 (m, [{(CF$_3$)$_2$C$_6$H$_3$}$_4$$\underline{\boldsymbol{B}}$]) ppm.

Anal. Calcd for $C_{144}H_{96}B_2F_{48}Ge_2N_2O_2P_4$: C, 55.99; H, 3.13; N, 0.91. Found: C, 55.09; H, 2.99; N, 0.97.

Synthesis of compound (4b); [{Y$_{N\text{-}Mes}$–GeO}$_2$]$^{2+}$ 2[SbF$_6$]$^{–}$

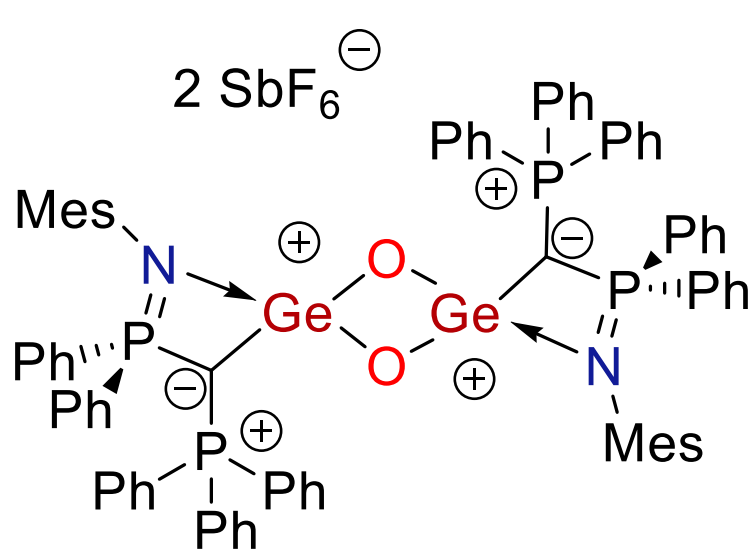

Compound **2** (234.3 mg, 0.16 mmol, 1 equiv.), AgSbF$_6$ (112.3 mg, 0.32 mmol, 2 equiv.). Single crystals suitable for X-ray diffraction analyses were grown by layering hexane into a solution of the compound in DCM at –30 °C. Yield: 66 % (197 mg, 0.10 mmol).

Both the anti and syn isomer are observed by NMR spectroscopy, in a ratio of 62:38, respectively. Over time, crystals of compound ***4b*** *were observed to be unstable in solution and gradually decomposed to the corresponding phosphonium salt, as indicated by the appearance of new resonances in the ^{31}P{^{1}H} NMR spectrum δ = [δ$_P$: 33.7 (d, $^{2}J_{PP}$ = 31.85 Hz,* ***P̲***Ph$_2$N*, 19.3 (d, $^{2}J_{PP}$ = 31.85 Hz,* ***P̲***Ph$_3$*)]. Due to the close proximity of signals in the aromatic region of the ^{1}H and ^{13}C NMR spectra, the unambiguous assignment of anti and syn isomers is not possible.*

^{31}P{^{1}H}-NMR (162 MHz, DCM-d$_2$) for *anti* isomer: δ = 46.11 (d,$^{2}J_{PP}$ = 26.9 Hz, ***P***Ph$_2$N), 13.43 (d, $^{2}J_{PP}$ = 26.7 Hz, ***P***Ph$_3$, *anti*) ppm.

^{31}P{^{1}H}-NMR (162 MHz, DCM-d$_2$) for *syn* isomer: δ = 45.62 (d,$^{2}J_{PP}$ = 25.6 Hz, ***P***Ph$_2$N, *syn*), 13.67 (d, $^{2}J_{PP}$ = 25.6 Hz, ***P***Ph$_3$, *syn*) ppm.

^{1}H-NMR (400 MHz, DCM-d$_2$) for *anti* + *syn*: δ = 7.73 (br m, 8H, BAr^{F}-***H***$_{, ortho}$), 7.55 (br s, 4H, BAr^{F}-***H***$_{, para}$), 7.54 – 7.50 (m, 3H, *anti* + *syn*), 7.44 – 7.41 (m, 3H, *anti* + *syn*), 7.36 – 7.34 (m, 6H, *anti* + *syn*), 7.32 – 7.31 (br, 2H, *anti* + *syn*), 7.27 – 7.17 (m, 12H, *anti* + *syn*), 6.30 (s, 2H, mes***H***$_{Ph, meta}$, *anti*), 6.27 (s, 2H, mes***H***$_{Ph, meta}$, *syn*), 2.07 (s, 3H, mes-C***H***$_{3\ Ph, para}$, *anti*), 1.94 (s, 3H, mes-C***H***$_{3\ Ph, para}$, *syn*), 1.53 (s, 6H, mes-C***H***$_{3\ Ph, ortho}$, *syn*),1.51 (s, 6H, mes-C***H***$_{3\ Ph, ortho}$, *anti*) ppm.

^{13}C{^{1}H}-NMR (101 MHz, DCM-d$_2$) for *anti* + *syn*: δ = 137.26 (d, $^{5}J_{CP}$ = 3.9 Hz mes-***C***$_{Ph, para}$, *anti*), 137.11 (d, $^{5}J_{CP}$ = 3.7 Hz mes-***C***$_{Ph, para}$, *syn*), 134.88 (d, J = 2.9 Hz, NPPh$_2$***C***$_{Ph, meta}$, *syn*), 134.78 (d, J = 2.9 Hz, NPPh$_2$***C***$_{Ph, meta}$, *anti*), 134.61 (d, $^{4}J_{CP}$ = 2.9 Hz, PPh$_3$***C***$_{Ph, meta}$, *syn*), 134.56 (d, $^{4}J_{CP}$ = 3.0 Hz, PPh$_3$***C***$_{Ph, meta}$, *anti*), 133.50 (d, $^{2}J_{CP}$ = 11.3 Hz, NPPh$_2$***C***$_{Ph, ortho}$, *anti*), 133.44 (d, $^{2}J_{CP}$ = 11.2 Hz, NPPh$_2$***C***$_{Ph, ortho}$, *syn*), 133.09 (d, J = 10.9 Hz, PPh$_3$***C***$_{Ph, ortho}$, *anti*), 132.85 (d, J = 10.9 Hz, PPh$_3$***C***$_{Ph, ortho}$, *syn*), 131.15 (d, $^{4}J_{CP}$ = 5.7 Hz PPh$_3$***C***$_{Ph, para}$), 130.79 (d, $^{2}J_{CP}$ = 13.4 Hz mes-***C***$_{Ph, ipso}$), 130.35 (d, $^{3}J_{CP}$ = 12.9 Hz mes-***C***$_{Ph, ortho}$, *anti*), 130.12 (d, $^{3}J_{CP}$ = 12.9 Hz mes-***C***$_{Ph, ortho}$, *syn*), 129.47 (d, $^{4}J_{CP}$ = 10.5 Hz mes-***C***$_{Ph, meta}$, *anti*), 129.46 (br, s, NPPh$_2$***C***$_{Ph, para}$), 129.32 (d, $^{4}J_{CP}$ = 10.5 Hz mes-***C***$_{Ph, meta}$, *anti*), 124.44 (d, $^{1}J_{CP}$ = 93.9 Hz, NPPh$_2$***C***$_{Ph, ipso}$), 123.85 (dd, $^{1}J_{CP}$ = 94.3, 3.0 Hz, PPh$_3$***C***$_{Ph, ipso}$, *anti*), 123.94 (dd, $^{1}J_{CP}$ = 94.5, 3.0 Hz, PPh$_3$***C***$_{Ph, ipso}$, *syn*), 21.08 (s, mes-***C***H$_{3\ Ph, para}$, *anti*) , 20.68 (s, mes-***C***H$_{3\ Ph, para}$, *syn*), 19.85 (s, mes-***C***H$_{3\ Ph, ortho}$, *syn*), 19.45 (s, mes-***C***H$_{3\ Ph, ortho}$, *anti*) ppm; P***C***P not observed.

Anal. Calcd for $C_{80}H_{72}F_{12}Ge_2N_2O_2P_4Sb_2$ × 2 (CH_2Cl_2): C, 49.14; H, 3.82; N, 1.40. Found: C, 49.79; H, 3.97; N, 1.46.

Synthesis of compound (4c); [{Y$_{N\text{-}Mes}$–GeO}$_2$]$^{2+}$ 2[OTf]$^{–}$

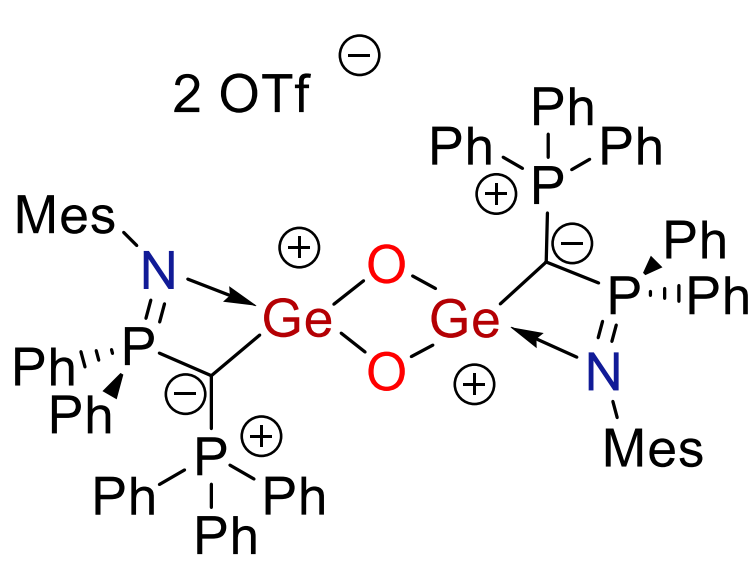

Compound **2** (273 mg, 0.19 mmol, 1 equiv.), AgOTf (97.9 mg, 0.38 mmol, 2 equiv.). Single crystals suitable for X-ray diffraction analyses were grown by layering hexane into a solution of the compound in DCM at –30 °C. Yield: 88% (279 mg, 0.17 mmol).

Only the *anti* isomer is observed by NMR spectroscopy.

^{31}P{^{1}H}-NMR (162 MHz, DCM-d$_2$) at room temperature: δ = 45.92 (d,$^{2}J_{PP}$ = 26.4 Hz, ***P***Ph$_2$N), 13.54 (d, $^{2}J_{PP}$ = 26.4 Hz, ***P***Ph$_3$) ppm.

^{31}P{^{1}H}-NMR (162 MHz, DCM-d$_2$) at –45 °C (*anti*): δ = 46.36 (d,$^{2}J_{PP}$ = 27.7 Hz, ***P***Ph$_2$N), 13.06 (d, $^{2}J_{PP}$ = 27.9 Hz, ***P***Ph$_3$) ppm.

^{31}P{^{1}H}-NMR (162 MHz, DCM-d$_2$) at –45 °C (*syn*): δ = 45.59 (d,$^{2}J_{PP}$ = 26.3 Hz, ***P***Ph$_2$N), 13.41 (d, $^{2}J_{PP}$ = 26.4 Hz, ***P***Ph$_3$) ppm.

^{1}H-NMR (400 MHz, DCM-d$_2$): δ = 7.61 – 7.53 (m, 5H), 7.44 – 7.40 (m, 6H), 7.34 – 7.27 (m, 10H), 7.25 – 7.20 (m, 4H), 6.30 (s, 2H, mes***H***$_{Ph,\ meta}$), 2.03 (s, 3H, mes-C***H***$_{3\ Ph,\ para}$), 1.53 (s, 6H, mes-C***H***$_{3\ Ph,\ ortho}$) ppm.

^{13}C{^{1}H}-NMR (101 MHz, DCM-d$_2$): δ = 137.22 (s, mes-***C***$_{Ph,\ ipso}$), 137.20 (s, mes-***C***$_{Ph,\ para}$), 134.87 (d, $^{4}J_{CP}$ = 3.0 Hz, NPPh$_2$***C***$_{Ph,\ meta}$), 134.64 (d, $^{4}J_{CP}$ = 3.1 Hz, PPh$_3$***C***$_{Ph,\ meta}$), 133.49 (d, ^{3}J = 11.3 Hz, NPPh$_2$***C***$_{Ph,\ ortho}$), 133.01 (d, ^{3}J = 10.8 Hz, PPh$_3$***C***$_{Ph,\ ortho}$), 131.38 (br, PPh$_3$***C***$_{Ph,\ para}$), 130.31 (d, $^{3}J_{CP}$ = 12.9 Hz, mes-***C***$_{Ph,\ ortho}$), 129.75 (s, NPPh$_2$***C***$_{Ph,\ para}$), 129.48 (d, $^{4}J_{CP}$ = 13.1 Hz mes-***C***$_{Ph,\ meta}$), 124.46 (d, $^{1}J_{CP}$ = 94.1 Hz, NPPh$_2$***C***$_{Ph,\ ipso}$), 123.91 (dd, $^{1}J_{CP}$ = 93.5, 3.0 Hz, PPh$_3$***C***$_{Ph,\ ipso}$), 20.95 (s, mes-***C***H$_{3\ Ph,\ para}$), 19.62 (s, mes-***C***H$_{3\ Ph,\ ortho}$) ppm; P***C***P and ***C***F$_3$O$_3$S not observed.

^{19}F{^{1}H}-NMR (377 MHz, DCM-d$_2$): δ = –78.76 (s,C***F***$_3$O$_3$S) ppm.

Anal. Calcd for $C_{82}H_{72}F_6Ge_2N_2O_8P_4S_2$ × (CH_2Cl_2): C, 57.10; H, 4.27; N, 1.60; S, 3.67. Found: C, 57.10; H, 4.51; N, 1.55; S, 3.34.

Synthesis of compound (4d); [{Y$_{N\text{-}Mes}$–GeO}$_2$]$^{2+}$ 2[B(C$_6$F$_5$)$_3$Cl]$^{-}$

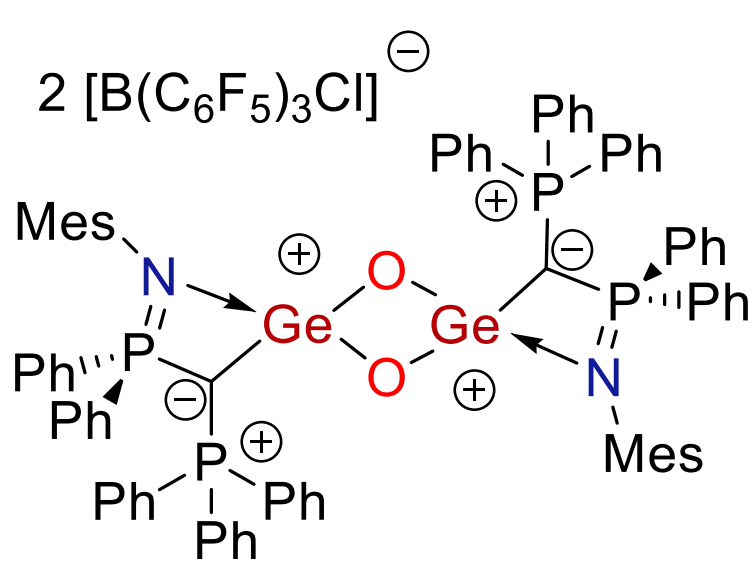

Compound **2** (169 mg, 0.11 mmol, 1 equiv.), B(C$_6$F$_5$)$_3$(123 mg, 0.23 mmol, 2 equiv.). Single crystals suitable for X-ray diffraction analyses were grown by layering hexane into a solution of the compound in toluene at room temperature. Yield: 71 % (207 mg, 0.08 mmol).

Both the anti and syn isomer are observed by NMR spectroscopy, in a ratio of 57:43, respectively. Due to the close proximity of signals in the aromatic region of the ^{1}H and ^{13}C NMR spectra, the unambiguous assignment of anti and syn isomers is not possible.

^{31}P{^{1}H}-NMR (162 MHz, DCM-d$_2$) for *anti* isomer: δ = 45.95 (d,$^{2}J_{PP}$ = 26.9 Hz, ***P***Ph$_2$N), 13.27 (d, $^{2}J_{PP}$ = 26.9 Hz, ***P***Ph$_3$, *anti*) ppm.

^{31}P{^{1}H}-NMR (162 MHz, DCM-d$_2$) for *syn* isomer: δ = 45.45 (d,$^{2}J_{PP}$ = 25.6 Hz, ***P***Ph$_2$N, *syn*), 13.50 (d, $^{2}J_{PP}$ = 25.6 Hz, ***P***Ph$_3$, *syn*) ppm.

^{1}H-NMR (400 MHz, DCM-d$_2$) for *anti + syn*: δ = 7.73 (br m, 8H, BAr^{F}-$\underline{\boldsymbol{H}}_{,\ ortho}$), 7.55 (br s, 4H, BAr^{F}-$\underline{\boldsymbol{H}}_{,\ para}$), 7.54 – 7.50 (m, 3H, *anti* + *syn*), 7.44 – 7.41 (m, 3H, *anti* + *syn*), 7.36 – 7.34 (m, 6H, *anti* + *syn*), 7.32 – 7.31 (br, 2H, *anti* + *syn*), 7.27 – 7.17 (m, 12H, *anti* + *syn*), 6.30 (s, 2H, mes$\underline{\boldsymbol{H}}_{Ph,\ meta}$, *anti*), 6.27 (s, 2H, mes$\underline{\boldsymbol{H}}_{Ph,\ meta}$, *syn*), 2.07 (s, 3H, mes-C$\underline{\boldsymbol{H}}_{3\ Ph,\ para}$, *anti*), 1.94 (s, 3H, mes-C$\underline{\boldsymbol{H}}_{3\ Ph,\ para}$, *syn*), 1.53 (s, 6H, mes-C$\underline{\boldsymbol{H}}_{3\ Ph,\ ortho}$, *syn*),1.51 (s, 6H, mes-C$\underline{\boldsymbol{H}}_{3\ Ph,\ ortho}$, *anti*) ppm.

^{13}C{^{1}H}-NMR (101 MHz, DCM-d$_2$) for *anti + syn*: δ = 147.34 (dm, $^{1}J_{CF}$ = 237 Hz, B(C$_6$F$_5$)$_3$-$\underline{\boldsymbol{C}}_{Ph,\ ortho}$), 138.01 (dm, $^{1}J_{CF}$ = 237 Hz, B(C$_6$F$_5$)$_3$-$\underline{\boldsymbol{C}}_{Ph,\ para}$), 135.80 (dm, $^{1}J_{CF}$ = 237 Hz, B(C$_6$F$_5$)$_3$-$\underline{\boldsymbol{C}}_{Ph,\ meta}$), 137.20 (d, $^{5}J_{CP}$ = 4.0 Hz mes-$\underline{\boldsymbol{C}}_{Ph,\ para}$, *anti*), 137.06 (d, $^{5}J_{CP}$ = 3.8 Hz mes-$\underline{\boldsymbol{C}}_{Ph,\ para}$, *syn*), 134.88 (d, J = 3.0 Hz, NPPh$_2$$\underline{\boldsymbol{C}}_{Ph,\ meta}$, *syn*), 134.79 (d, J = 2.9 Hz, NPPh$_2$$\underline{\boldsymbol{C}}_{Ph,\ meta}$, *anti*), 134.58 (d, $^{4}J_{CP}$ = 2.9 Hz, PPh$_3$$\underline{\boldsymbol{C}}_{Ph,\ meta}$, *syn*), 134.50 (d, $^{4}J_{CP}$ = 3.0 Hz, PPh$_3$$\underline{\boldsymbol{C}}_{Ph,\ meta}$, *anti*), 133.77 (d, $^{4}J_{CP}$ = 10.7 Hz PPh$_3$$\underline{\boldsymbol{C}}_{Ph,\ para}$), 133.48 (d, $^{2}J_{CP}$ = 11.3 Hz, NPPh$_2$$\underline{\boldsymbol{C}}_{Ph,\ ortho}$, *anti*), 133.44 (d, $^{2}J_{CP}$ = 11.4 Hz, NPPh$_2$$\underline{\boldsymbol{C}}_{Ph,\ ortho}$, *syn*), 133.05 (d, J = 10.9 Hz, PPh$_3$$\underline{\boldsymbol{C}}_{Ph,\ ortho}$, *anti*), 132.82 (d, J = 10.6 Hz, PPh$_3$$\underline{\boldsymbol{C}}_{Ph,\ ortho}$, *syn*), 131.07 (dd, $^{2}J_{CP}$ = 63.0, 5.1 Hz mes-$\underline{\boldsymbol{C}}_{Ph,\ ipso}$, *anti*), 131.00 (dd, $^{2}J_{CP}$ = 63.0, 5.1 Hz mes-$\underline{\boldsymbol{C}}_{Ph,\ ipso}$, *syn*), 130.29 (d, $^{3}J_{CP}$ = 12.9 Hz mes-$\underline{\boldsymbol{C}}_{Ph,\ ortho}$, *anti*), 130.07 (d, $^{3}J_{CP}$ = 12.8 Hz mes-$\underline{\boldsymbol{C}}_{Ph,\ ortho}$, *syn*), 129.48 (d, $^{4}J_{CP}$ = 9.9 Hz mes-$\underline{\boldsymbol{C}}_{Ph,\ meta}$, *anti*), 129.35 (d, $^{4}J_{CP}$ = 9.8 Hz mes-$\underline{\boldsymbol{C}}_{Ph,\ meta}$, *anti*), 129.15(d, $^{5}J_{CP}$ = 12.7 Hz, NPPh$_2$$\underline{\boldsymbol{C}}_{Ph,\ para}$), 128.40(d, $^{5}J_{CP}$ = 12.5 Hz, NPPh$_2$$\underline{\boldsymbol{C}}_{Ph,\ para}$),124.44 (d, $^{1}J_{CP}$ = 94.0 Hz, NPPh$_2$$\underline{\boldsymbol{C}}_{Ph,\ ipso}$, *syn*), 124.39 (d, $^{1}J_{CP}$ = 94.0 Hz, NPPh$_2$$\underline{\boldsymbol{C}}_{Ph,\ ipso}$, *anti*), 123.50 (dd, $^{1}J_{CP}$ = 94.5, 3.0 Hz, PPh$_3$$\underline{\boldsymbol{C}}_{Ph,\ ipso}$, *anti*), 123.46 (dd, $^{1}J_{CP}$ = 94.5, 3.0 Hz, PPh$_3$$\underline{\boldsymbol{C}}_{Ph,\ ipso}$, *syn*), 21.05 (s, mes-$\underline{\boldsymbol{C}}H_{3\ Ph,\ para}$, *anti*) , 20.62 (s, mes-$\underline{\boldsymbol{C}}H_{3\ Ph,\ para}$, *syn*), 19.78 (s, mes-$\underline{\boldsymbol{C}}H_{3\ Ph,\ ortho}$, *syn*), 19.35 (s, mes-$\underline{\boldsymbol{C}}H_{3\ Ph,\ ortho}$, *anti*) ppm; P$\underline{\boldsymbol{C}}$P and B(C$_6F_5$)$_3$-$\underline{\boldsymbol{C}}_{Ph,\ ipso}$] not observed.

^{19}F{^{1}H}-NMR (377 MHz, DCM-d$_2$): δ = –132.73 (d, $^{3}J_{FF}$ = 20.4 Hz, 6F, C$_6$$\underline{\boldsymbol{F}}_{5,\ ortho}$), –162.52 (t, $^{3}J_{FF}$ = 20.4 Hz, 6F, C$_6$$\underline{\boldsymbol{F}}_{5,\ para}$), –167.23 (t, $^{3}J_{FF}$ = 22.5 Hz, 6F, B(C$_6$$\underline{\boldsymbol{F}}_5$)$_{3,\ meta}$) ppm.

^{11}B{^{1}H}-NMR (128 MHz, DCM-d$_2$): δ = –7.36 (br, B(C$_6$$\underline{\boldsymbol{F}}_5$)$_3$) ppm.
Anal. Calcd for $C_{116}H_{72}B_2Cl_2F_{30}Ge_2N_2O_2P_4$ × 0.5 (CH_2Cl_2): C, 55.97; H, 2.94; N, 1.12. Found: C, 55.11; H, 2.76; N, 1.36.

Synthesis of compound (4e); [{Y$_{N\text{-}Mes}$–GeO}$_2$]$^{2+}$ 2[B(C$_6$F$_5$OTf)]$^{–}$

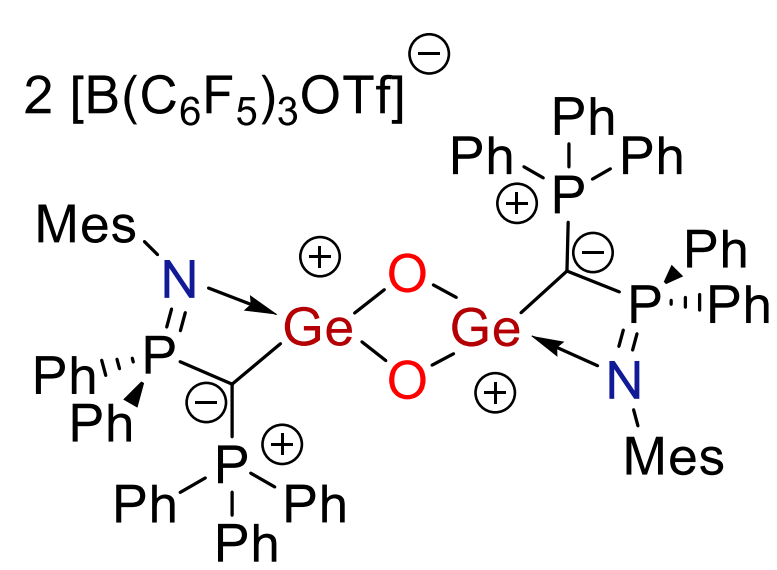

Method 1: A pre-cooled solution (–30 °C) of compound **4c** (42.3 mg, 0.02 mmol, 1 equiv.) in toluene (1 mL) was transferred to B(C$_6$F$_5$)$_3$ (26.1 mg, 0.05 mmol, 2 equiv.). In 15 minutes, the starting material was consumed, as confirmed by ^{31}P and ^{19}F NMR spectroscopy. Removal of solvent under reduced pressure from the reaction mixture resulted in a white solid. The white solid was washed with hexane (2 × 1 mL) and dried under *vacuo*. Yield: 36% (52.8 mg, 0.01 mmol). Single crystals suitable for X-ray diffraction analyses were grown by layering hexane into a solution of the compound in toluene at room temperature.
*Over time, crystals of compound **4e** were observed to be unstable in solution and gradually decomposed to the corresponding phosphonium salt, as indicated by the appearance of new*

*resonances in the $^{31}P\{^{1}H\}$ NMR spectrum δ = [δ_P: 33.74 (d, $^{2}J_{PP}$ = 31.85 Hz, **P**Ph_2N, 19.28 (d, $^{2}J_{PP}$ = 31.85 Hz, **P**Ph_3)].*

Method 2: To a pre-cooled solution (–30 °C) of **4d** (30.6 mg, 0.01 mmol, 1 equiv.) in DCM, AgOTf (6.4, 0.02 mmol, 2 equiv.) was added. The reaction mixture was stirred for 15 minutes and then filtered. Removal of all volatiles from the filtrate under reduced pressure resulted in an analytically pure sample of compound **4e**.

^{31}P{^{1}H}-NMR (162 MHz, DCM-d$_2$): δ = 45.95 (d, $^{2}J_{PP}$ = 26.1 Hz, ***P***Ph_2N), 13.58 (d, $^{2}J_{PP}$ = 26.3 Hz, ***P***Ph_3) ppm.

^{1}H-NMR (400 MHz, DCM-d$_2$): δ = 7.62 – 7.55 (m, 5H), 7.45 – 7.40 (m, 7H), 7.35 – 7.29 (m, 8H), 7.26 – 7.21 (m, 5H), 6.31 (s, 2H, mes***H***$_{Ph,\ meta}$), 2.04 (s, 3H, mes-C***H***$_{3\ Ph,\ para}$), 1.54 (s, 6H, mes-C***H***$_{3\ Ph,\ ortho}$) ppm.

^{13}C{^{1}H}-NMR (101 MHz, DCM-d$_2$): δ = 147.13 (dm, $^{1}J_{CF}$ = 237 Hz, B(C_6F_5)$_3$-***C***$_{Ph,\ ortho}$), 137.18 (d, $^{5}J_{CP}$ = 3.7 Hz mes-***C***$_{Ph,\ para}$), 134.85 (d, J = 2.9 Hz, NPPh$_2$***C***$_{Ph,\ meta}$), 134.60 (d, $^{4}J_{CP}$ = 2.9 Hz, PPh$_3$***C***$_{Ph,\ meta}$), 133.46 (d, $^{2}J_{CP}$ = 11.3 Hz, NPPh$_2$***C***$_{Ph,\ ortho}$), 132.98 (d, J = 10.9 Hz, PPh$_3$***C***$_{Ph,\ ortho}$), 131.32 (br, s, PPh$_3$***C***$_{Ph,\ para}$), 130.71 (d, $^{2}J_{CP}$ = 13.5 Hz mes-***C***$_{Ph,\ ipso}$), 130.27 (d, $^{3}J_{CP}$ = 12.9 Hz mes-***C***$_{Ph,\ ortho}$), 129.73 (br, s, NPPh$_2$***C***$_{Ph,\ para}$), 129.44 (d, $^{4}J_{CP}$ = 13.1 Hz mes-***C***$_{Ph,\ meta}$), 124.41 (d, $^{1}J_{CP}$ = 93.9 Hz, NPPh$_2$***C***$_{Ph,\ ipso}$), 123.88 (dd, $^{1}J_{CP}$ = 94.4, 2.9 Hz, PPh$_3$***C***$_{Ph,\ ipso}$), 20.91 (s, mes-***C***H$_{3\ Ph,\ para}$), 19.59 (s, mes-***C***H$_{3\ Ph,\ ortho}$) ppm; [B(C_6F_5)$_3$-***C***$_{Ph,\ meta,\ para,\ ipso}$], P***C***P and ***C***$F_3O_3S$ not observed.

^{19}F{^{1}H}-NMR (377 MHz, DCM-d$_2$): δ = –132.95 (d, $^{3}J_{FF}$ = 18.9 Hz, 6F, C$_6$***F***$_{5,\ ortho}$), –160.35 (br s, C$_6$***F***$_{5,\ para}$), –166.34 (br s, B(C$_6$***F***$_5$)$_{3,\ meta}$), –78.74 (s, ***F***$_3O_3S$) ppm.
^{11}B{^{1}H}-NMR (128 MHz, DCM-d$_2$): δ = –0.42 (br, ***B***(C_6F_5)$_3$) ppm.

Anal. Calcd for $C_{118}H_{72}B_2F_{36}Ge_2N_2O_8P_4S_2$: C, 52.79; H, 2.70; N, 1.04; S, 2.39. Found: C, 50.63; H, 3.28; N, 0.99; S, 1.77.

1.6 Synthesis of 5

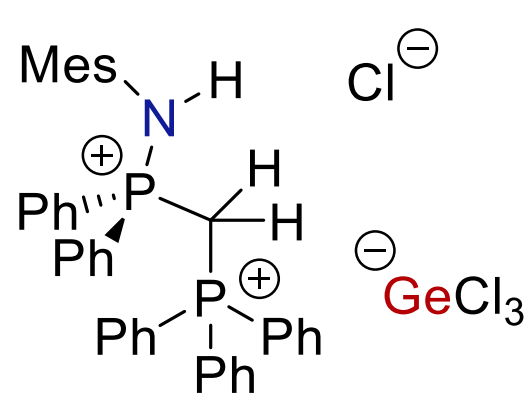

A solution of HCl (4 M in 1,4-dioxane, 0.04 mL, 2.0 equiv) in thf (3 mL) was added dropwise to a stirring solution of compound **2** (104 mg, 0.07 mmol, 1.0 equiv) in anhydrous DCM (3 mL) at **–**78 °C under a nitrogen atmosphere. The resulting mixture was stirred at **–**78 °C for 20 minutes and allowed to warm to room temperature. Subsequently, the solvent was removed under reduced pressure. The white solid was washed with pentane (2 mL) and dried under *vacuo*. Yield: 94 % (55 mg, 0.07 mmol). Single crystals suitable for X-ray diffraction analyses were grown by slow vapor diffusion of hexane into a solution of the compound in DCM at room temperature.

^{31}P{^{1}H}-NMR (162 MHz, DCM-d$_2$): δ = 35.47 (d, $^{2}J_{PP}$ = 14.1 Hz, ***P***Ph$_2$N), 17.35 (d, $^{2}J_{PP}$ = 14.0 Hz, ***P***Ph$_3$) ppm.

^{1}H-NMR (400 MHz, DCM-d$_2$): δ = 9.82 (d, $^{1}J_{NP}$ = 7.3 Hz, 1H, mes-N***H***), 8.02 (dd, *J* = 13.4, 7.6 Hz, 4H, NPPh$_2$***H***$_{Ph, ortho}$), 7.92 (dd, *J* = 13.4, 7.4 Hz, 6H, NPPh$_2$***H***$_{Ph, meta,}$ PPh$_3$***H***$_{Ph, ortho}$), 7.64 – 7.58 (m, 4H), 7.53 (br d, *J* = 7.5 Hz, 2H, PC***H***P), 7.50 – 7.45 (m, 7H), 7.37 – 7.32 (m, 4H), 6.58 (s, 2H, mes***H***$_{Ph, meta}$), 2.17 (s, 6H, mes-C***H***$_{3\ Ph, ortho}$), 2.05 (s, 3H, mes-C***H***$_{3\ Ph, para}$) ppm.

^{13}C{^{1}H}-NMR (101 MHz, DCM-d$_2$): δ = 137.11 (d, $^{5}J_{CP}$ = 2.7 Hz mes-***C***$_{Ph, para}$), 137.00 (d, $^{3}J_{CP}$ = 3.7 Hz, mes-***C***$_{Ph, ortho}$), 135.68 (d, $^{3}J_{CP}$ = 3.0 Hz, NPPh$_2$***C***$_{Ph, meta}$), 135.22 (d, $^{3}J_{CP}$ = 3.1 Hz, PPh$_3$***C***$_{Ph, meta}$), 134.91 (d, $^{2}J_{CP}$ = 11.5 Hz, NPPh$_2$***C***$_{Ph, ortho}$), 134.33 (d, $^{2}J_{CP}$ = 10.9 Hz, PPh$_3$***C***$_{Ph, ortho}$),130.37 (d, $^{4}J_{CP}$ = 13.3 Hz, PPh$_3$***C***$_{Ph, para}$), 129.65 (d, $^{4}J_{CP}$ = 13.6 Hz NPPh$_2$***C***$_{Ph, para}$), 131.33 (d, $^{2}J_{CP}$ = 3.8 Hz, mes-***C***$_{Ph, ipso}$), 129.45 (d, $^{4}J_{CP}$ = 1.9 mes-***C***$_{Ph, meta}$),118.40 (d, $^{2}J_{CP}$ = 102.5, NPPh$_2$***C***$_{Ph, ipso}$), 118.00 (d, $^{1}J_{CP}$ = 88.2, PPh$_3$***C***$_{Ph, ipso}$), 22.97 (dd, $^{1}J_{CP}$ = 47.0, 57.6 Hz, P***C***P), 21.02 (s, mes-*C*H$_{3\ Ph, ortho}$), 20.73 (s, mes-*C*H$_{3\ Ph, para}$), ppm.

2 NMR spectra of the isolated compounds

2.1 NMR spectra of compound [$Y_{N\text{-}Tol}$–GeCl]

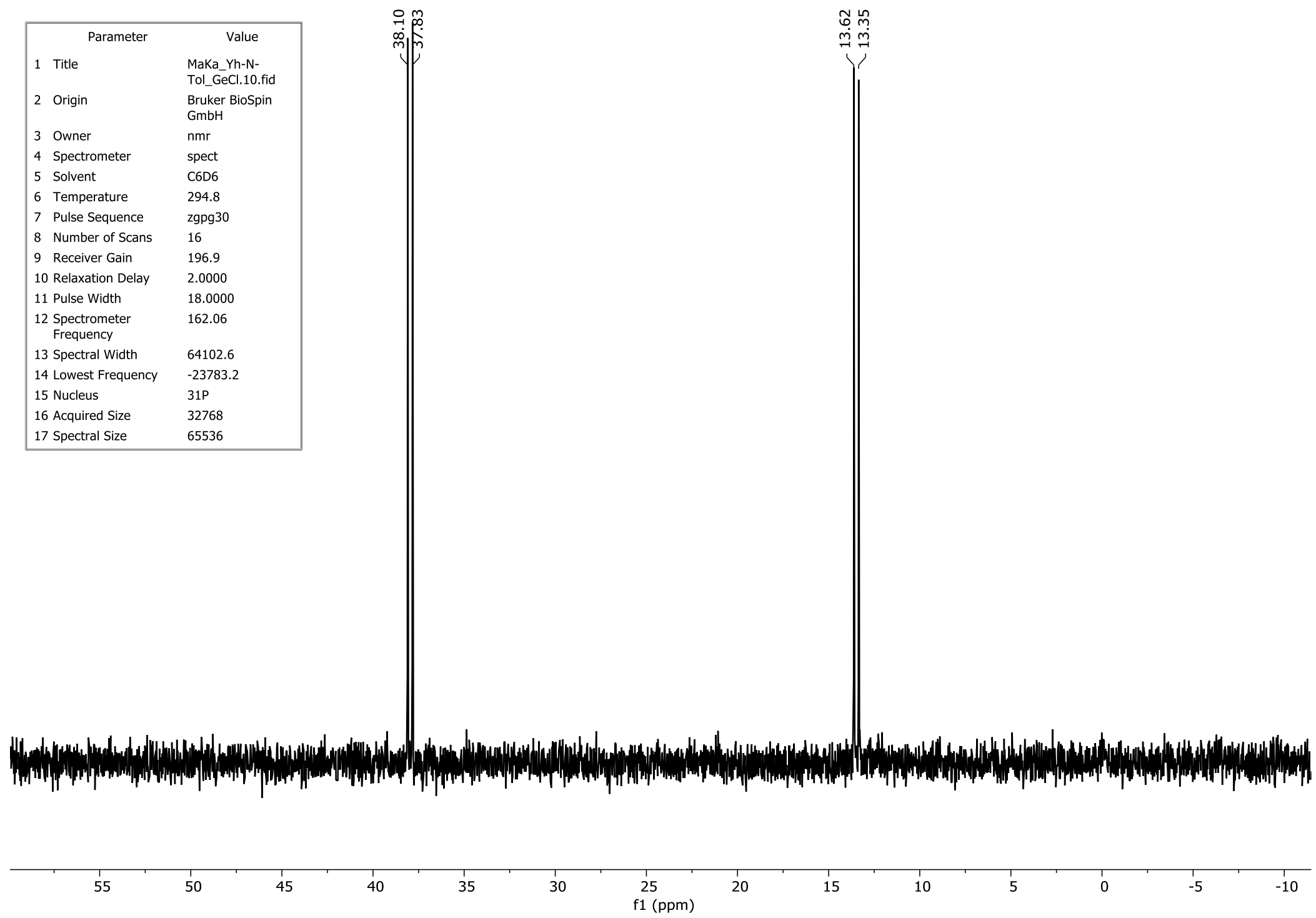

Figure S1. $^{31}P\{^{1}H\}$ NMR spectrum of compound **[$Y_{N\text{-}Tol}$–GeCl]** in Benzene-d_6.

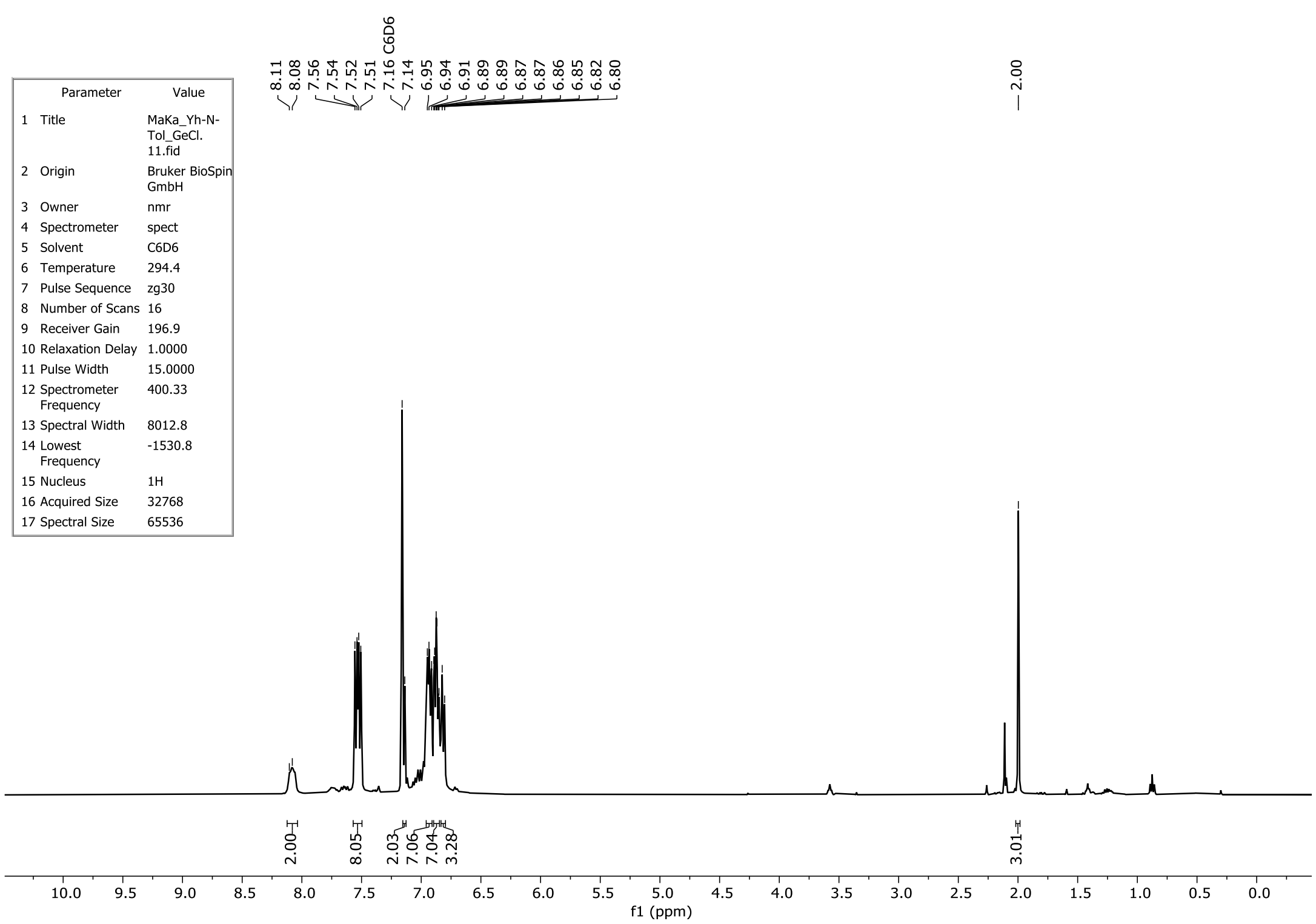

Figure S2. ^{1}H NMR spectrum of compound **[$Y_{N\text{-}Tol}$–GeCl]** in Benzene-d_6.

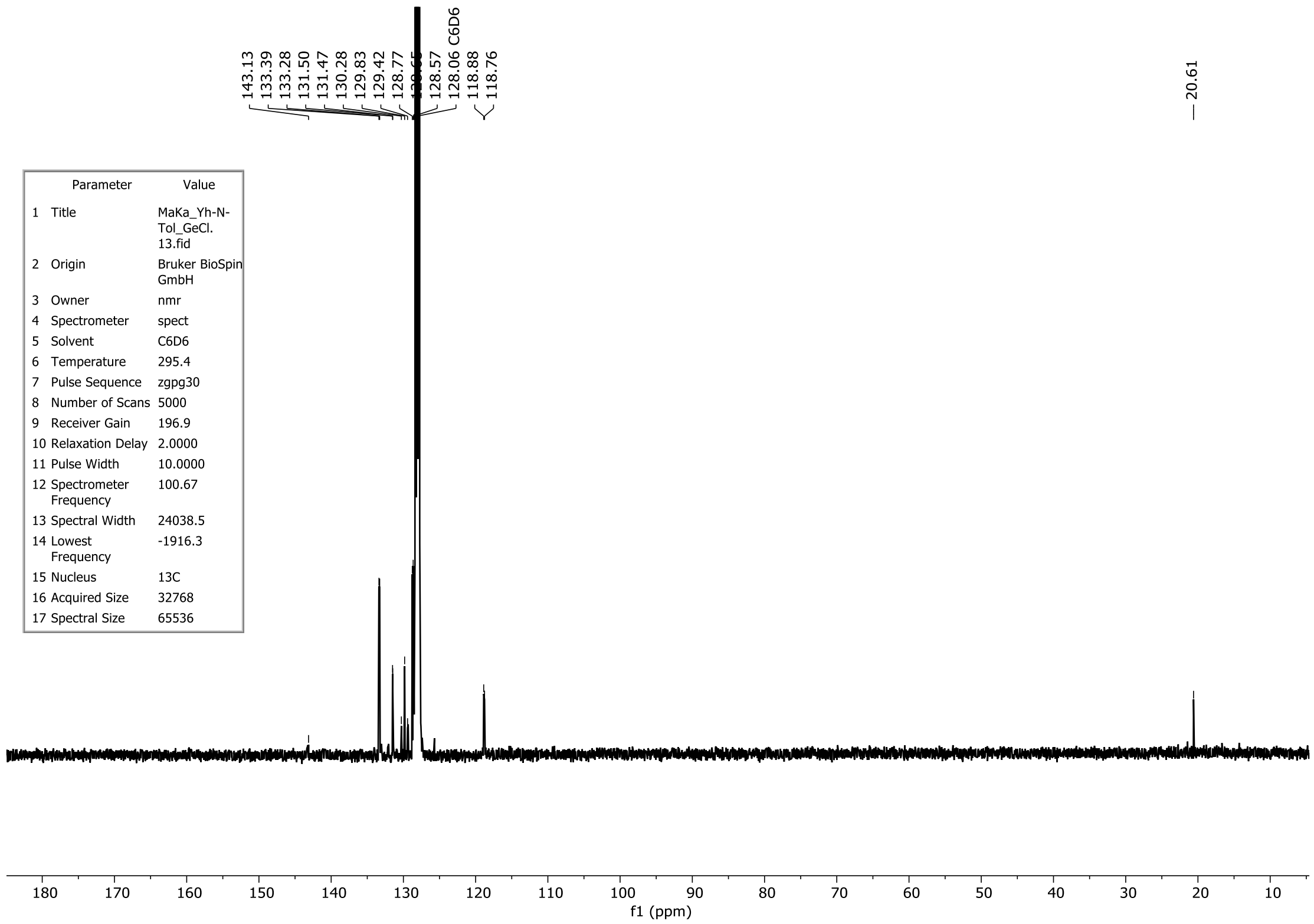

Figure S3. $^{13}C\{^{1}H\}$ NMR spectrum of compound **[$Y_{N\text{-}Tol}$–GeCl]** in Benzene-d_6.

2.2 NMR spectra of compound 2

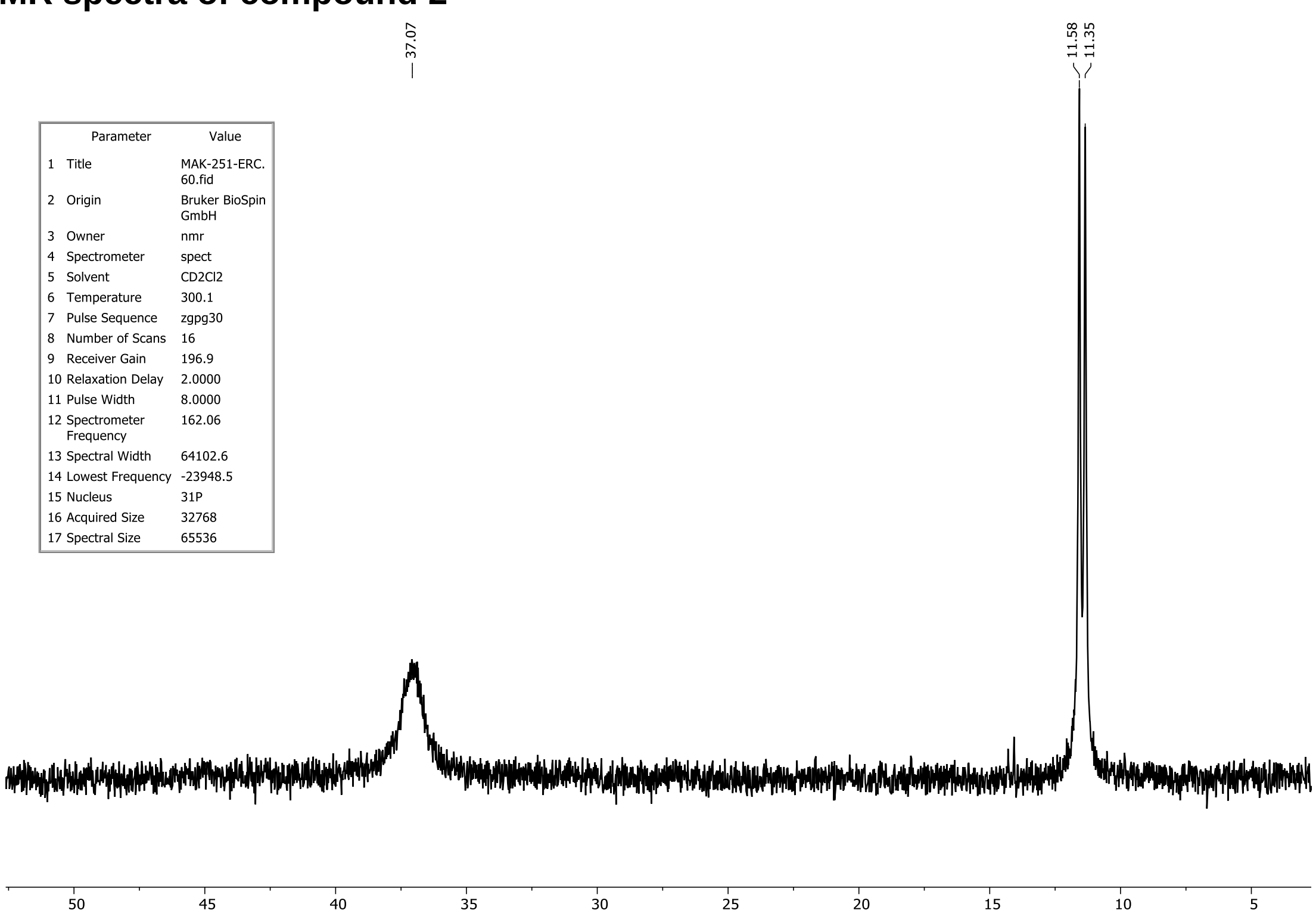

Figure S4. $^{31}P\{^{1}H\}$ NMR spectrum of compound **2** in DCM-d_2.

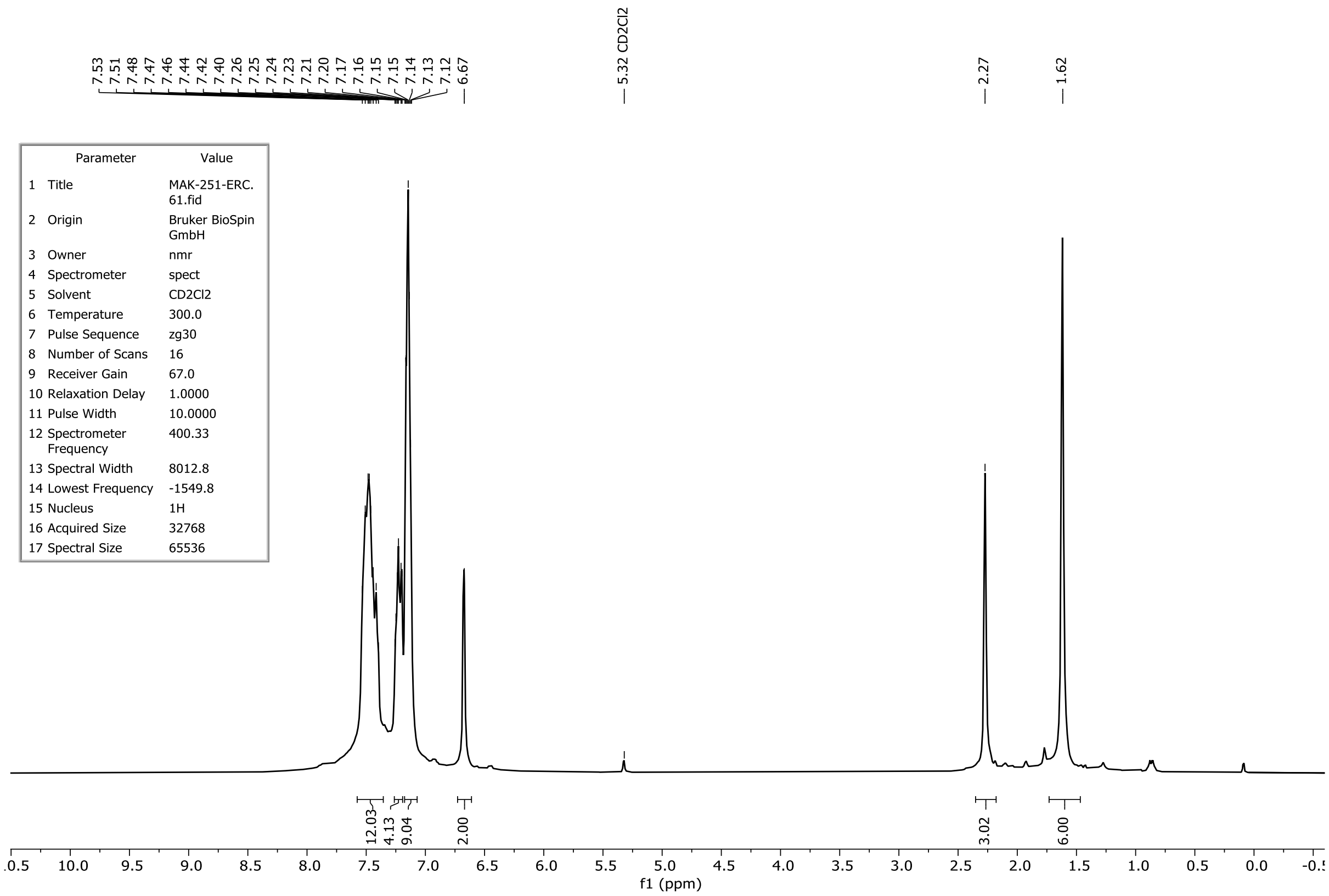

Figure S5.^{1}H NMR spectrum of compound **2** in DCM-d_2.

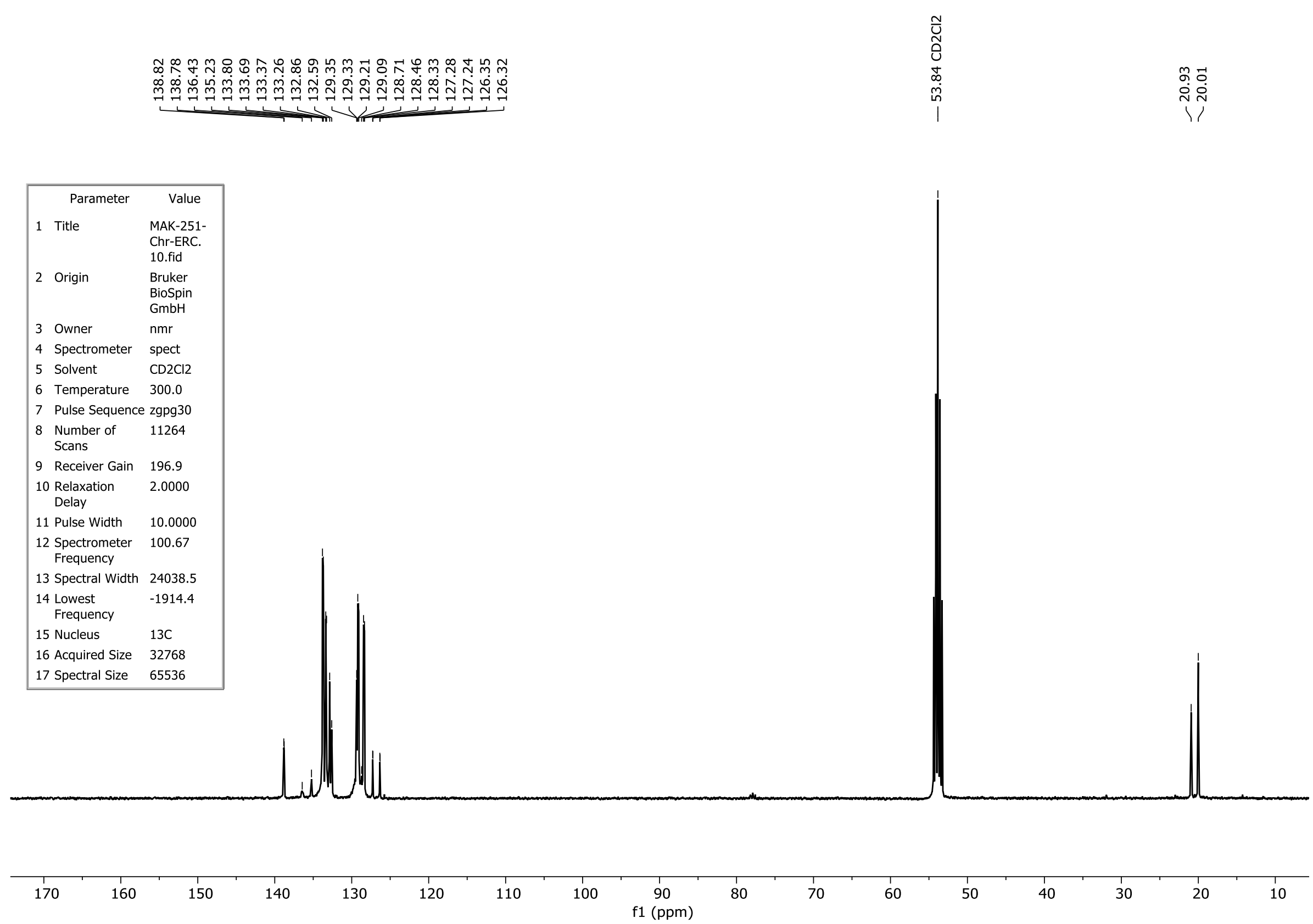

Figure S6. $^{13}C\{^1H\}$ NMR spectrum of compound **2** in DCM-d_2.

2.3 NMR spectra of compound 3

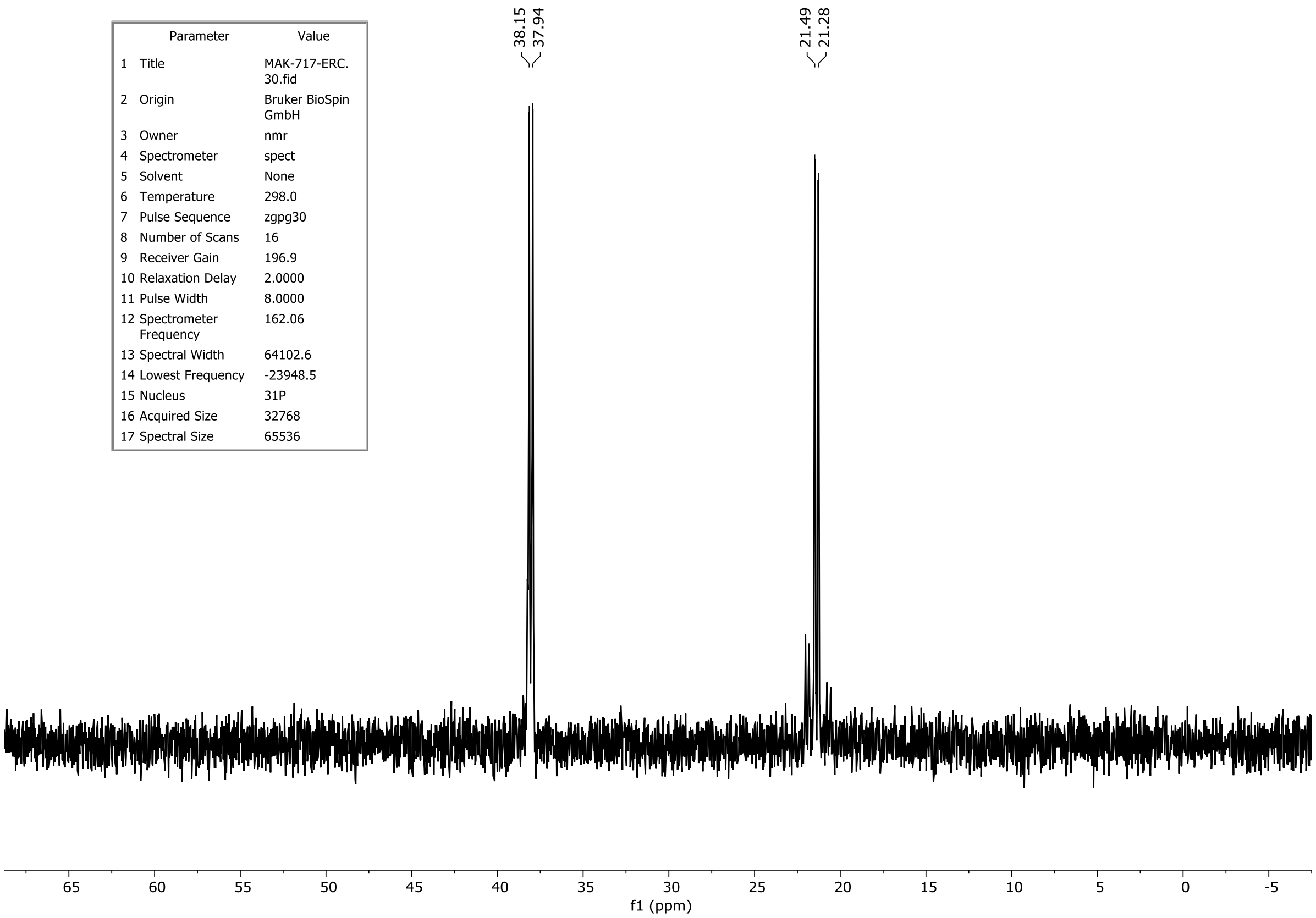

Figure S7. $^{31}P\{^1H\}$ NMR spectrum of compound **3** in DCM-d_2.

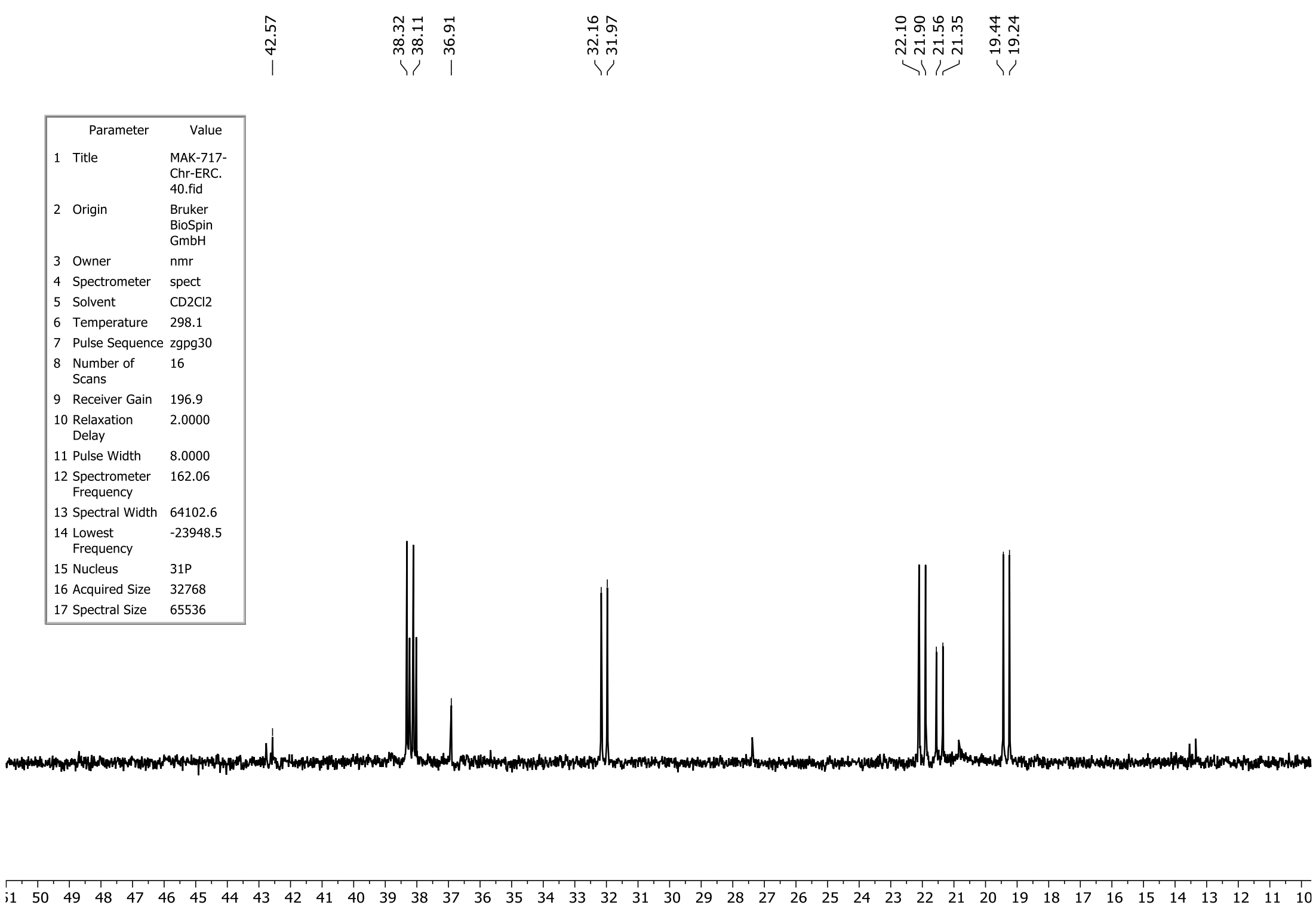

Figure S8. $^{31}P\{^1H\}$ NMR spectrum of compound **3** in DCM-d_2 after few hours.

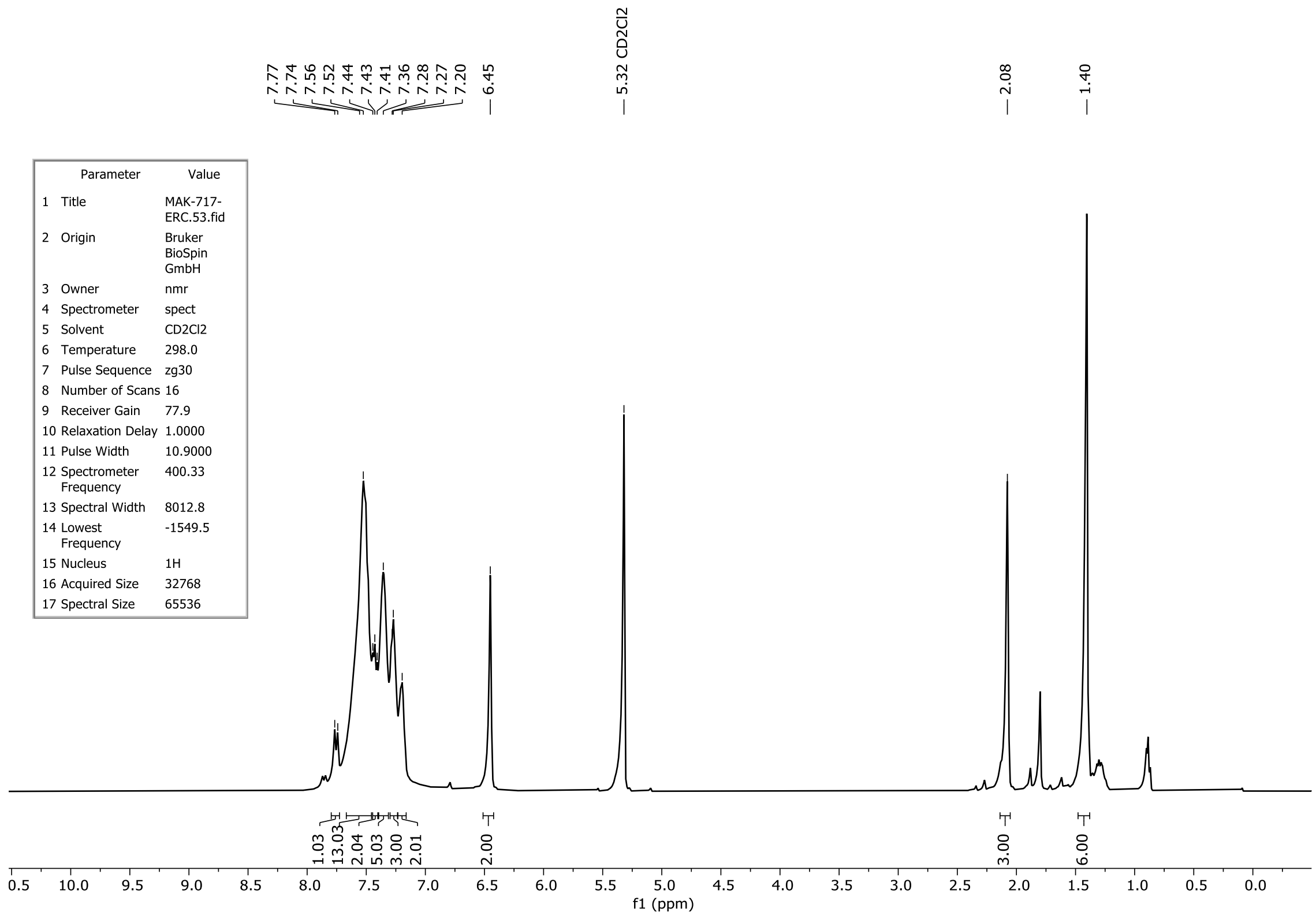

Figure S9. ^{1}H NMR spectrum of compound **3** in DCM-d_2.

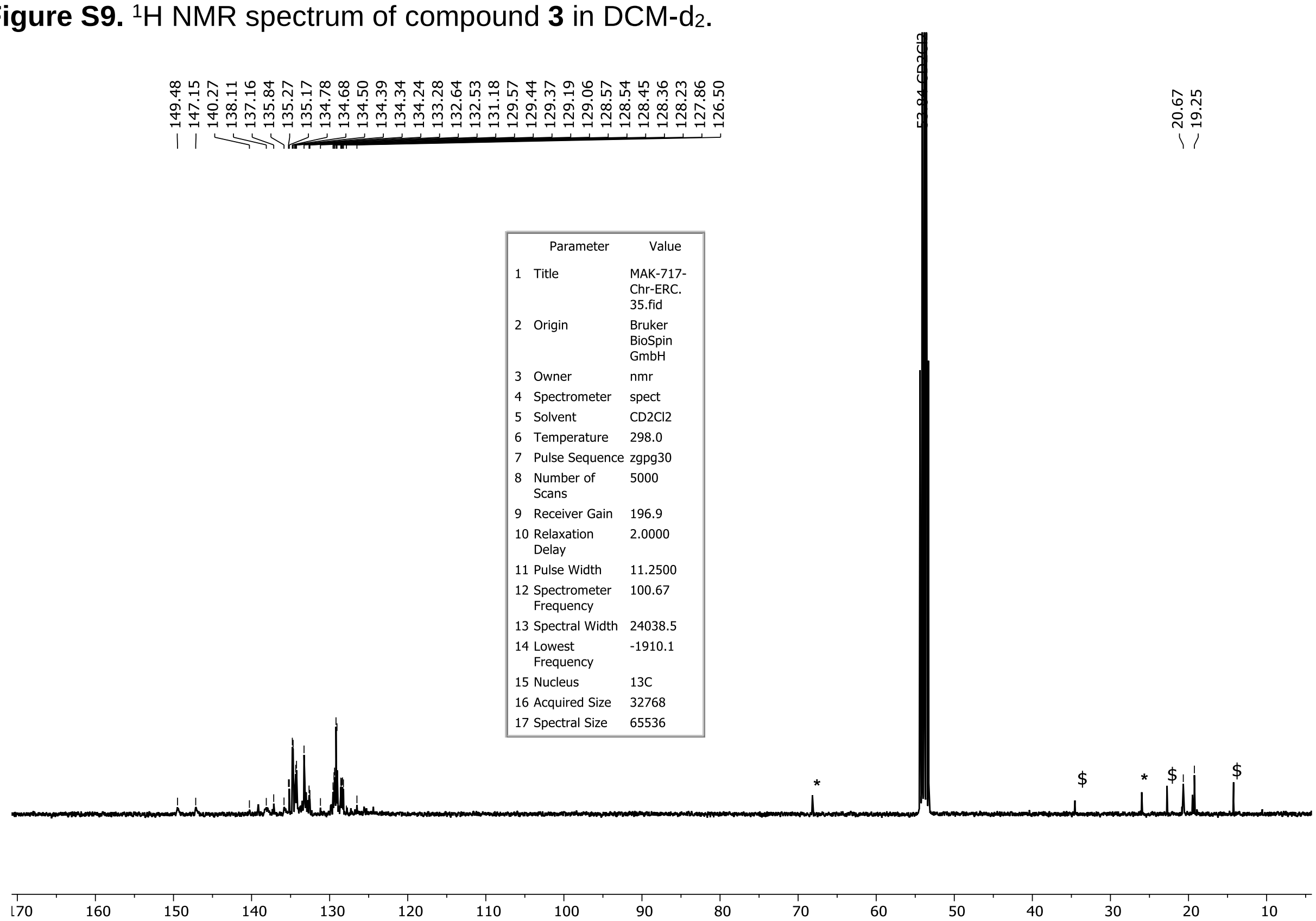

Figure S10. $^{13}C\{^{1}H\}$ NMR spectrum of compound **3** in DCM-d_2. The $^{13}C\{^{1}H\}$ NMR spectrum shows residues of thf and pentane (δ = 68.16 + 25.98 (*) and 34.57 + 22.77 + 14.24 ($) ppm).

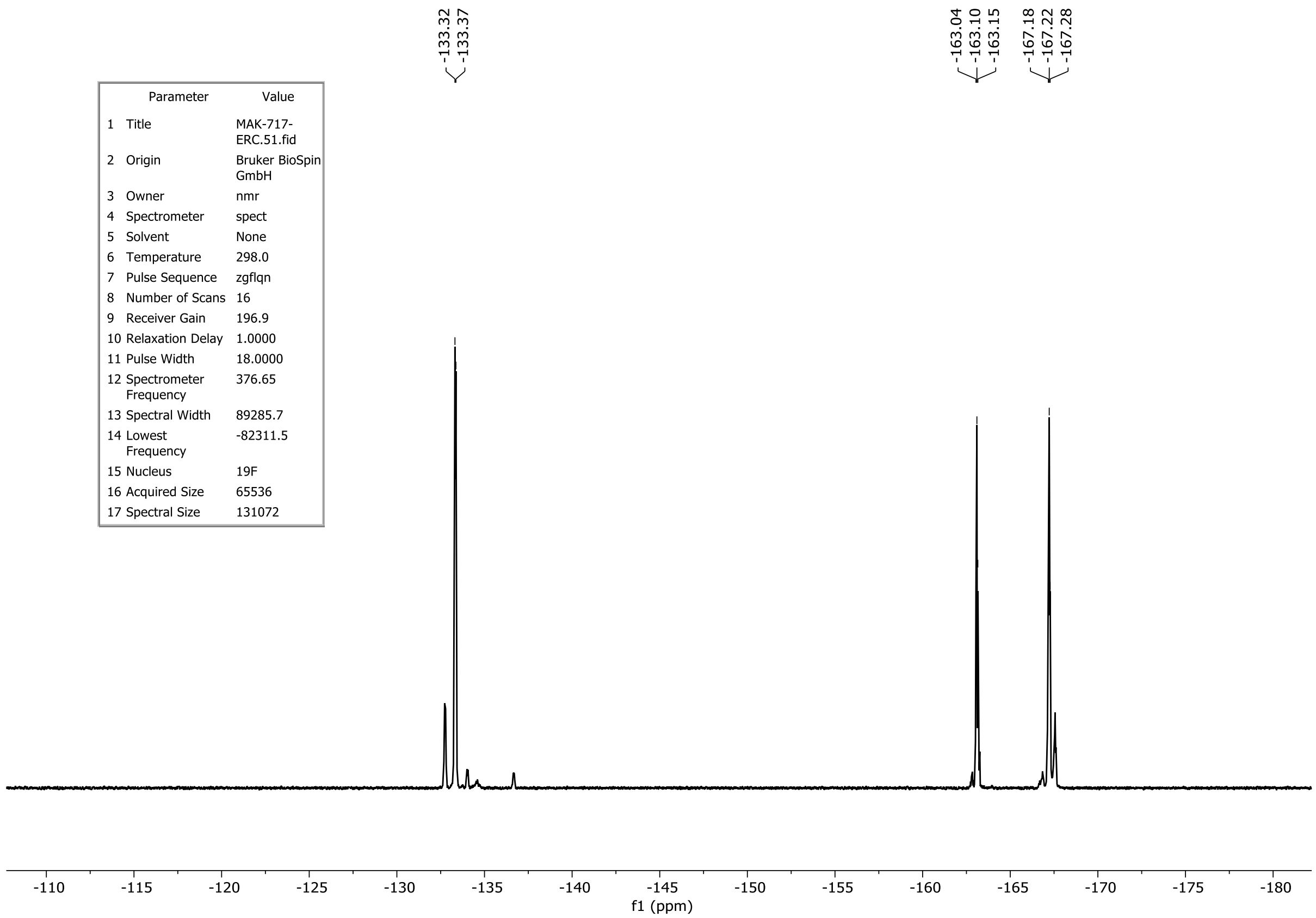

Figure S11. $^{19}F\{^{1}H\}$ NMR spectrum of compound **3** in DCM-d$_2$.

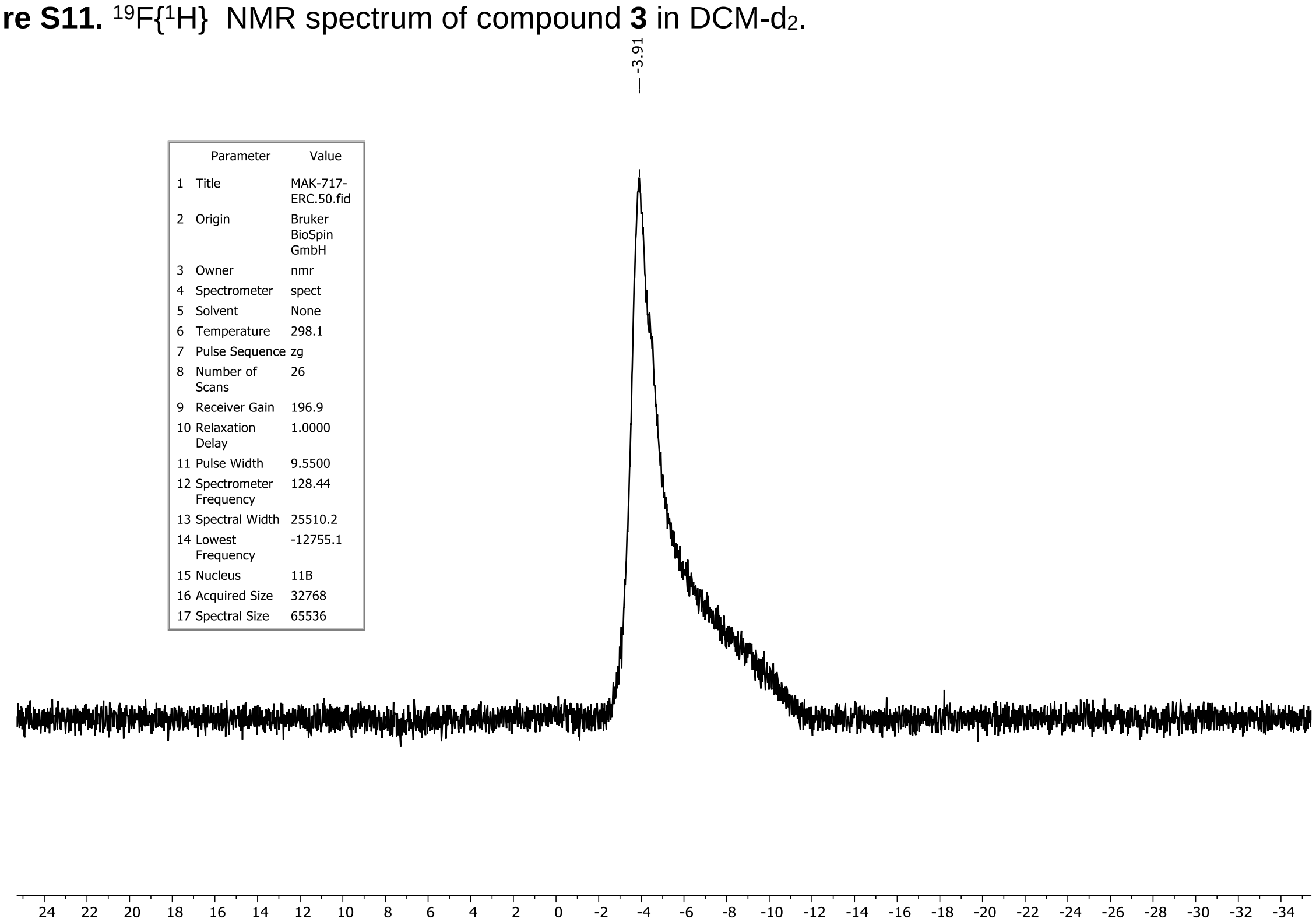

Figure S12. $^{11}B\{^{1}H\}$ NMR spectrum of compound **3** in DCM-d$_2$.

2.4 NMR spectra of compounds 4a–e

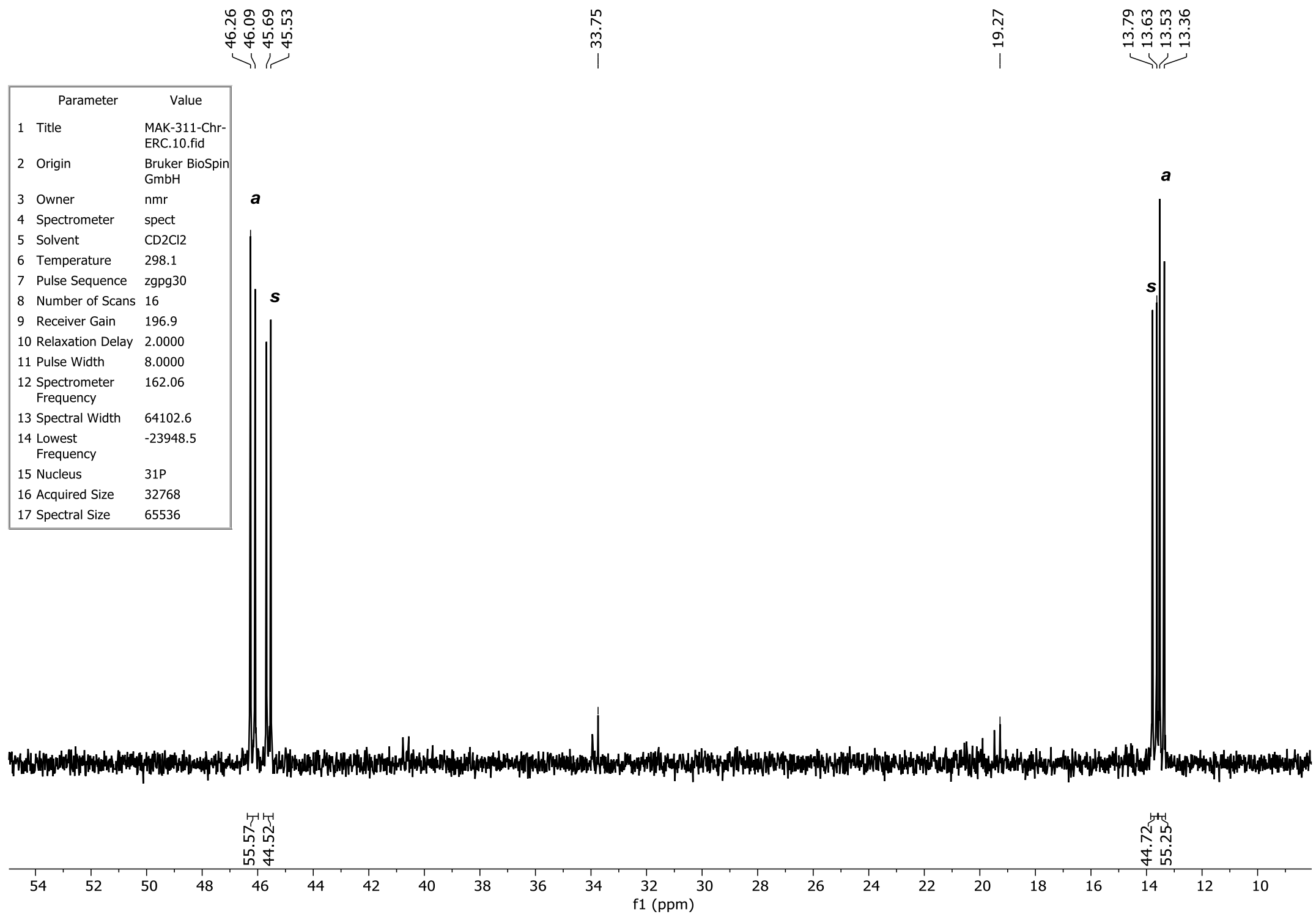

Figure S13. $^{31}P\{^1H\}$ NMR of compound **4a** in DCM-d$_2$. The ^{31}P NMR spectrum shows a small amount of decomposition during characterization of **4a** to its phosphonium salt at 33.74 (d, $^2J_{PP}$ = 31.85 Hz, ***P***Ph$_2$N) and 19.28 (d, $^2J_{PP}$ = 31.85 Hz, ***P***Ph$_3$).

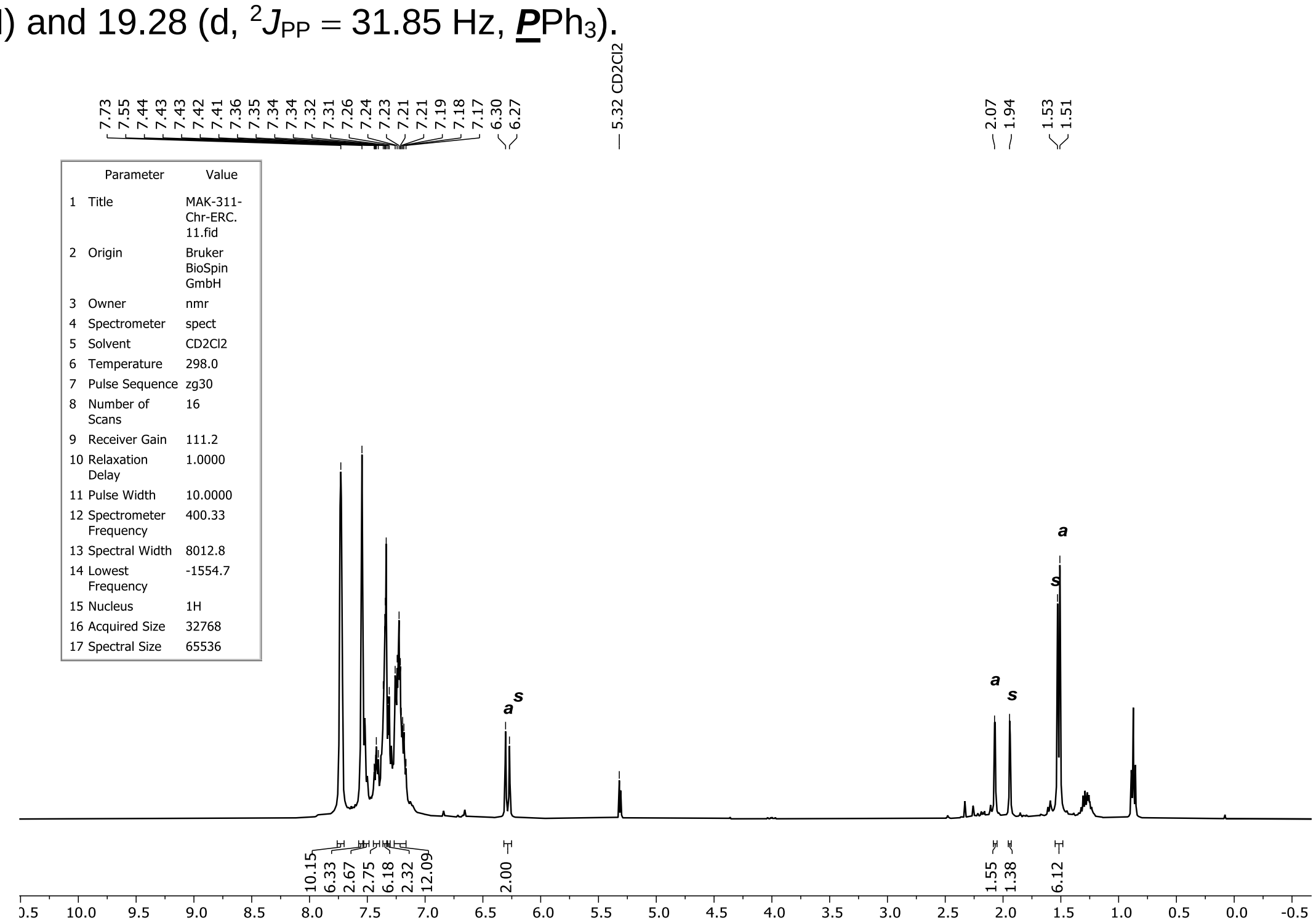

Figure S14. ^1H NMR of compound **4a** in DCM-d$_2$. The ^1H NMR spectrum shows overintegration of BAr^F protons due to the presence of its decomposition product, i.e. Phosphonium salt with BAr^F as a counter anion.

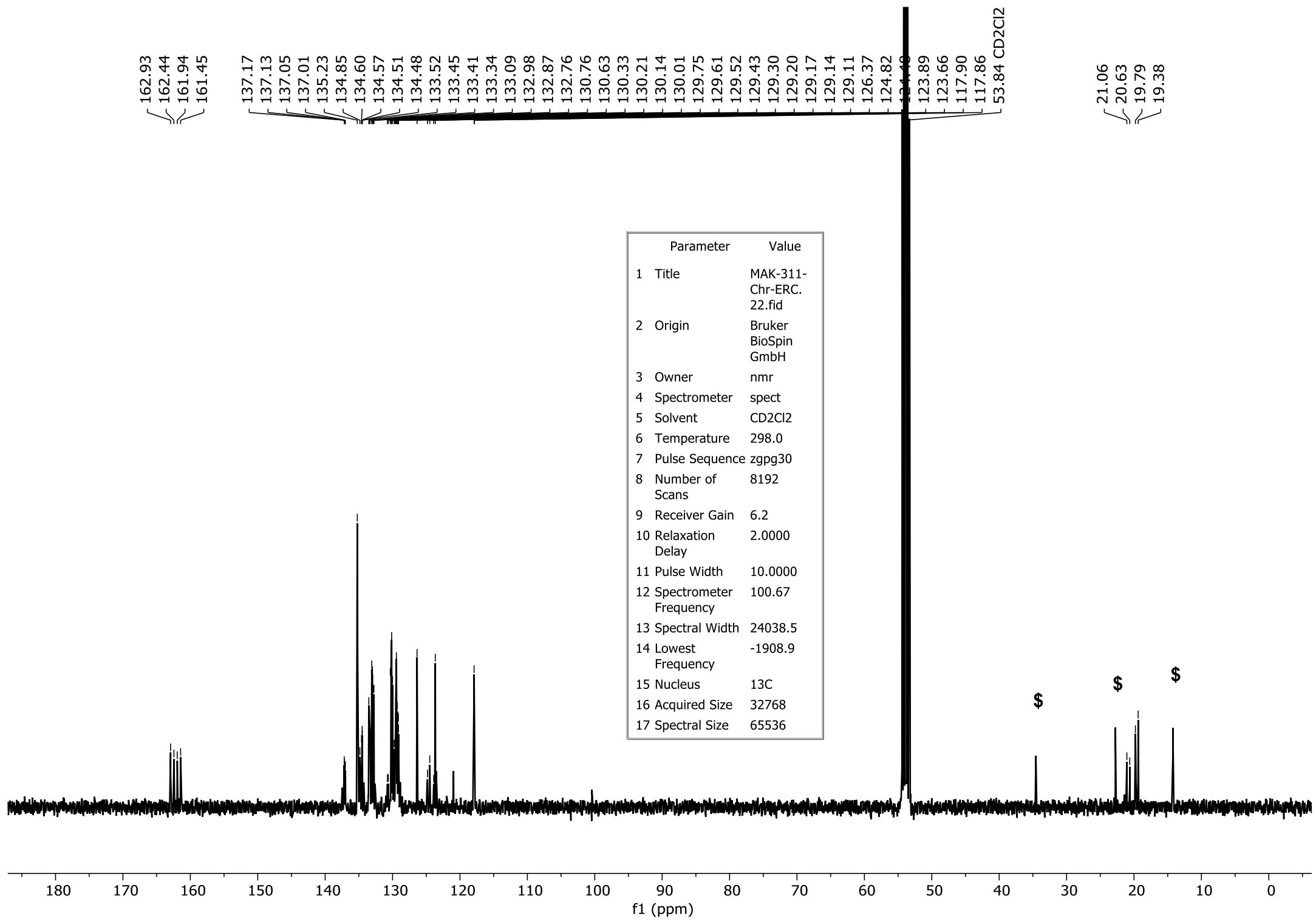

Figure S15. $^{13}C\{^1H\}$ NMR spectrum of compound **4a** in DCM-d_2. The $^{13}C\{^1H\}$ NMR spectrum shows residues of pentane (δ = 34.57 + 22.77 + 14.24 ($) ppm).

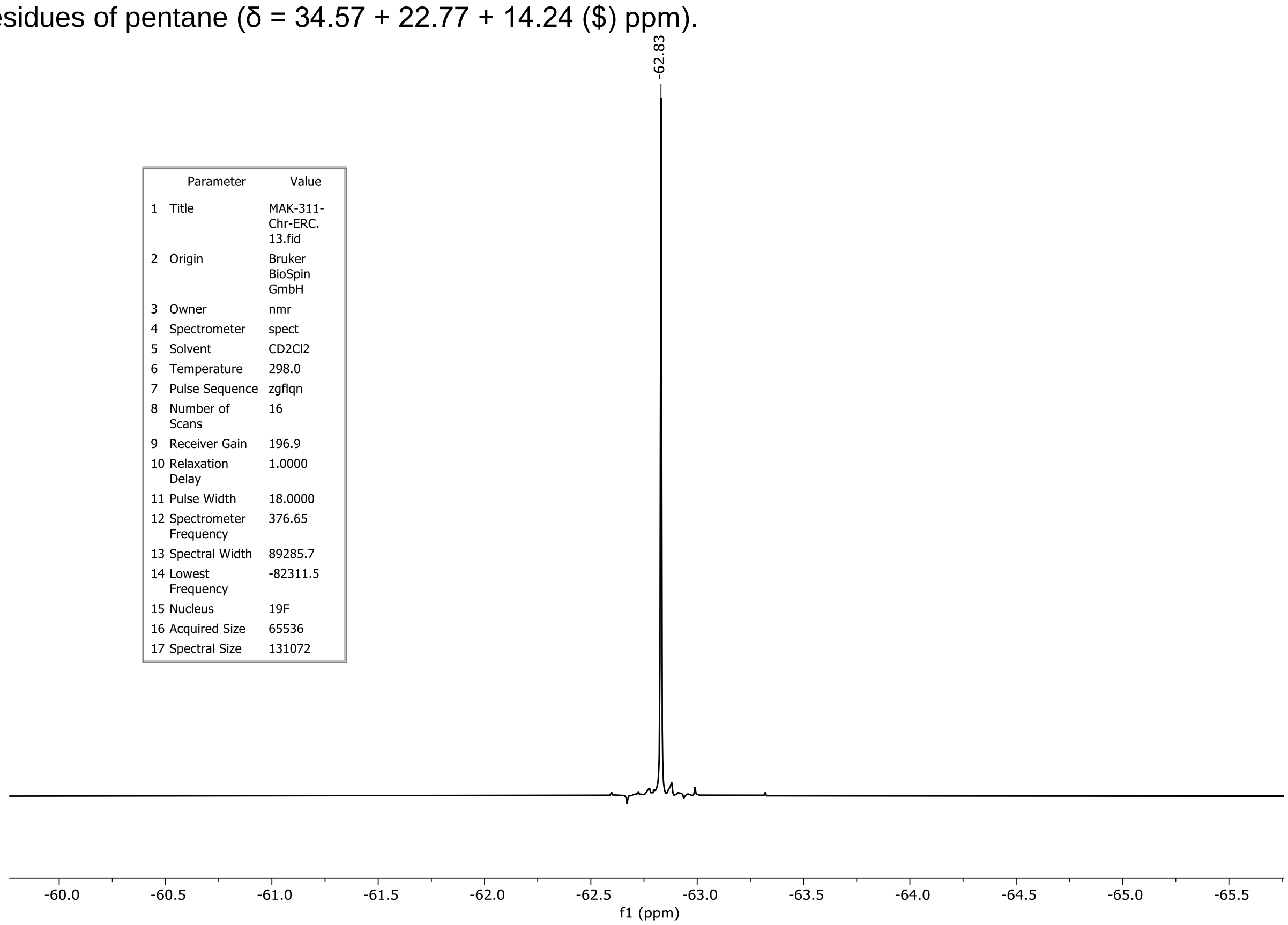

Figure S16. $^{19}F\{^1H\}$ NMR spectrum of compound **4a** in DCM-d_2.

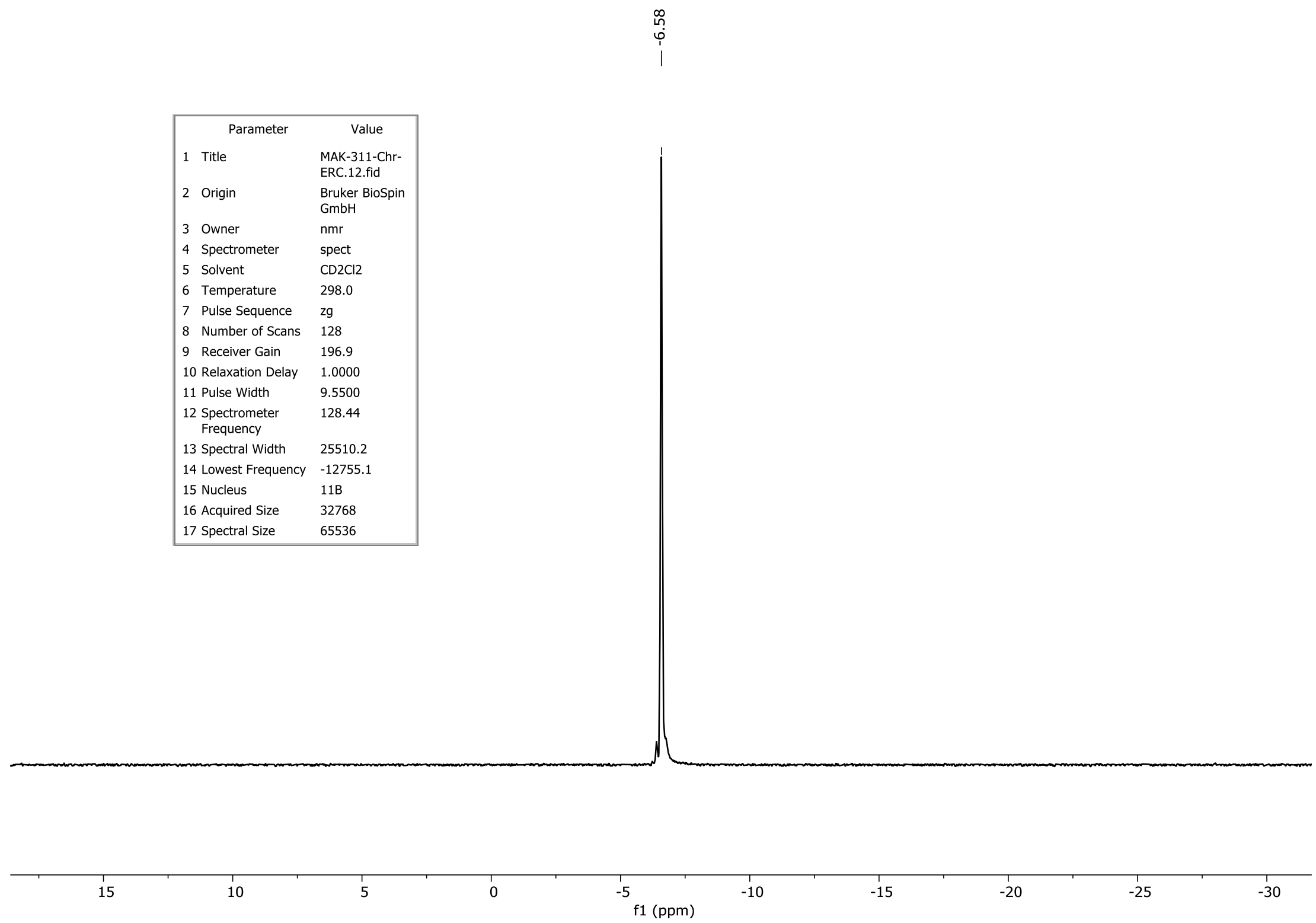

Figure S17. $^{11}B\{^{1}H\}$ NMR spectrum of compound **4a** in DCM-d_2.

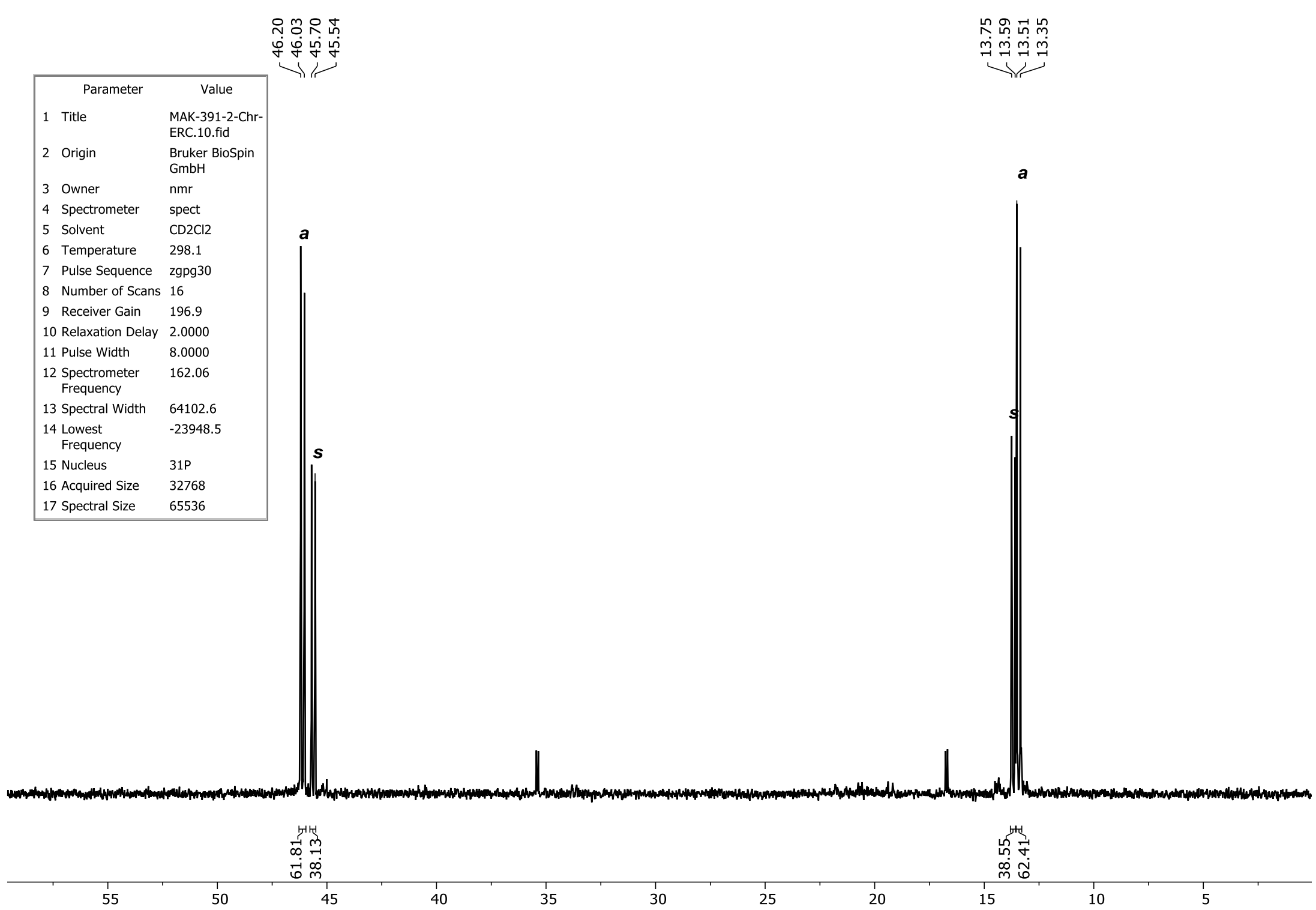

Figure S18. $^{31}P\{^{1}H\}$ NMR spectrum of compound **4b** in DCM-d_2. The ^{31}P NMR spectrum shows a small amount of decomposition during characterization of **4b** to its phosphonium salt at 33.74 (d, $^{2}J_{PP}$ = 31.85 Hz, ***P***Ph_2N) and 19.28 (d, $^{2}J_{PP}$ = 31.85 Hz, ***P***Ph_3).

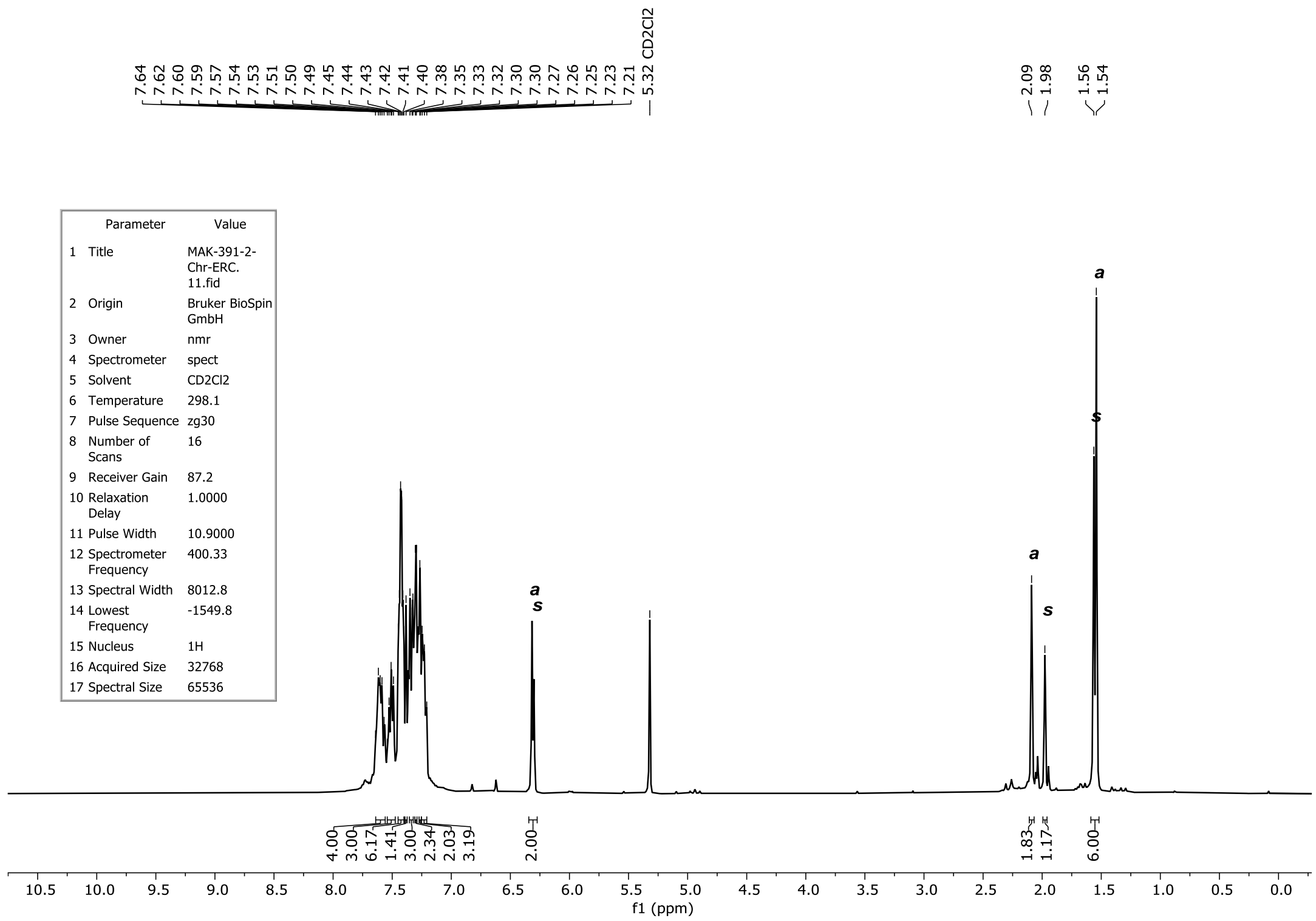

Figure S19. ^{1}H NMR spectrum of compound **4b** in DCM-d_2.

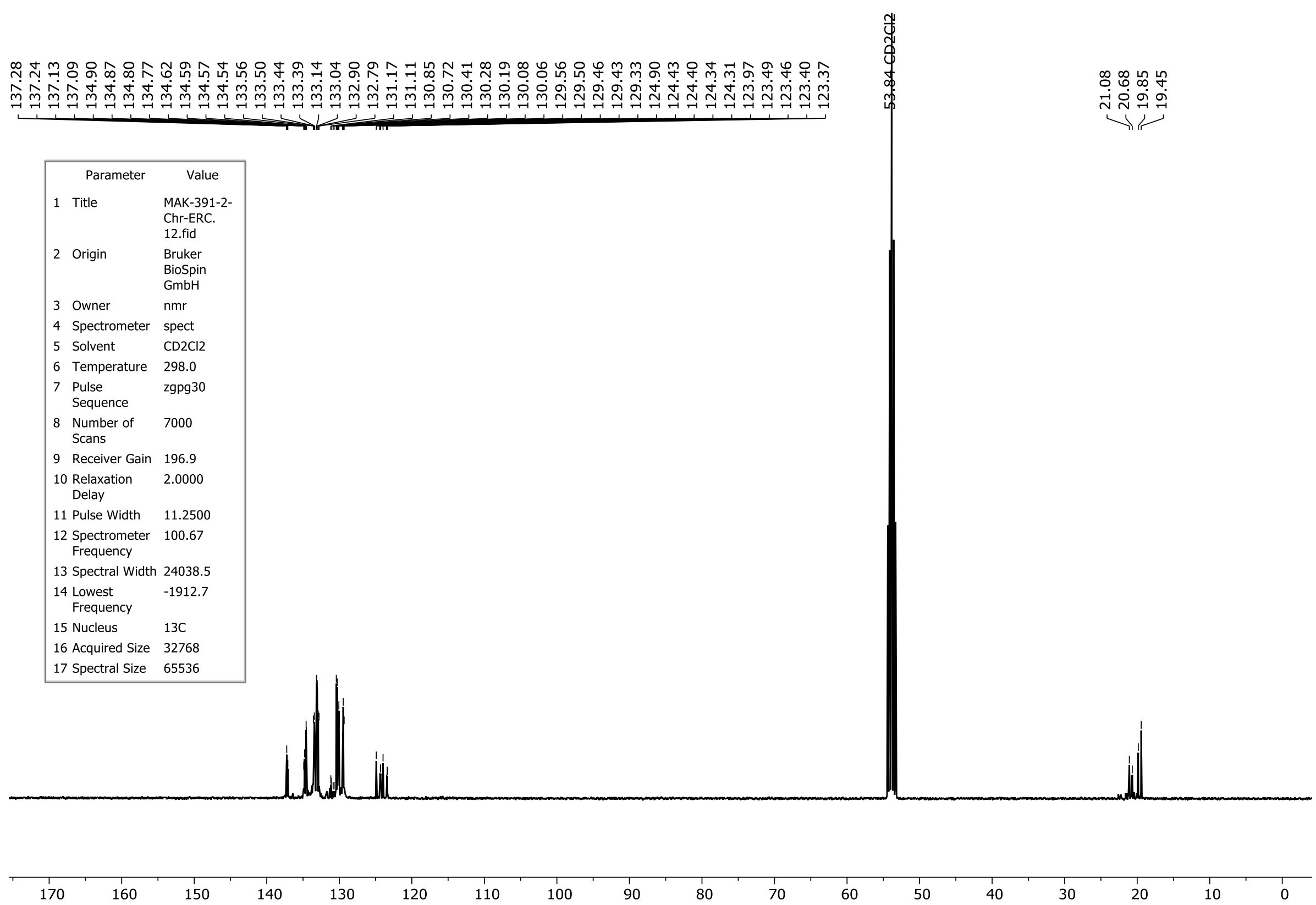

Figure S20. $^{13}C\{^{1}H\}$ NMR spectrum of compound **4b** in DCM-d_2.

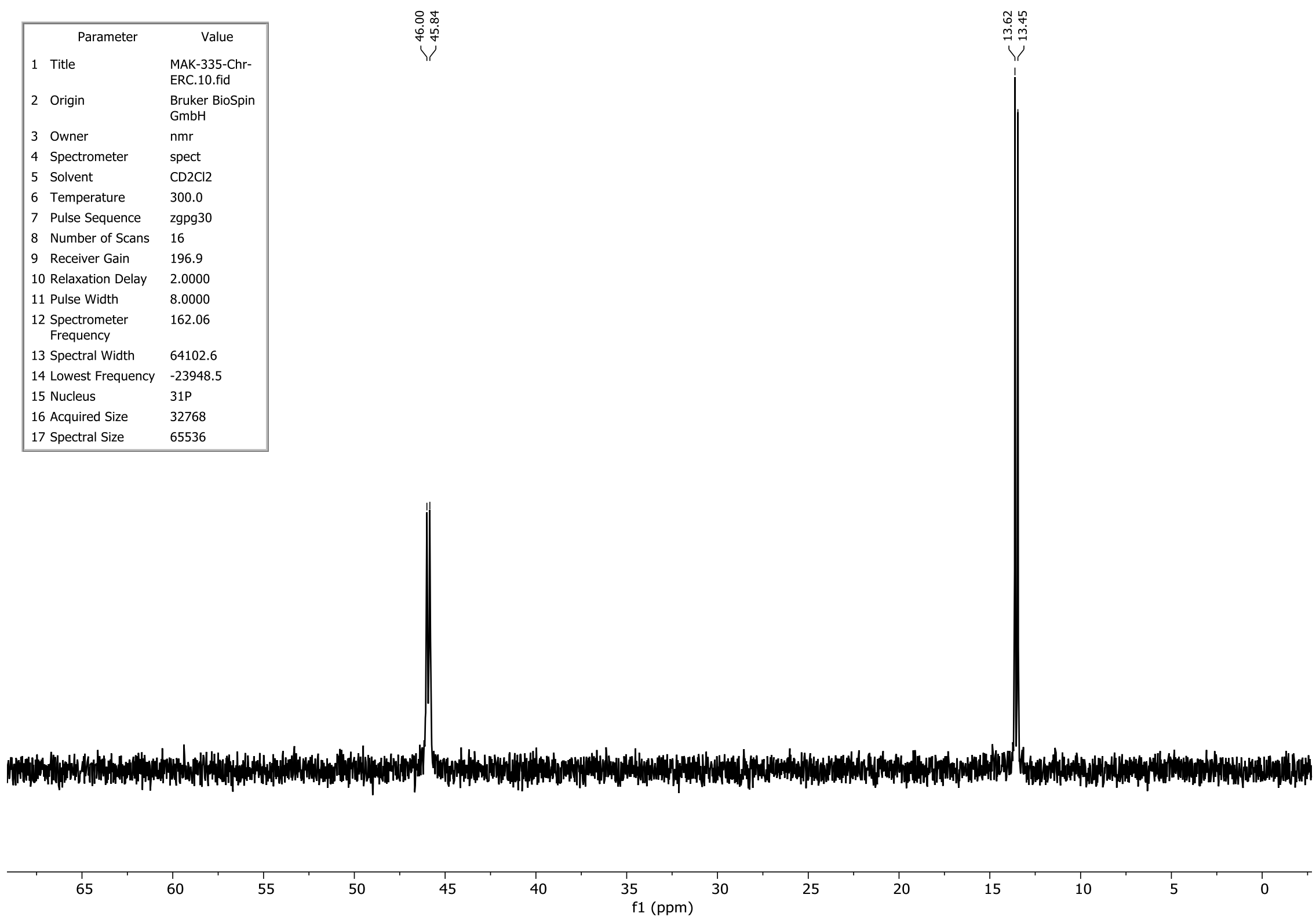

Figure S21. $^{31}P\{^1H\}$ NMR spectrum of compound **4c** in DCM-d_2.

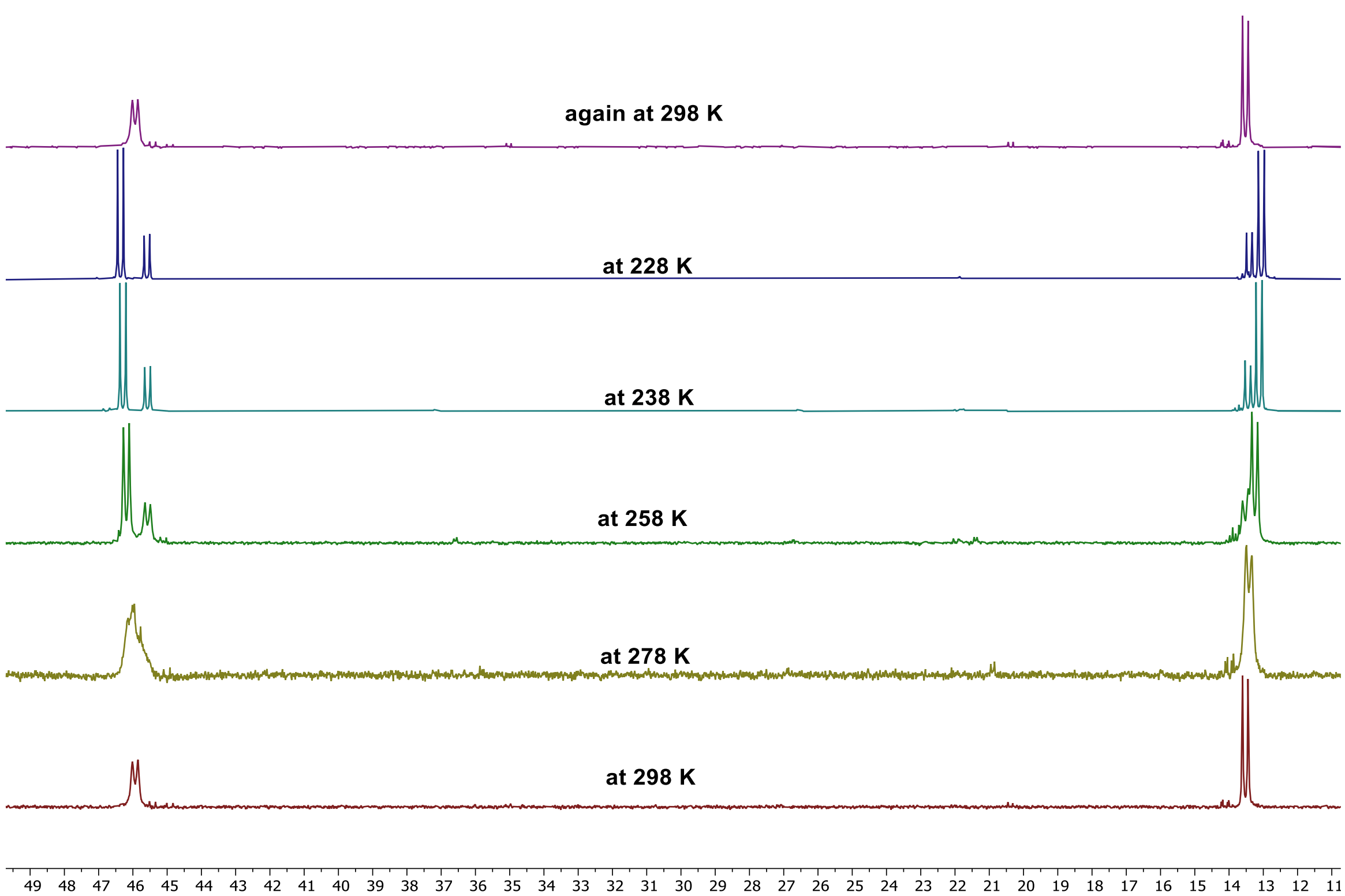

Figure S22. Variable-temperature (VT) $^{31}P\{^1H\}$ NMR spectrum of compound **4c** in DCM-d_2 from 298 to 228K.

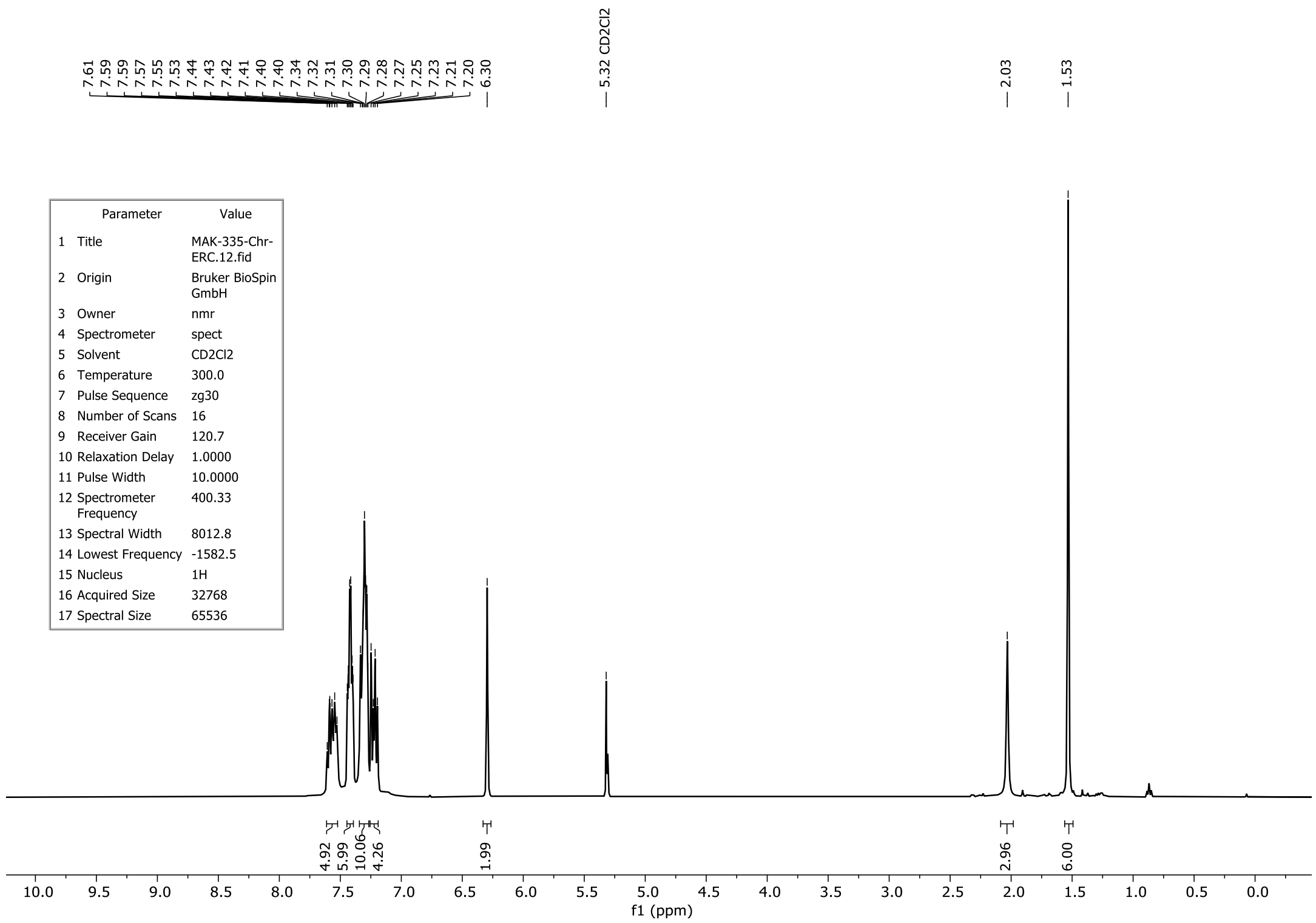

Figure S23.^{1}H NMR spectrum of compound **4c** in DCM-d_2.

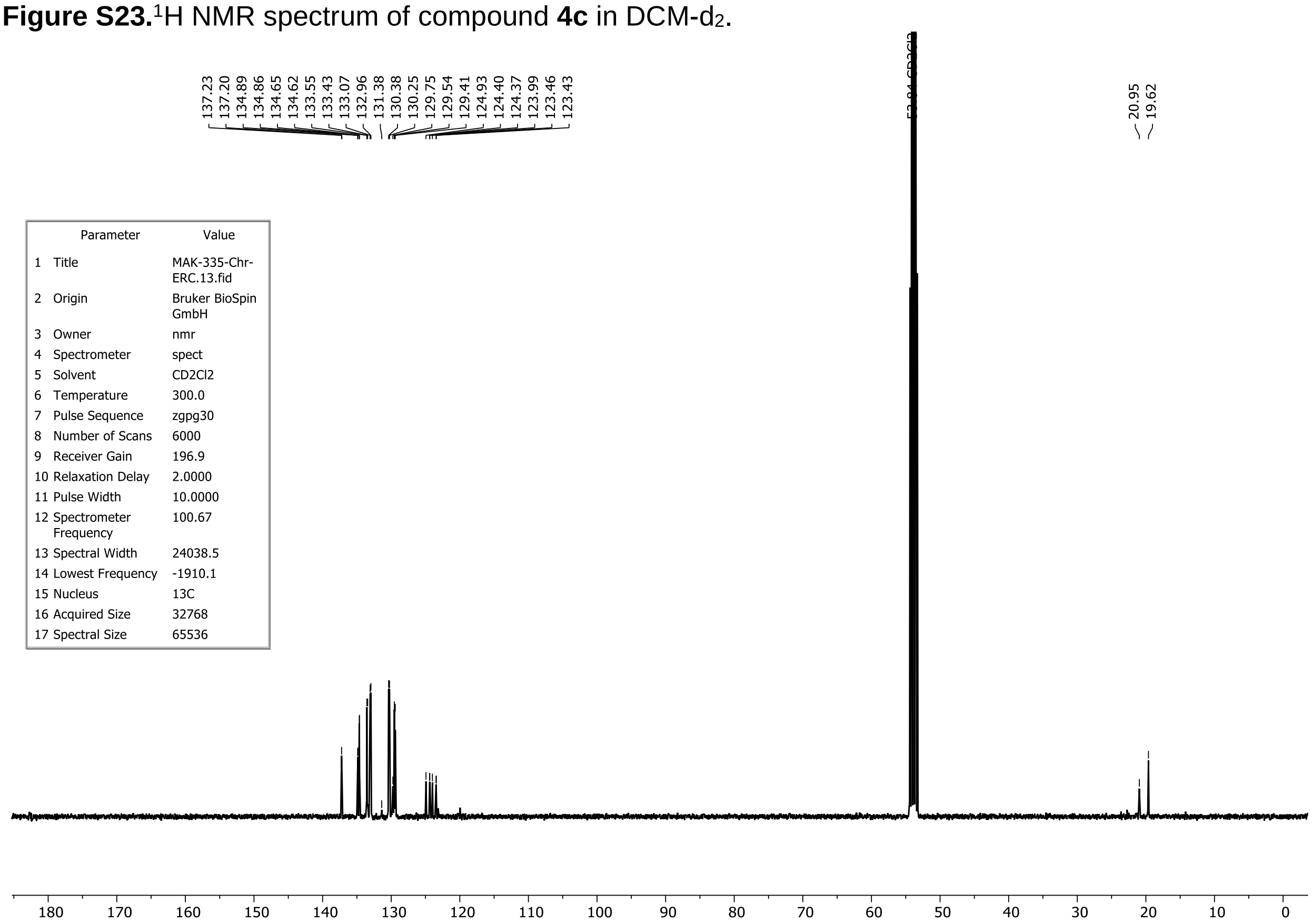

Figure S24.^{13}C{^{1}H} NMR spectrum of compound **4c** in DCM-d_2.

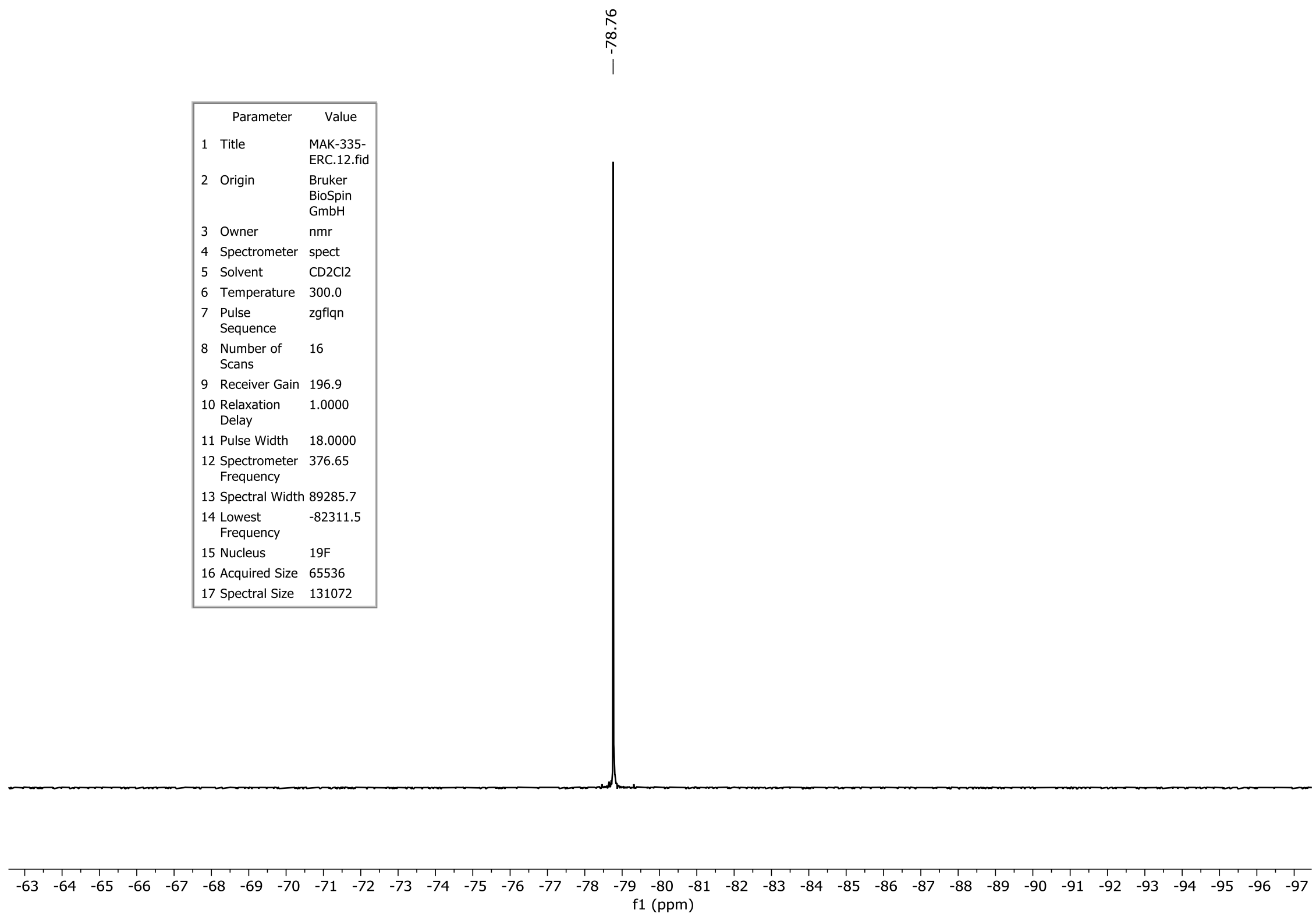

Figure S25. $^{19}F\{^{1}H\}$ NMR spectrum of compound **4c** in DCM-d_2.

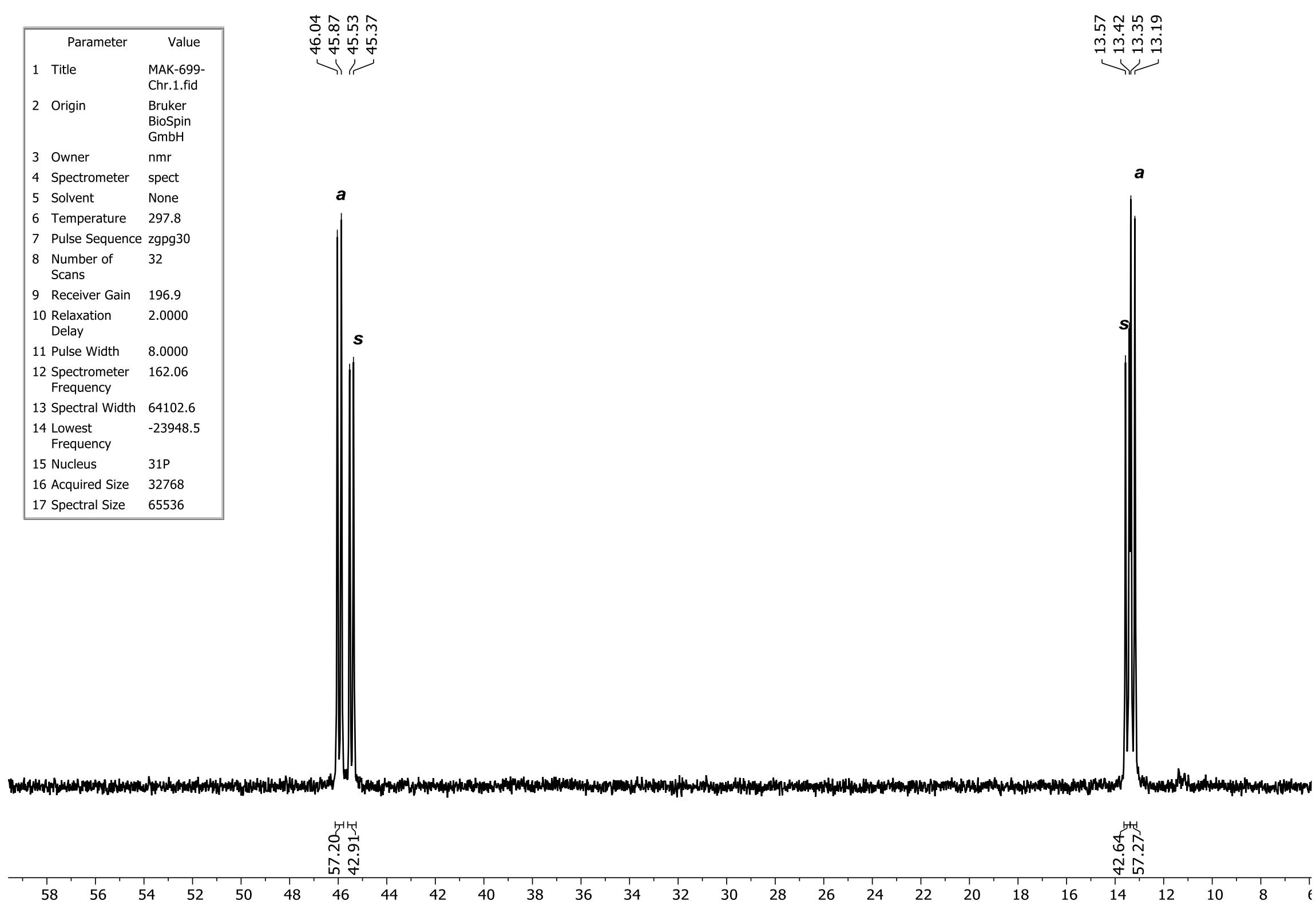

Figure S26. $^{31}P\{^{1}H\}$ NMR of compound **4d** in DCM-d_2.

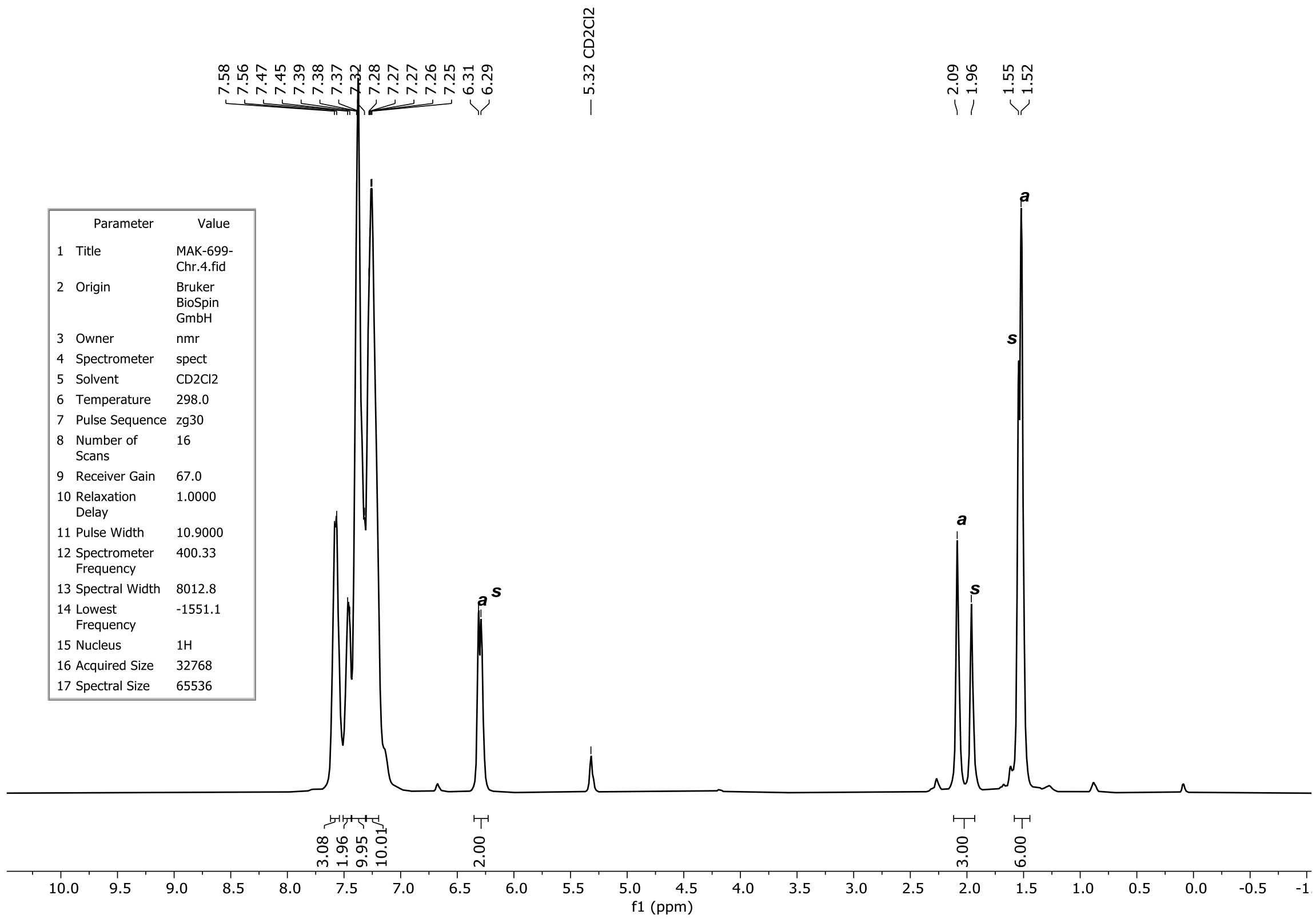

Figure S27. ^{1}H NMR spectrum of compound **4d** in DCM-d_2.

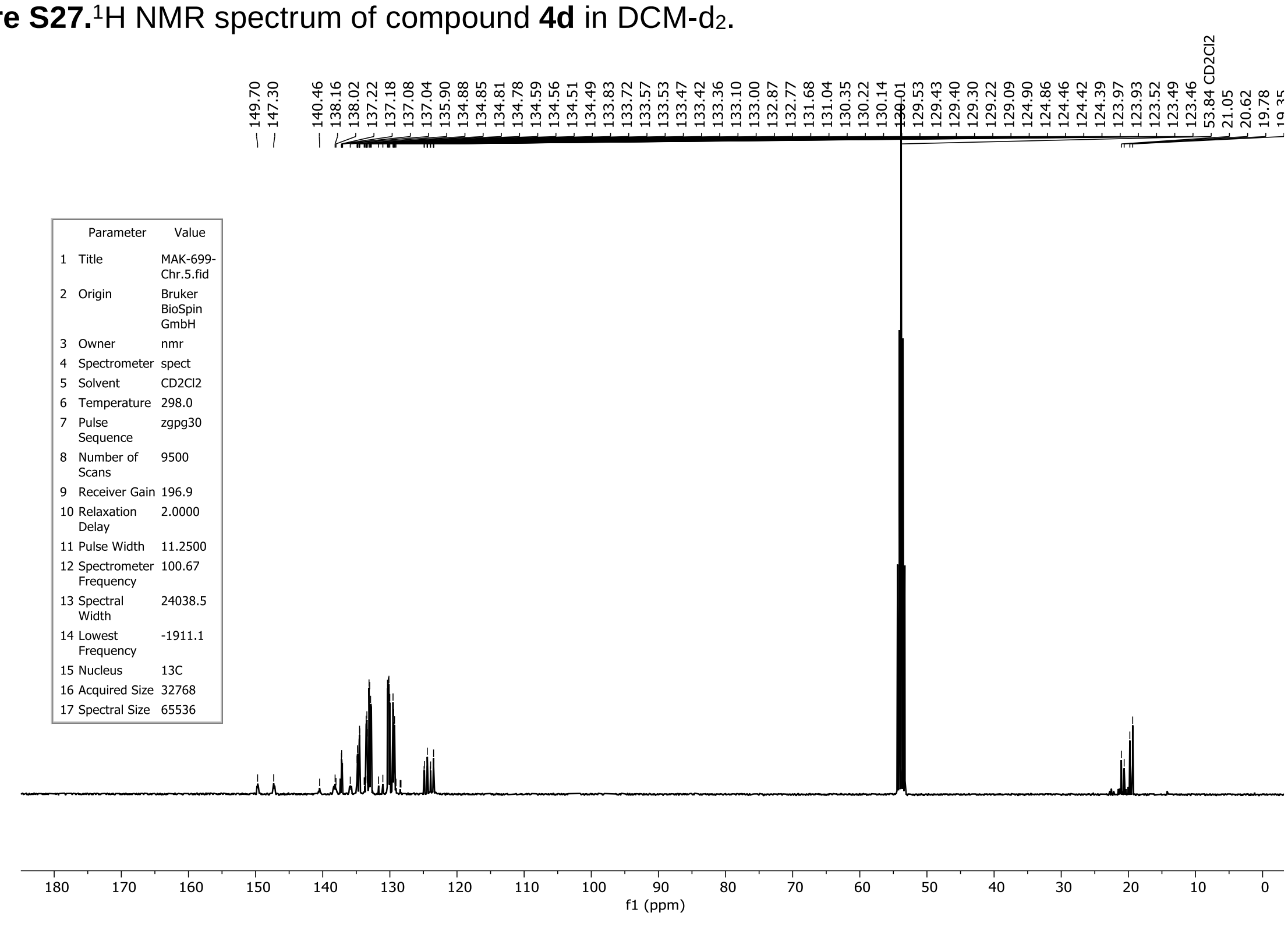

Figure S28. $^{13}C\{^{1}H\}$ NMR spectrum of compound **4d** in DCM-d_2.

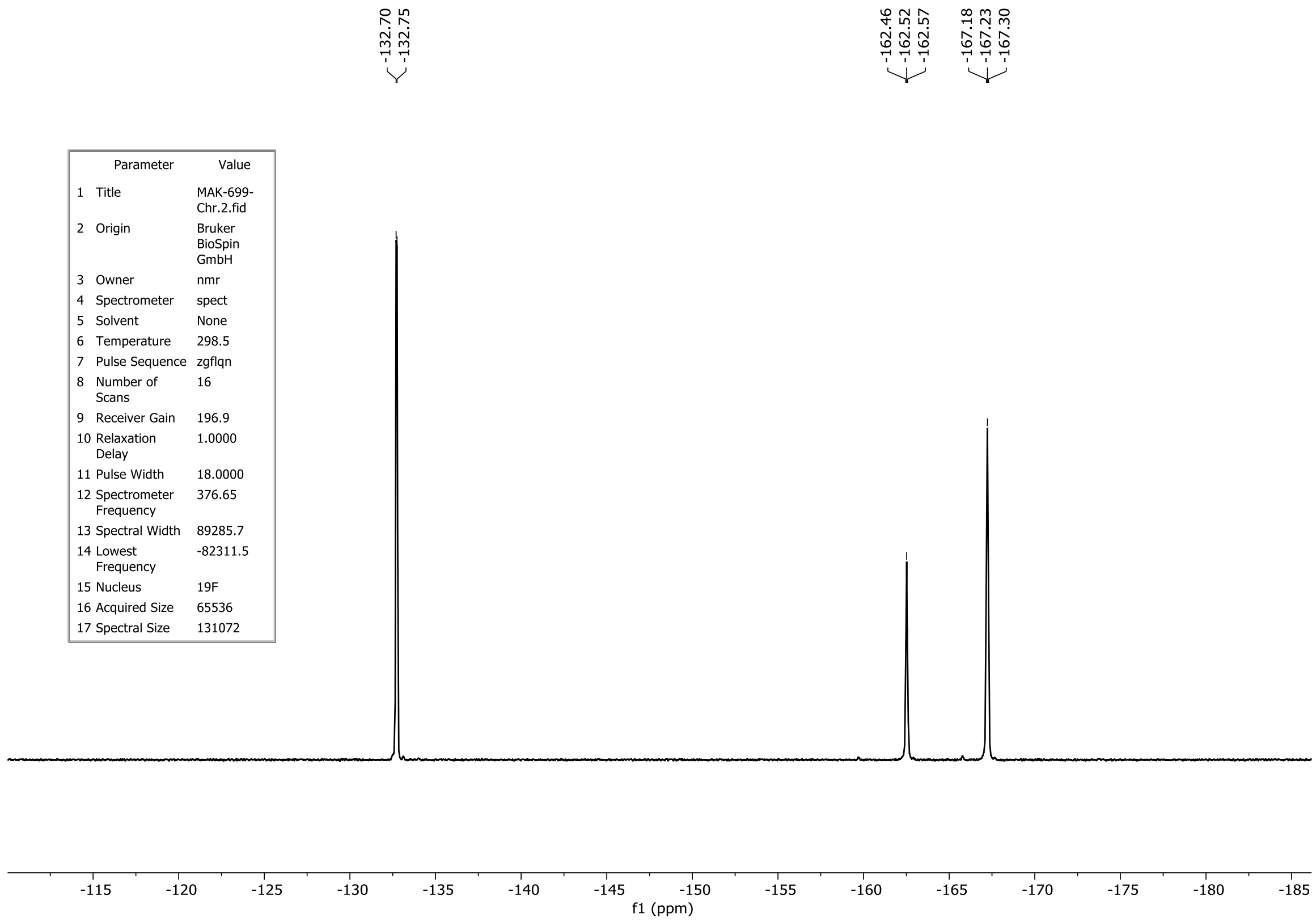

Figure S29. $^{19}F\{^{1}H\}$ NMR spectrum of compound **4d** in DCM-d_2.

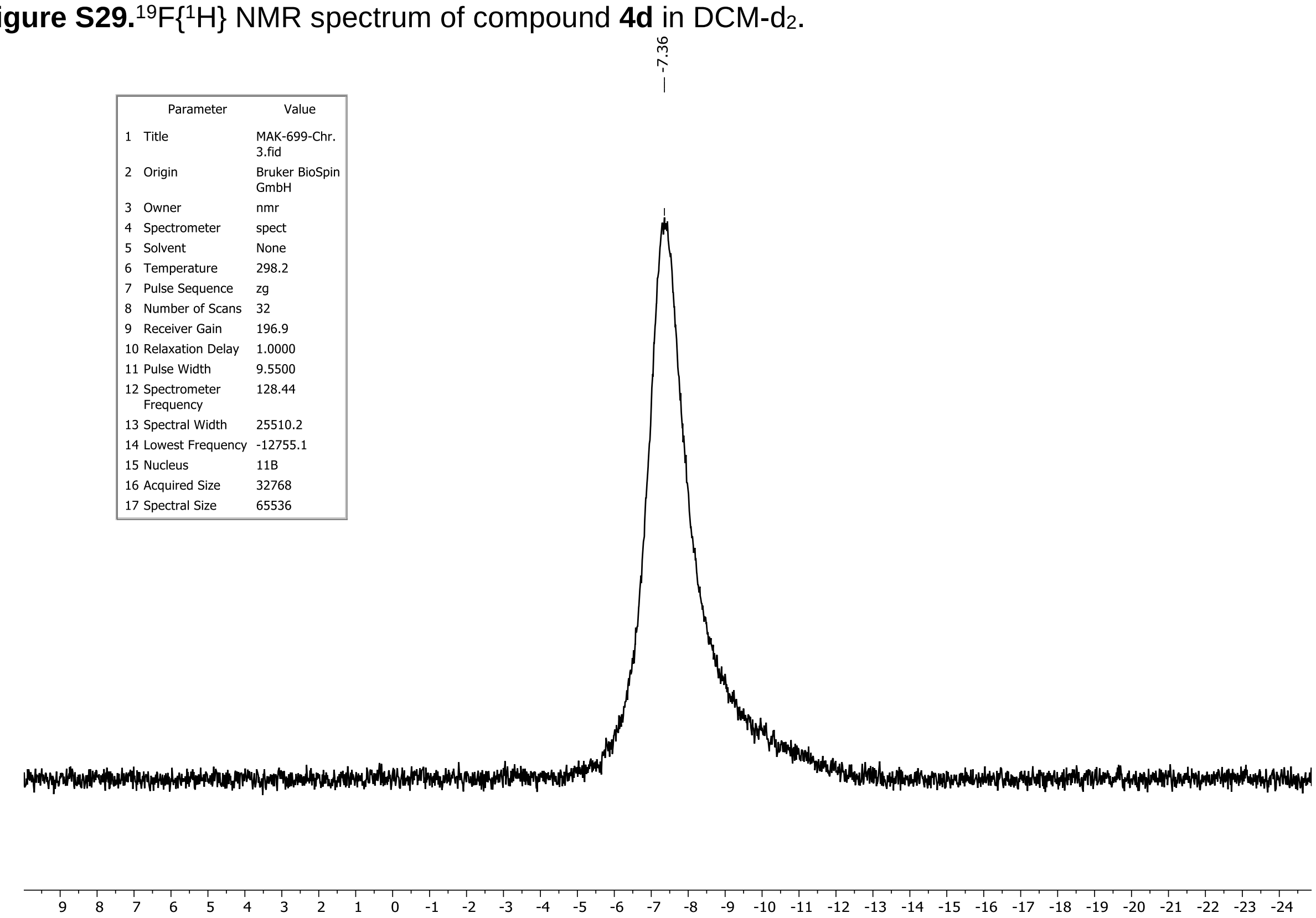

Figure S30. $^{11}B\{^{1}H\}$ NMR spectrum of compound **4d** in DCM-d_2.

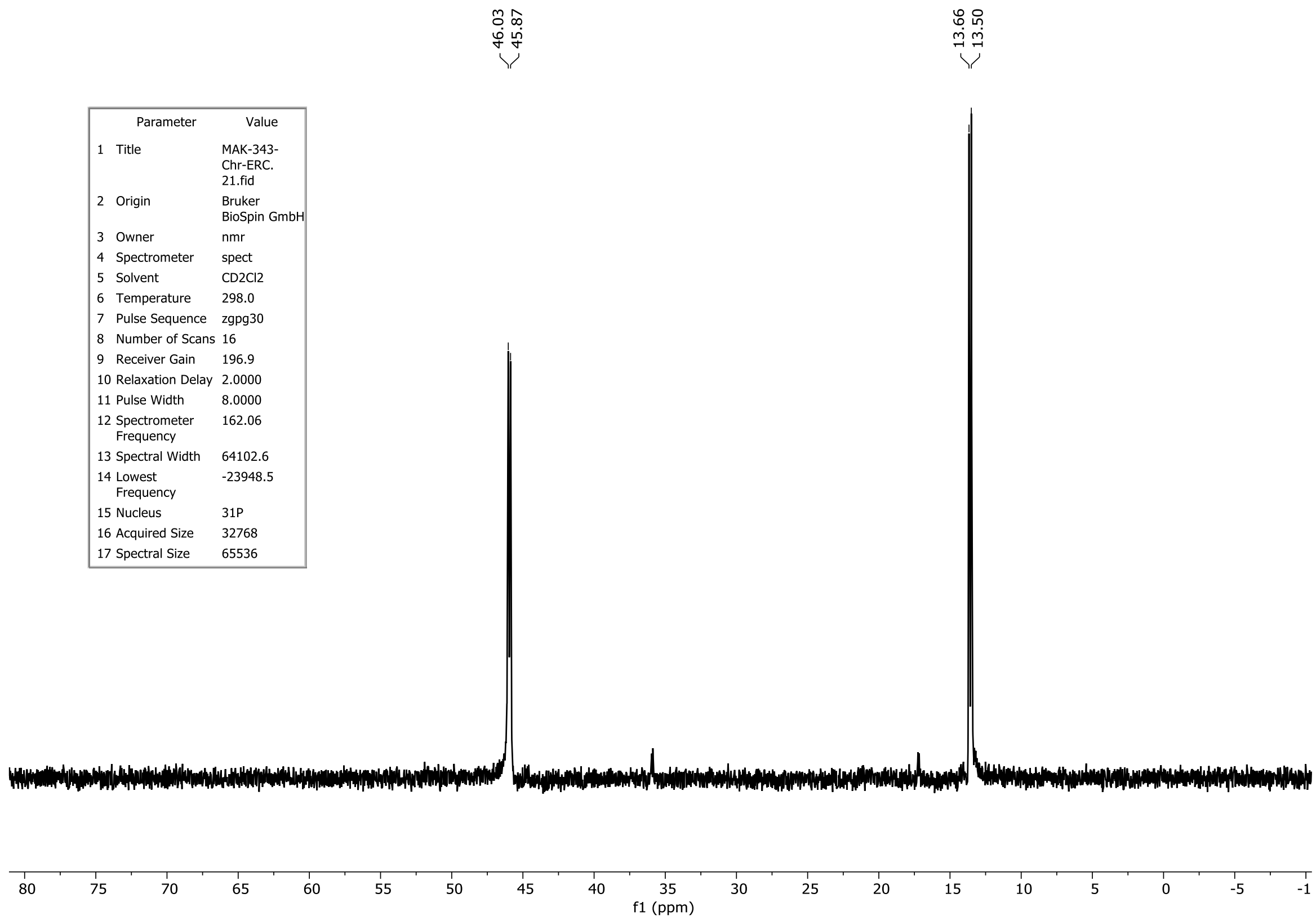

Figure S31. $^{31}P\{^1H\}$ NMR of compound **4e** in DCM-d_2. The ^{31}P NMR spectrum shows a small amount of decomposition during characterization of **4e** to its phosphonium salt at 33.74 (d, $^2J_{PP}$ = 31.85 Hz, ***P***Ph_2N) and 19.28 (d, $^2J_{PP}$ = 31.85 Hz, ***P***Ph_3).

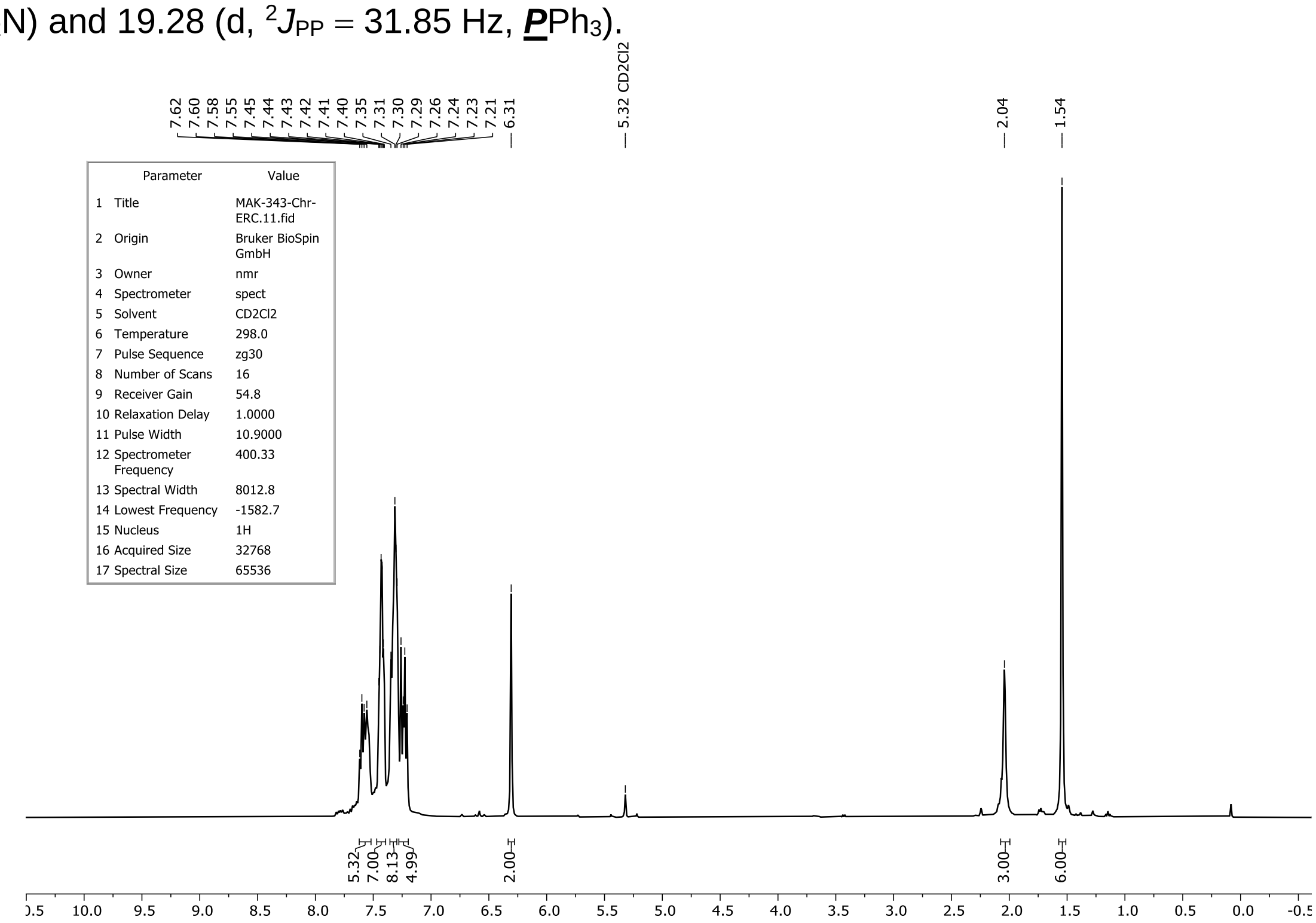

Figure S32. ^1H NMR of compound **4e** in DCM-d_2.

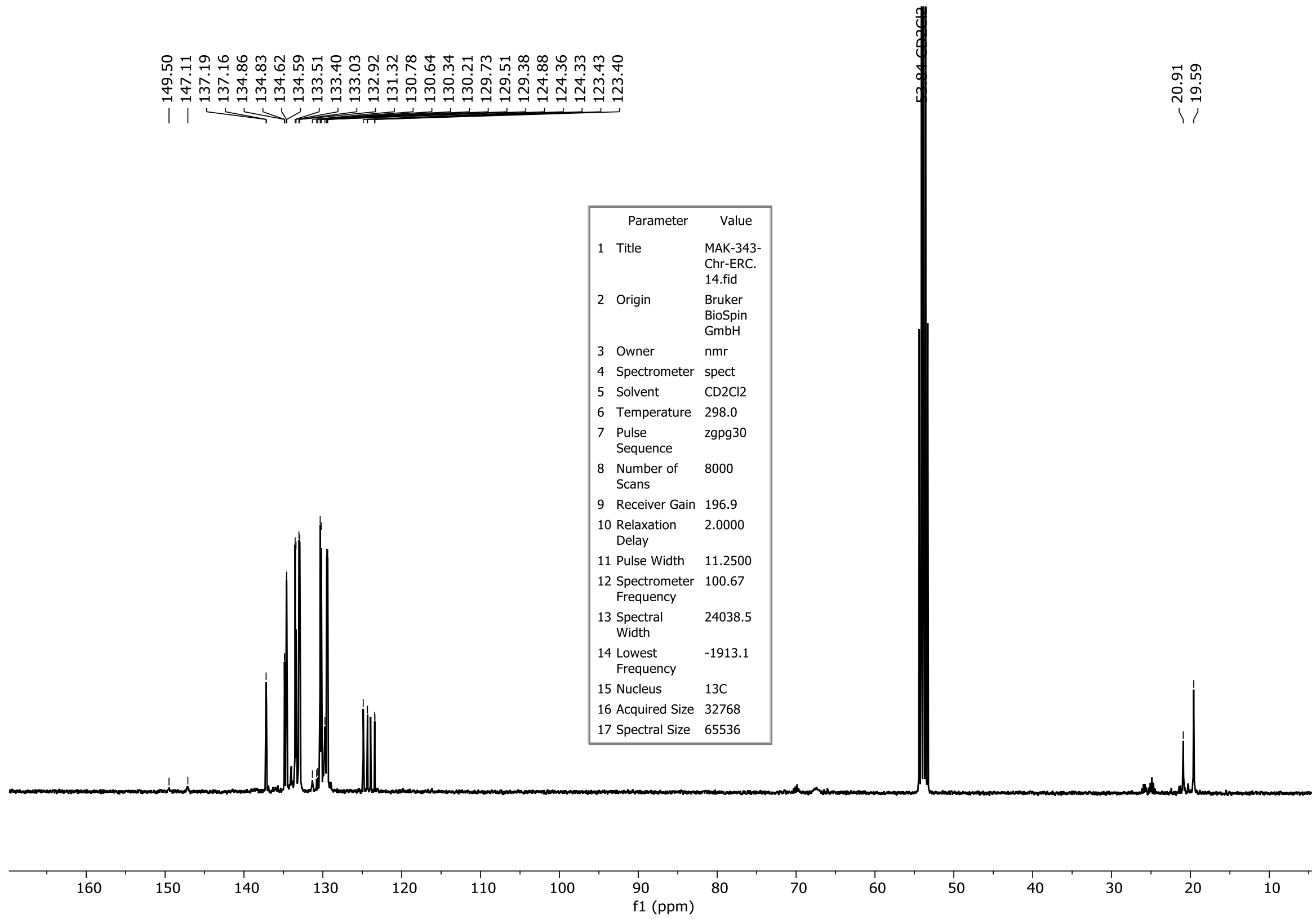

Figure S33.$^{13}C\{^1H\}$ NMR spectrum of compound **4e** in DCM-d_2.

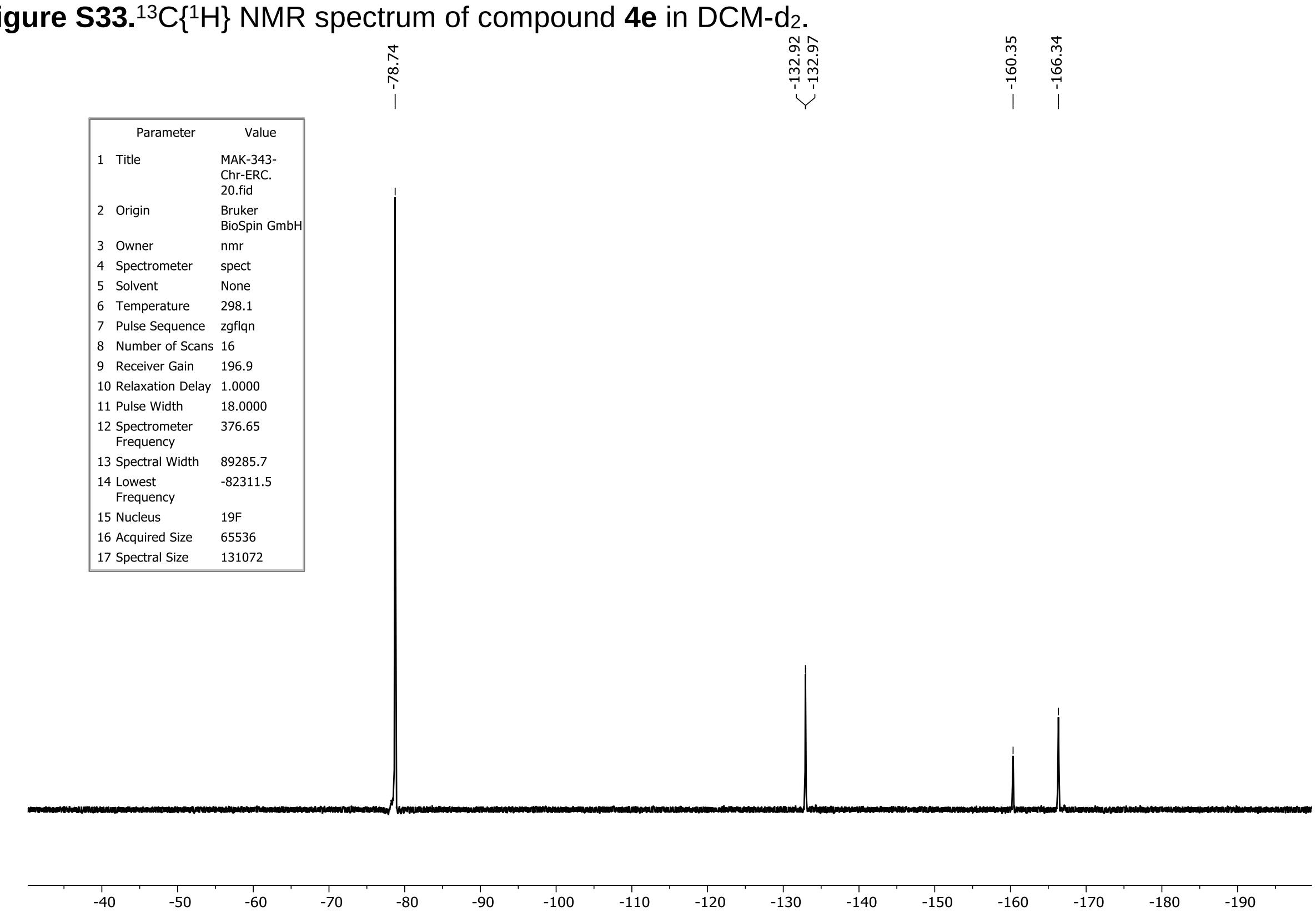

Figure S34.$^{19}F\{^1H\}$ NMR spectrum of compound **4e** in DCM-d_2.

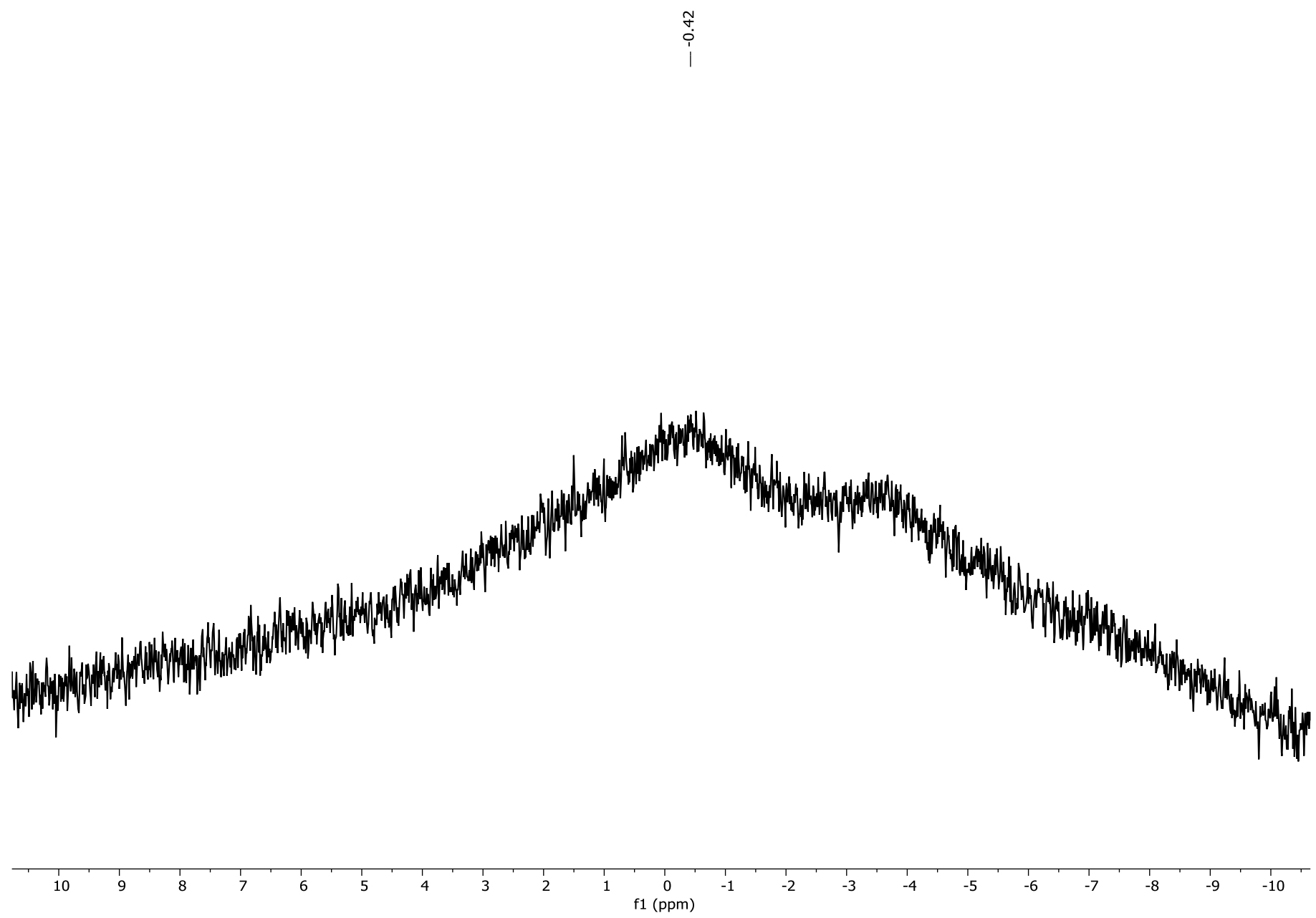

Figure S35. $^{11}B\{^{1}H\}$ NMR spectrum of compound **4e** in DCM-d_2.

2.5 NMR spectra of compound 5

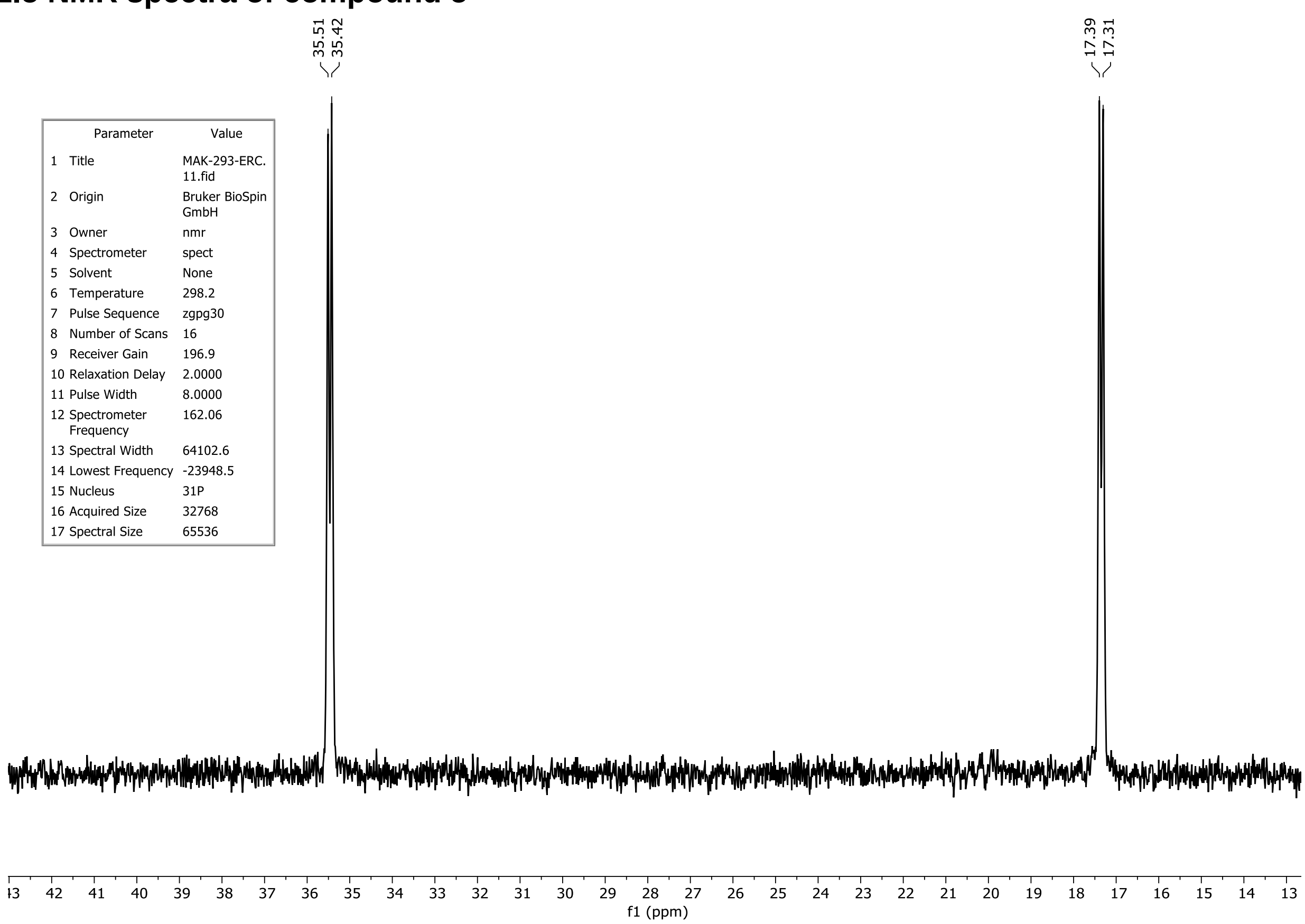

Figure S36. $^{31}P\{^{1}H\}$ NMR spectrum of compound **5** in DCM-d_2.

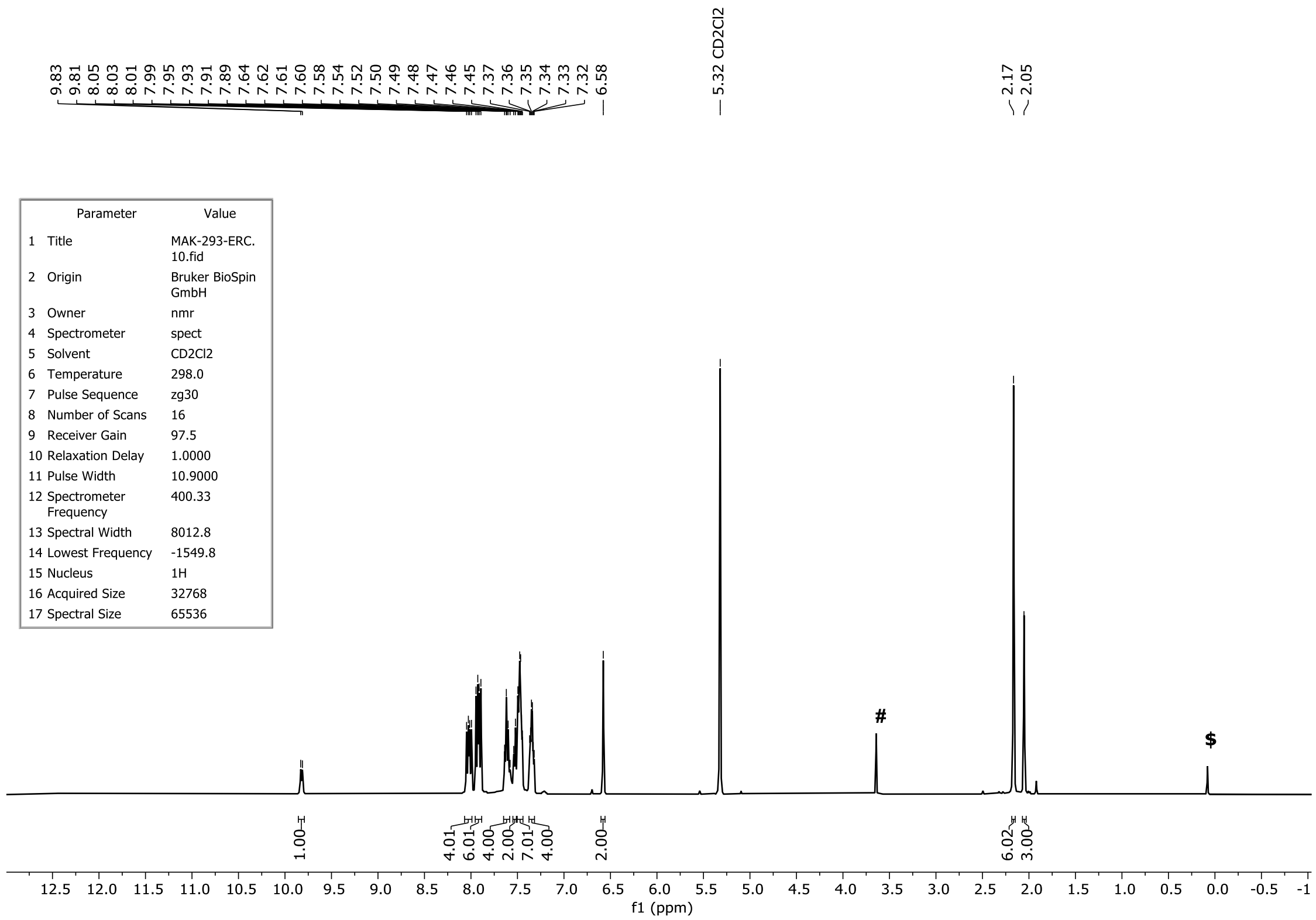

Figure S37. ^{1}H NMR spectrum of compound **5** in DCM-d_2. The ^{1}H NMR spectrum shows residues of 1,4-dioxne and grease (δ = 3.6 (#) and 0.09 ($) ppm).

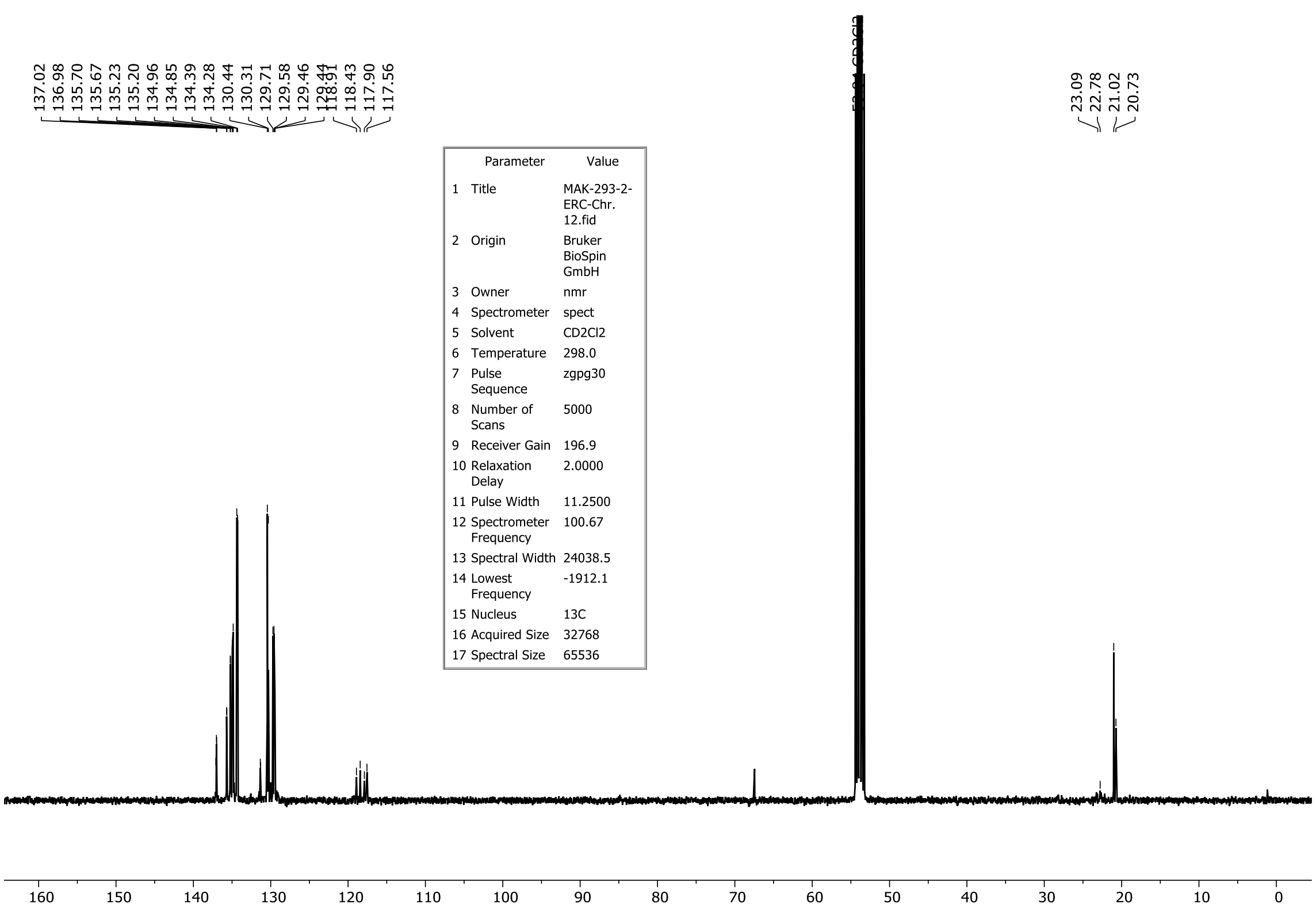

Figure S38. $^{13}C\{^{1}H\}$ NMR spectrum of compound **5** in DCM-d_2.

3. Crystal structure determination.

3.1 General information

High-quality single crystals were hand-picked under polarized optical microscopy and mounted an inert oil (perfluoropolyalkylether) on the diffractometer. The data collection was performed at 100 K. X-ray intensity data measurements of all compounds were carried out on an Oxford SuperNova diffractometer with graphite-monochromatized (CuK_{α}= 1.54184 Å) radiation. The X-ray generator was operated at 50 kV and 30 mA.
All the structures were solved using direct methods, refined by the full-matrix least-squares on F^2 using the Shelx software package.[6–9] Non-hydrogen atoms were refined anisotropically. Hydrogen atoms were inserted at ideal positions and refined as riding atoms.
Crystallographic data including structure factors have been deposited with the Cambridge Crystallographic Data Centre as supplementary publication no. CCDC-2536593 to CCDC-2536601. Copies of the data can be gained free of charge on application to Cambridge Crystallographic Data Centre, 12 Union Road, Cambridge CB2 1EZ, UK; [fax: (+44) 1223-336-033; email: deposit@ccdc.cam.ac.uk].

Table S1. Data collection and structure refinement details for compounds **[$Y_{N\text{-}Tol}$-GeCl]**, **2**, and **3**.

Compound	**[$Y_{N\text{-}Tol}$-GeCl]**	**2**	**3**
CCDC No.	2536600	2536593	2536601
Formula	$C_{38}H_{32}Cl_1Ge_1N_1P_2$	$C_{80}H_{72}Cl_2Ge_2N_2O_2P_4$	$C_{58}H_{37}B_1Cl_2F_{15}Ge_1N_1O_1P_2$
Formula weight [g.mol^{-1}]	672.62	1433.35	1265.12
Temperature [K]	100.0(2)	100.0(2)	100.0(2)
Wavelength [Å]	1.54184	1.54184	1.54184
Crystal system	Triclinic	Monoclinic	Triclinic
Space group	P-1	$P2_1/c$	P-1
a [Å]	9.4395(3)	13.84690(10)	14.5867(3)
b [Å]	10.5984(4)	19.70040(10)	14.5879(3)
c [Å]	17.1227(4)	14.30170(10)	15.6880(4)
α [°]	95.708(3)	90	65.990(2)
β [°]	97.457(2)	118.2690(10)	83.312(2)
γ [°]	106.193(3)	90	77.600(2)
Volume [Å^3]	1614.51(9)	3436.05(5)	2977.68(13)
Z	2	2	2
Calc. density [Mg•m^{-3}]	1.384	1.385	1.411
μ [mm^{-1}]	3.196	3.060	2.799
F(000)	692.0	1480.0	1272

Crystal dimensions [mm]	0.250 x 0.080 x 0.040	0.090 x 0.070 x 0.050	0.337 x 0.243 x 0.154
Theta range [°]	4.389 to 67.999	3.624 to 67.998	3.085 to 67.078
Index ranges	$-11 \le h \le 10$	$-16 \le h \le 15$	$-14 \le h \le 17$
	$-12 \le k \le 12$	$-23 \le k \le 23$	$-17 \le k \le 17$
	$-20 \le l \le 15$	$-17 \le l \le 16$	$-18 \le l \le 18$
Reflections collected	17616	48274	39224
Independent reflections	5848 [R_{int} = 0.0422]	6261 [R_{int} = 0.0380]	10644 [R_{int} = 0.0369]
Data/Restraints/Parameter	5848 / 0 / 389	6261 / 96 / 594	10644 / 96 / 771
Goodness-of-fit on F^2	1.049	1.052	1.031
Final R indices	R1 = 0.0352,	R1 = 0.0358,	R1 = 0.0340,
[I>2sigma(I)]	wR2 = 0.0949	wR2 = 0.0943	wR2 = 0.0916
R indices (all data)	R1 = 0.0378,	R1 = 0.0397,	R1 = 0.0361,
	wR2 = 0.0968	wR2 = 0.0974	wR2 = 0.0934
Largest diff. peak and hole	0.752 and –0.548	0.520 and –0.428	0.753 and –0.508

Table S2. Data collection and structure refinement details for compounds **4a**, **4b**, and **4c**.

Compound	**4a**	**4b**	**4c**
CCDC No.	2536594	2536598	2536597
Formula	$C_{146}H_{100}B_2F_{48}Ge_2N_2O_2P_4Cl_4$	$C_{80}H_{72}F_{12}Ge_2N_2O_2P_4Sb_2$	$C_{82}H_{72}F_6Ge_2N_2O_8P_4S_2$
Formula weight [g.mol^{-1}]	3258.75	1833.95	1660.59
Temperature [K]	100.0(2)	100.0(2)	100.0(2)
Wavelength [Å]	1.54184	1.54184	1.54184
Crystal system	Triclinic	Triclinic	Triclinic
Space group	P-1	P-1	P-1
a [Å]	12.7671(4)	10.8164(3)	9.8887(2)
b [Å]	14.9702(3)	14.1727(5)	14.5250(2)
c [Å]	20.4084(4)	15.0268(4)	15.3385(2)
α [°]	80.120(2)	99.846(3)	69.1710(10)
β [°]	89.554(2)	98.470(2)	88.974(2)
γ [°]	76.732(2)	104.725(3)	76.829(2)
Volume [Å^3]	3738.12(16)	2150.67(12)	2000.14(6)

Z	1	1	1
Calc. density [Mg•m^{-3}]	1.448	1.416	1.397
μ [mm^{-1}]	2.541	7.024	2.751
F(000)	1640	916	852
Crystal dimensions [mm]	0.180 x 0.110 x 0.080	0.106 x 0.051 x 0.042	0.900 x 0.280 x 0.240
Theta range [°]	3.080 to 67.999	3.304 to 67.079	3.090 to 67.993
Index ranges	–15 ≤ h ≤ 14	–12 ≤ h ≤ 12	–10 ≤ h ≤ 11
	–17 ≤ k ≤ 17	–15 ≤ k ≤ 16	–17 ≤ k ≤ 17
	–24 ≤ l ≤ 23	–17 ≤ l ≤ 17	–18 ≤ l ≤ 18
Reflections collected	44940	22677	24274
Independent reflections	13523 [R_{int} = 0.0657]	7594 [R_{int} = 0.0615]	7277 [R_{int} = 0.0415]
Data/Restraints/Parameter	13523 / 0 / 949	7594 / 0 / 472	7277 / 122 / 554
Goodness-of-fit on F^2	1.070	1.060	1.035
Final R indices	R1 = 0.0532,	R1 = 0.0618,	R1 = 0.0392,
[I>2sigma(I)]	wR2 = 0.1435	wR2 = = 0.1668	wR2 = 0.1012
R indices (all data)	R1 = 0.0619,	R1 = 0.0682,	R1 = 0.0426,
	wR2 = 0.1496	wR2 = 0.1715	wR2 = 0.1033
Largest diff. peak and hole	0.777 and –0.766	2.087 and –1.787	0.655 and –0.548

Table S3. Data collection and structure refinement details for compounds **4d**, **4e**, and **5**.

Compound	**4d**	**4e**	**5**
CCDC No.	2536595	2536596	2536599
Formula	$C_{130}H_{88}B_2Cl_2F_{30}Ge_2N_2O_2P_4$	$C_{132}H_{88}B_2F_{36}Ge_2N_2O_8P_4S_2$	$C_{40}H_{39}Cl_4Ge_1N_1P_2$
Formula weight [g.mol^{-1}]	2641.60	2868.84	810.05
Temperature [K]	100.0(2)	100.0(2)	110.0(2)
Wavelength [Å]	1.54184	1.54184	1.54184
Crystal system	Triclinic	Triclinic	Triclinic
Space group	P-1	P-1	P-1
a [Å]	14.5312(2)	14.2482(2)	9.3404(2)
b [Å]	15.4248(2)	15.7912(2)	13.3763(2)
c [Å]	15.5304(2)	16.6635(2)	17.9969(3)

α [°]	78.9500(10)	111.6770(10)	92.1590(10)
β [°]	67.83600(10)	100.2150(10)	100.5880(10)
γ [°]	63.6070(10)	110.4850(10)	104.5070(10)
Volume [Å^3]	2886.55(7)	3054.61(7)	2131.49(7)
Z	1	1	2
Calc. density [Mg•m^{-3}]	1.520	1.560	1.262
μ [mm^{-1}]	2.498	2.441	4.195
F(000)	1336	1448	832
Crystal dimensions [mm]	0.360 x 0.160 x 0.110	0.285 x 0.185 x 0.158	0.230 x 0.160 x 0.130
Theta range [°]	3.074 to 67.998	3.049 to 67.998	2.507 to 67.999
Index ranges	–15 ≤ h ≤ 17	–15 ≤ h ≤ 17	–11 ≤ h ≤ 11
	–18 ≤ k ≤ 18	–18 ≤ k ≤ 18	–16 ≤ k ≤ 13
	–18 ≤ l ≤ 18	–20 ≤ l ≤ 20	–21 ≤ l ≤ 19
Reflections collected	34119	38824	24487
Independent reflections	10495 [R_{int} = 0.0329]	11080 [R_{int} = 0.0359]	7748 [R_{int} = 0.0378]
Data/Restraints/Parameter	10495 / 0 / 788	11080 / 125 / 916	7748 / 0 / 440
Goodness-of-fit on F^2	1.033	1.039	1.030
Final R indices	R1 = 0.0331,	R1 = 0.0371,	R1 = 0.0408,
[I>2sigma(I)]	wR2 = 0.0884	wR2 = = 0.0904	wR2 = = 0.1059
R indices (all data)	R1 = 0.0358,	R1 = 0.0419,	R1 = 0.0455,
	wR2 = 0.0905	wR2 = 0.0930	wR2 = 0.1091
Largest diff. peak and hole	0.478 and –0.338	1.021 and –0.514	0.811 and –0.717

3.2. Crystal structure analysis of the chlorogermylene, 2, and 6

Crystal structure analysis of [YN-Tol-GeCl]

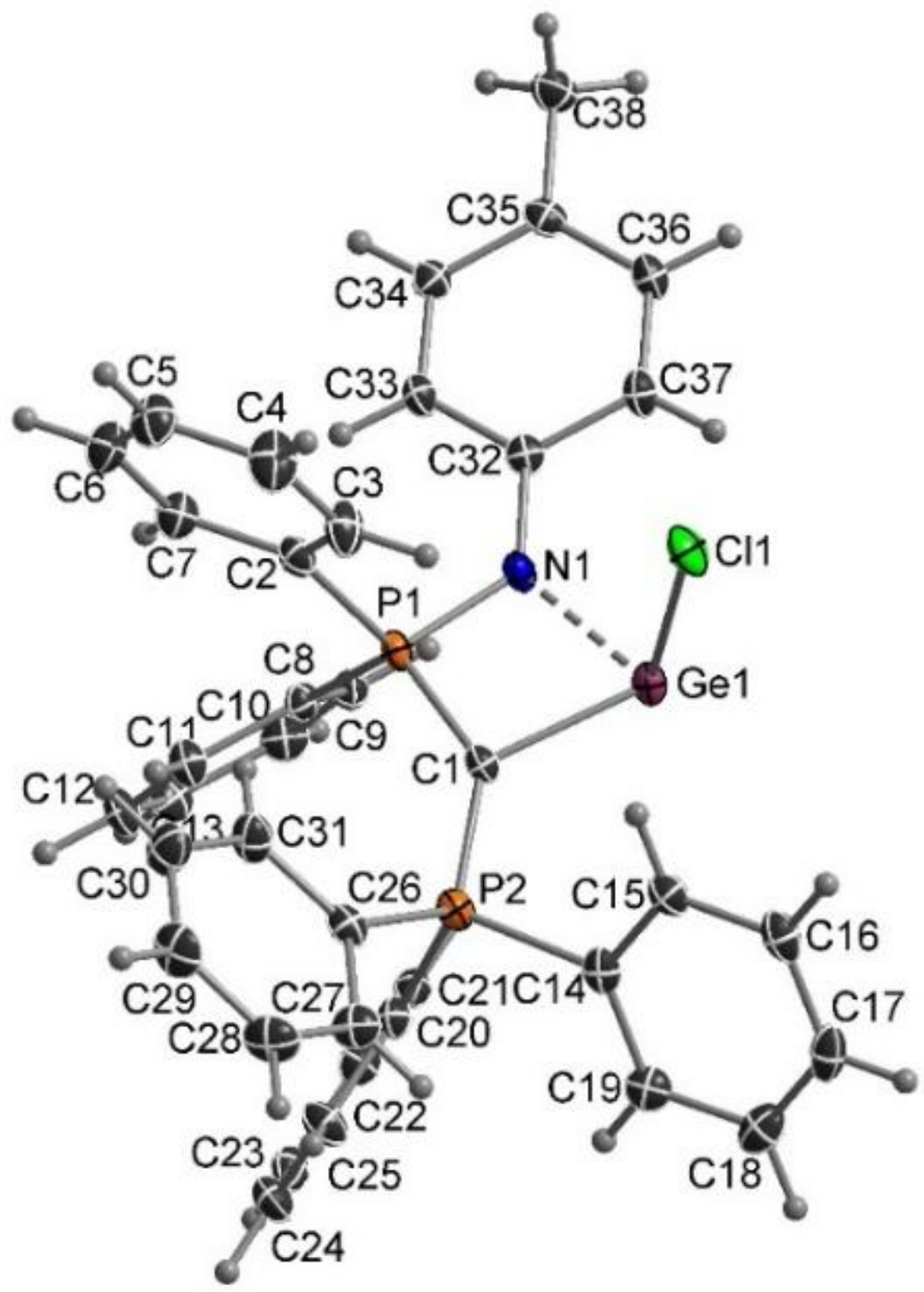

Figure S39. Molecular structure of compound **[$Y_{N\text{-}Tol}$-GeCl]**. Thermal ellipsoids at 50% probability level. Selected bond lengths [Å] and angles [°]: P1–C1 1.705(2), P2–C1 1.678(2), P1–N1 1.638(2), N1–Ge1 2.004(2), C1–Ge1 2.039(2), Ge1–Cl1 2.382(5), P1–C1–P2 135.9(1).

Crystal structure analysis of 2

All hydrogen atoms were placed in ideal positions. **2** was refined in ShelXle, and the crystal structure contained a disordered mesityl ($C_6H_2Me_3$) and phenyl groups. The disorders were modelled using SIMU and DELU restraints and refined using the PART instructions. The free variable for the two phenyl groups of the $Ph_2P{=}N$ moiety was optimized to occupancies of 45% /55% and 50%/ 50%, respectively. The free variable for one phenyl group of the PPh_3 moiety was optimized to occupancies of 57%/43%.

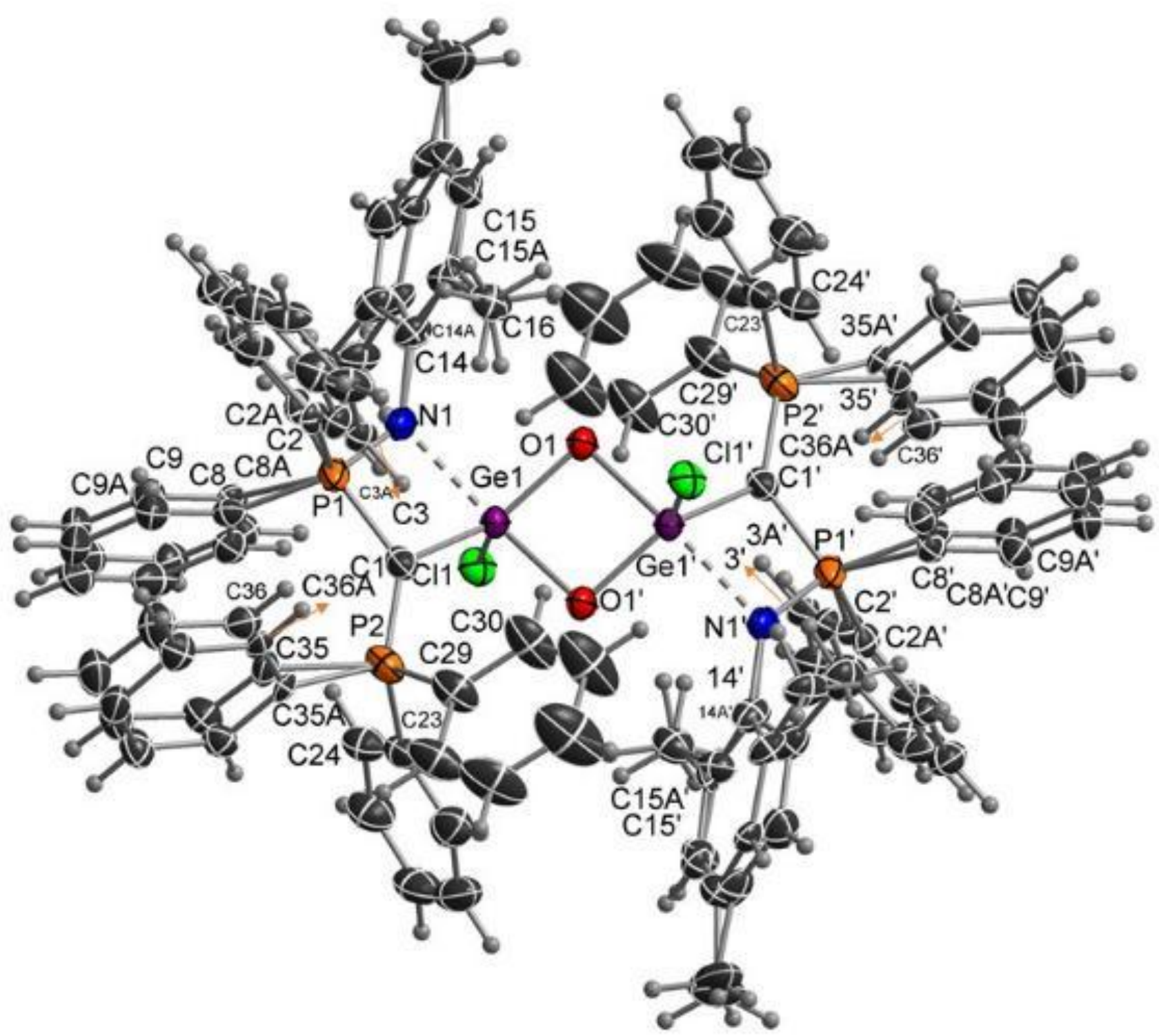

Figure S40. Molecular structure of compound **2**. Thermal ellipsoids at 50% probability level. Selected bond lengths [Å] and angles [º]: P1–C1/P1'–C1' 1.714(2), P2–C1/P2'–C1' 1.694(2), P1–N1/P1'–N1' 1.619(2), C1–Ge1/C1'–Ge1' 1.939(2), N1–Ge1/N1'–Ge1' 2.073(2), Ge1–Cl1/Ge1'–Cl1' 2.240(6), Ge1–O1/Ge1'–O1' 1.788(2), Ge1–O1'/Ge1'–O1 1.857(2), P1–C1–P2/P1'–C1'–P2' 133.4(1), O1–Ge1–O1'/O1–Ge1'–O1' 84.7(7), Ge1–O1–Ge1'/Ge1'–O1'–Ge1 95.2(7).

Crystal structure analyses of the 3

All hydrogen atoms were placed in ideal positions. **3** was refined in ShelXle, and highly disordered solvent molecules (toluene and hexane) were removed via the SQUEEZE function of the PLATON software package.

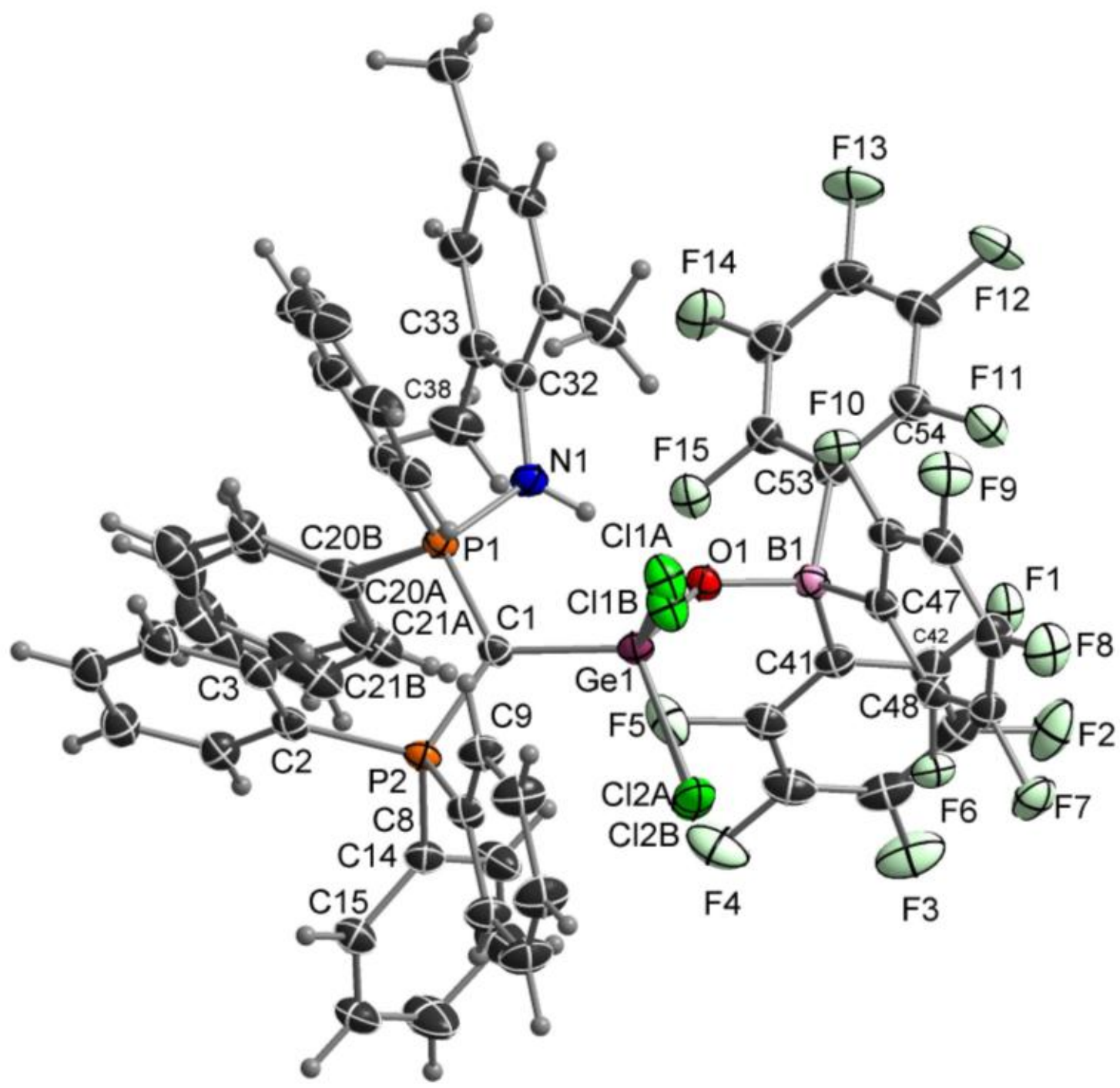

Figure S41. Molecular structure of compound **3**. Thermal ellipsoids at 50% probability level. Selected bond lengths [Å] and angles [°]: P1–C1 1.737(2), P2–C1 1.746(2), P1–N1 1.642(1), C1–Ge1 1.911(2), Ge1–Cl1A 2.185(2), Ge1–Cl1B 1.997(1), Ge1–Cl2A 2.131(1), Ge1–Cl2B 2.189(1), Ge1–O1 1.728(1), O1–B1 1.502(2), P1–C1–P2 120.9(1), 84.7(7), C1–Ge1–O1 112.7(6) Ge1–O1–B1 134.7(1).

3.3. Crystal structure analyses of the 1,3-digermadioxetane dications 4a-e

Crystal structure analysis of compound 4a

All hydrogen atoms were placed in ideal positions. **4a** was refined in ShelXle, and a highly disordered solvent molecule (pentane) was removed via the SQUEEZE function of the PLATON software package.

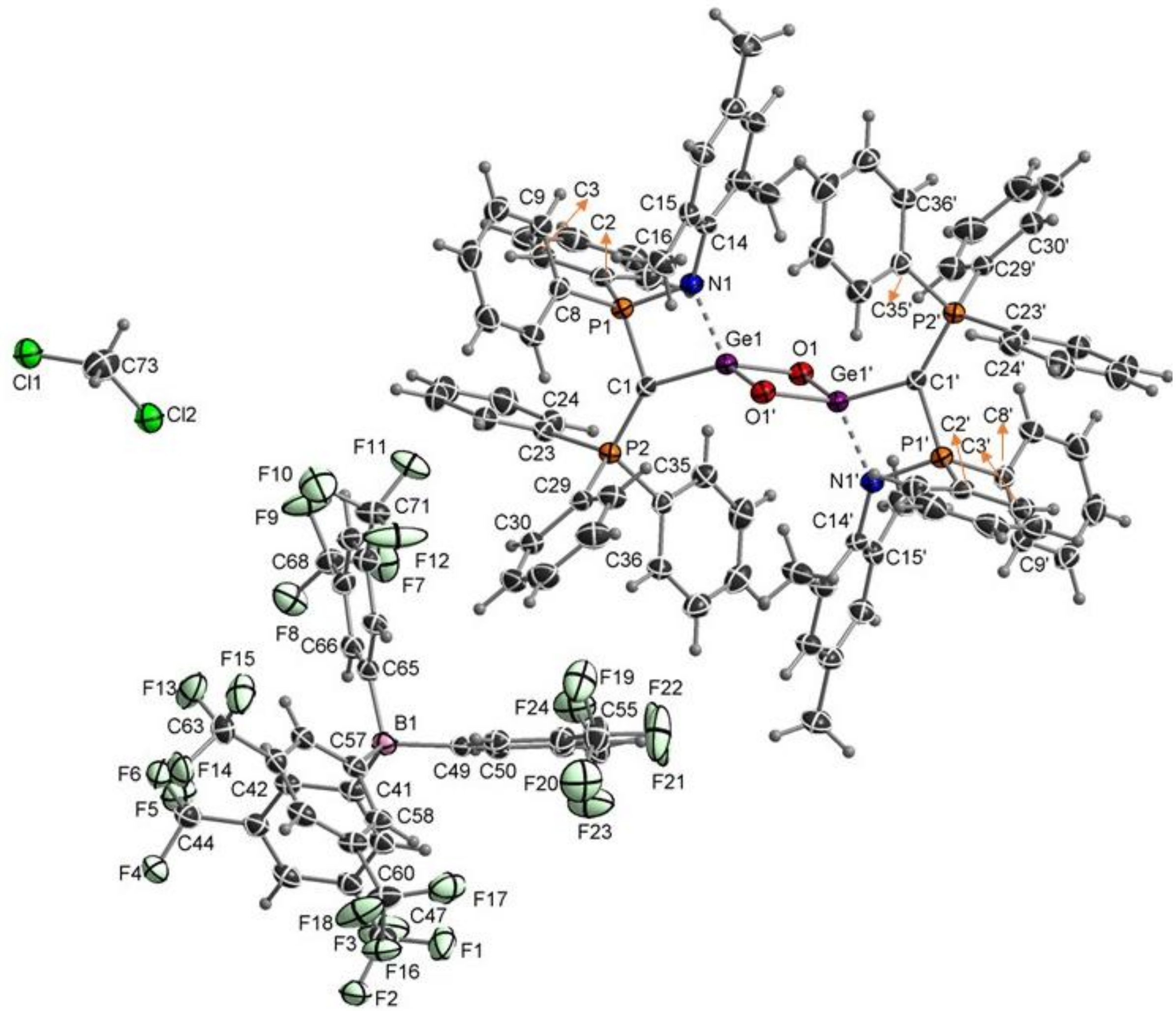

Figure S42. Molecular structure of compound **4a**. Thermal ellipsoids at 50% probability level. Selected bond lengths [Å] and angles [°]: P1–C1/P1'–C1' 1.735(3), P2–C1/P2'–C1' 1.707(3), P1–N1/P1'–N1' 1.673(2), C1–Ge1/C1'–Ge1' 1.874(3), N1–Ge1/N1'–Ge1' 1.860(2), Ge1–O1/Ge1'–O1' 1.795(2), Ge1–O1'/Ge1'–O1 1.799(2), P1–C1–P2/P1'–C1'–P2' 131.2(2), O1–Ge1–O1'/O1–Ge1'–O1' 87.7(9), Ge1–O1–Ge1'/Ge1'–O1'–Ge1 92.3(9).

Crystal structure analysis of compound 4b

All hydrogen atoms were placed in ideal positions. **4b** was refined in ShelXle, and a highly disordered solvent molecules (DCM and pentane) were removed via the SQUEEZE function of the PLATON software package.

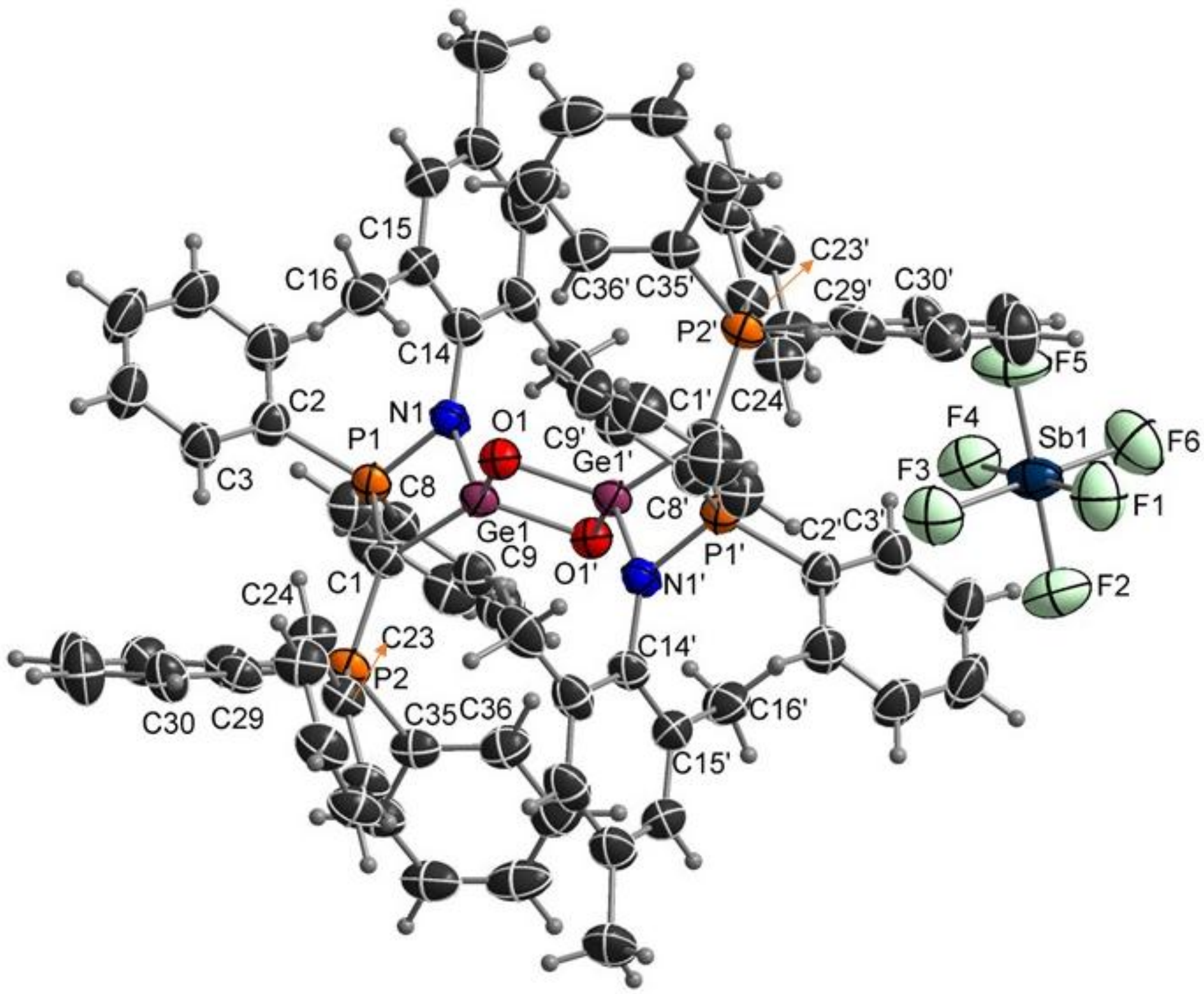

Figure S43. Molecular structure of compound **4b**. Thermal ellipsoids at 50% probability level. Selected bond lengths [Å] and angles [°]: P1–C1/P1'–C1' 1.732(5), P2–C1/P2'–C1' 1.721(5), P1–N1/P1'–N1' 1.672(4), C1–Ge1/C1'–Ge1' 1.874(5), N1–Ge1/N1'–Ge1' 1.846(4), Ge1–O1/Ge1'–O1' 1.778(3), Ge1–O1'/Ge1'–O1 1.817(3), P1–C1–P2/P1'–C1'–P2' 131.7(3), O1–Ge1–O1'/O1–Ge1'–O1' 87.7(2), Ge1–O1–Ge1'/Ge1'–O1'–Ge1 92.3(2).

Crystal structure analysis of compound 4c

All hydrogen atoms were placed in ideal positions. **4c** was refined in ShelXle, and a highly disordered solvent molecules (DCM and pentane) were removed via the SQUEEZE function of the PLATON software package.

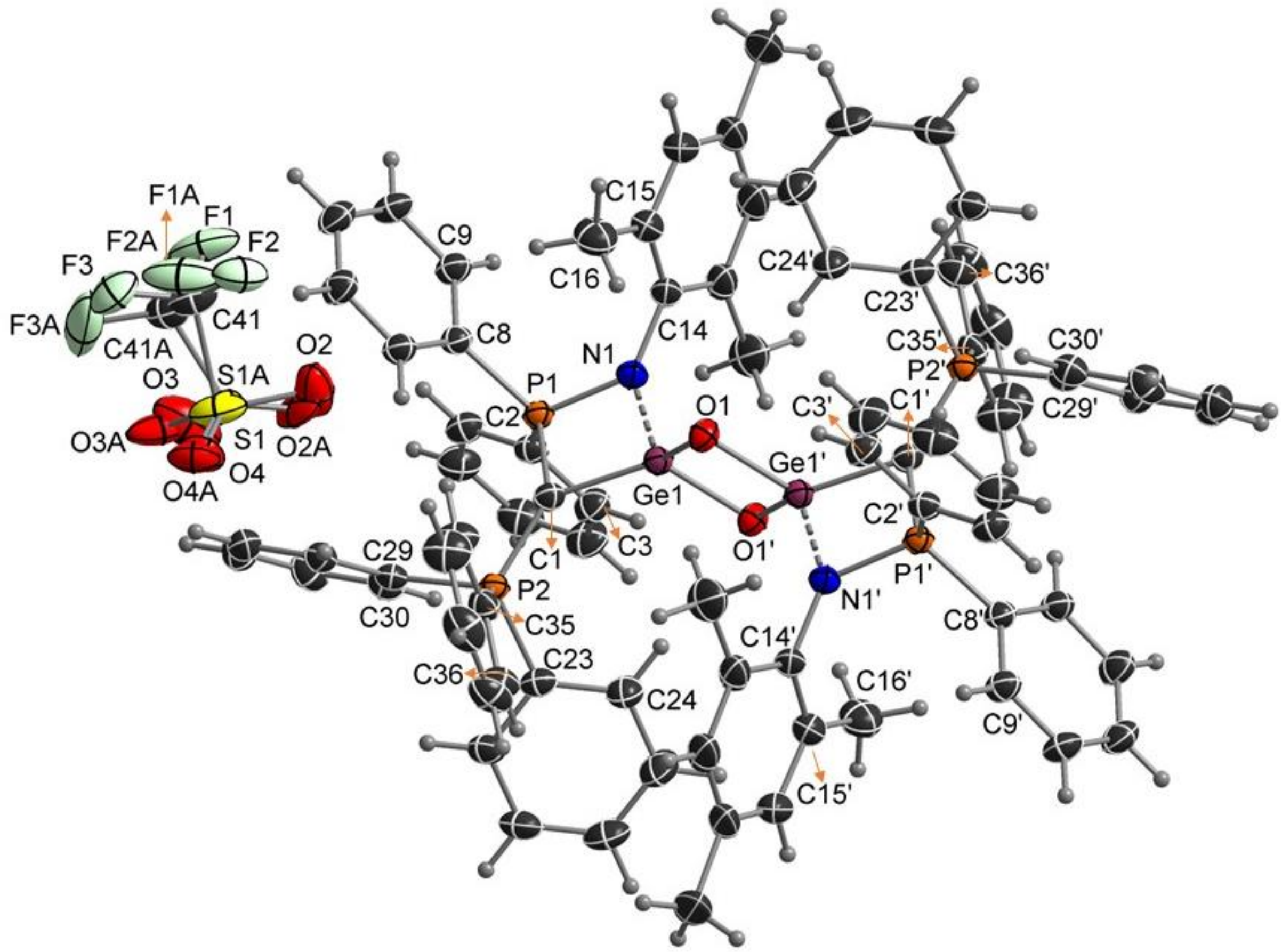

Figure S44. Molecular structure of compound **4c**. Thermal ellipsoids at 50% probability level. Selected bond lengths [Å] and angles [º]: P1–C1/P1'–C1' 1.741(2), P2–C1/P2'–C1' 1.717(2), P1–N1/P1'–N1' 1.675(2), C1–Ge1/C1'–Ge1' 1.877(2), N1–Ge1/N1'–Ge1' 1.855(2), Ge1–O1/Ge1'–O1' 1.791(2), Ge1–O1'/Ge1'–O1 1.814(2), P1–C1–P2/P1'–C1'–P2' 131.1(1), O1–Ge1–O1'/O1–Ge1'–O1' 87.5(7), Ge1–O1–Ge1'/Ge1'–O1'–Ge1 92.4(7).

Crystal structure analysis of compound 4d

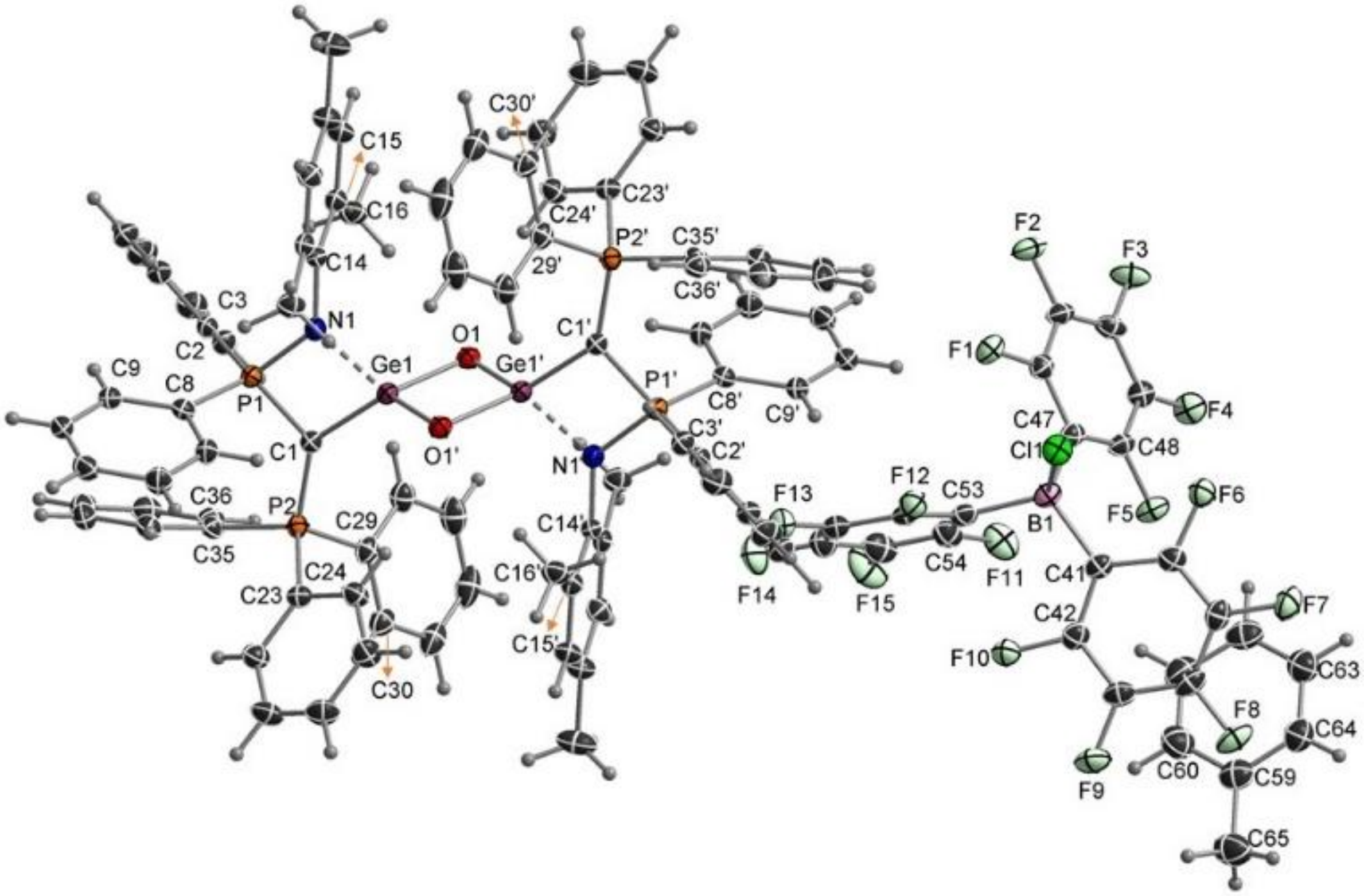

Figure S45. Molecular structure of compound **4d**. Thermal ellipsoids at 50% probability level. Selected bond lengths [Å] and angles [°]: P1–C1/P1'–C1' 1.736(2), P2–C1/P2'–C1' 1.715(2), P1–N1/P1'–N1' 1.673(2), C1–Ge1/C1'–Ge1' 1.885(2), N1–Ge1/N1'–Ge1' 1.859(1), Ge1–O1/Ge1'–O1' 1.796(1), Ge1–O1'/Ge1'–O1 1.805(1), P1–C1–P2/P1'–C1'–P2' 130.7(1), O1–Ge1–O1'/O1–Ge1'–O1' 87.6(6), Ge1–O1–Ge1'/Ge1'–O1'–Ge1 92.3(6).

Crystal structure analysis of compound 4e

All hydrogen atoms were placed in ideal positions. **4e** was refined in ShelXle, and the crystal structure contained a disordered toluene molecule. The disorders were modelled using SIMU and DELU restraints and refined using the PART instructions, and the free variables, which was optimized to occupancies of 78% and 22%.

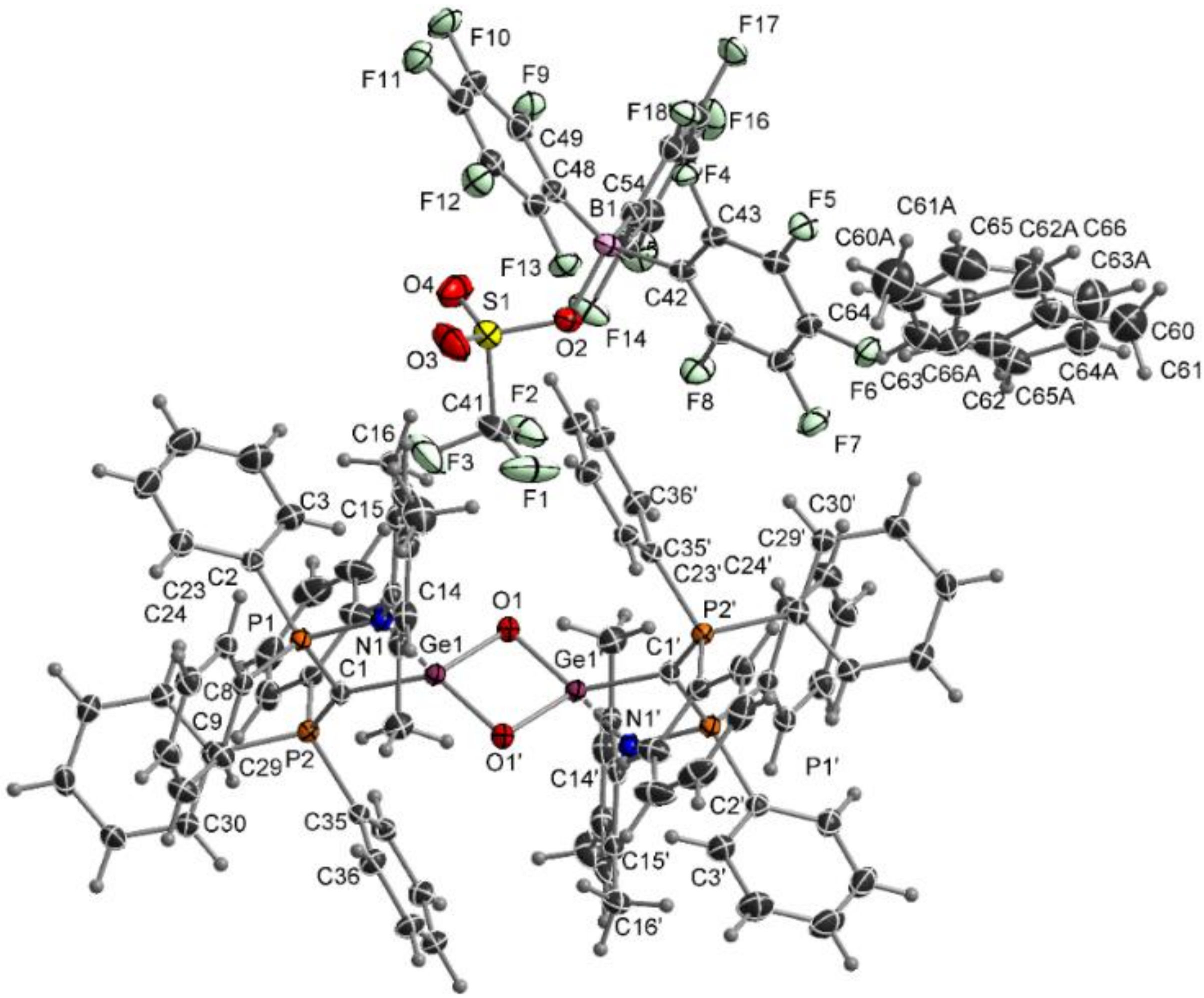

Figure S46. Molecular structure of compound **4e**. Thermal ellipsoids at 50% probability level. Selected bond lengths [Å] and angles [º]: P1–C1/P1'–C1' 1.734(2), P2–C1/P2'–C1' 1.713(2), P1–N1/P1'–N1' 1.675(2), C1–Ge1/C1'–Ge1' 1.899(2), N1–Ge1/N1'–Ge1' 1.867(2), Ge1–O1/Ge1'–O1' 1.794(1), Ge1–O1'/Ge1'–O1 1.801(1), P1–C1–P2/P1'–C1'–P2' 133.4(1), O1–Ge1–O1'/O1–Ge1'–O1' 87.2(7), Ge1–O1–Ge1'/Ge1'–O1'–Ge1 92.7(7).

3.4. Crystal structure analysis of compound 5

All hydrogen atoms were placed in ideal positions. **5** was refined in ShelXle, and a highly disordered solvent molecules (DCM and hexane) were removed via the SQUEEZE function of the PLATON software package.

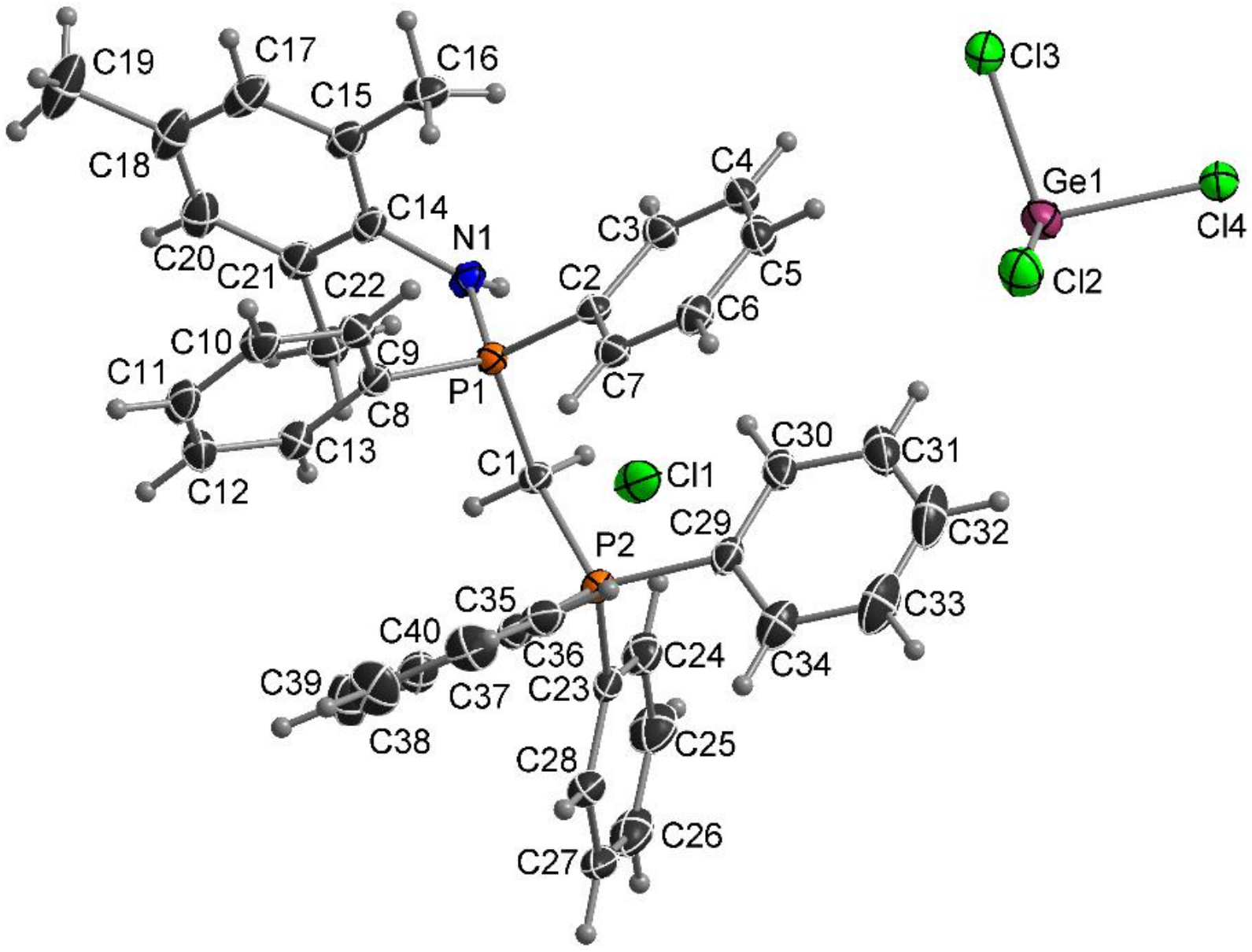

Figure S47. Molecular structure of compound **5**. Thermal ellipsoids at 50% probability level. Selected bond lengths [Å] and angles: P1–C1 1.818(2), P2–C1 1.806(2), P1–N1 1.633(2), P1–C1–P2 125.0(1).

4. Computational results

4.1 General information

All calculations were performed without symmetry restrictions. Starting coordinates were obtained with GaussView 6.1.1[10] respectively from the crystal structure analysis if available. The geometry optimizations were performed using Density Functional Theory (DFT) and carried out using the ORCA 6.0.1[11] program package utilizing the PBE0[12] functional in combination with def2-tzvpp basis set[13] and Grimme's D3 dispersion correction with Becke-Johnson damping[14] and the Conductor-like Polarizable Continuum Model (CPCM)[15] as implicit solvent model using THF. Harmonic vibrational frequency analyses were performed at the same level of theory and showed no imaginary frequencies. Single point calculations were performed on the ωB97M-V functional[16] with the def2-TZVPP basis set for **2** and **8** and def2-QZVP for **3** and **3'** utilizing the Universal Solvation Model (SMD)[17] using THF. The NBO calculations[18] were performed on program version 7.0 additionally to the IBO calculations[19]. All ORCA calculations ran with defgrid3 and verytightscf keywords to prevent imaginary frequencies. Quantum theory of atoms in molecules (QTAIM)[20] analysis was carried out using MultiWFN version 3.8[21] which was also used to visualize the topological properties of the molecules. The other results were visualized using VMD 1.9.4a53[22].

4.2 Energy optimization

Compound **2**

The original structure of **2** was obtained by single crystal XRD analysis. A disordered methyl group in the mesityl ring bound to the nitrogen was resolved manually. Initially a set of functionals were tested to find the optimal structure with the similarity of the bond lengths and angles to the XRD crystals as benchmark. The r²SCAN-3c[23] and ωB97M-3c hybrid functionals were tested as well as the revTPSS[24], PW6B95[25], revPBE[26] and PBE0 functionals with parameters as specified above. For technical reasons the calculations had to be carried out with the Grimme's D4 dispersion correction for the revTPSS functional.

Table S4. Comparison of experimental data and computational data using various functionals on bond lengths [Å] and angles [°] of **2**.

Bond lengths [Å]	**XRD**	r2SCAN-3c	wB97x-3c	revTPSS	PW6B95	revPBE	**PBE0**
P12-C5	**1.691**	1.707	1.702	1.695	1.687	1.699	**1.690**
C5-P8	**1.716**	1.725	1.720	1.711	1.702	1.717	**1.705**
P8-N6	**1.622**	1.651	1.634	1.639	1.623	1.642	**1.627**
C5-Ge2	**1.940**	1.955	1.941	1.945	1.938	1.958	**1.945**
Ge2-Cl1	**2.240**	2.300	2.287	2.283	2.267	2.301	**2.259**
Ge2-O3	**1.787**	1.806	1.797	1.806	1.792	1.819	**1.792**
Ge2-O4	**1.857**	1.878	1.884	1.877	1.855	1.891	**1.860**

O4-Ge7	**1.787**	1.806	1.797	1.806	1.792	1.819	**1.787**
O3-Ge7	**1.857**	1.878	1.884	1.877	1.855	1.891	**1.860**
Ge7-Cl81	**2.240**	2.300	2.287	2.283	2.267	2.301	**2.272**
Ge7-C84	**1.940**	1.955	1.941	1.945	1.938	1.958	**1.937**
C84-P82	**1.716**	1.725	1.720	1.711	1.702	1.717	**1.709**
P82-N83	**1.621**	1.651	1.634	1.639	1.623	1.642	**1.623**
C84-P88	**1.691**	1.707	1.702	1.695	1.687	1.699	**1.690**
Mean Dev.	**-**	0.012	0.012	0.012	0.008	0.015	**0.007**
Angles [°]							
P12-C5-P8	**133.377**	131.094	131.253	130.862	132.685	130.882	**131.961**
P12-C5-Ge2	**129.058**	131.425	130.784	131.969	130.714	131.879	**131.115**
P8-C5-Ge2	**95.449**	96.153	96.556	95.766	95.363	95.989	**95.568**
C5-P8-N6	**95.259**	94.071	94.717	94.766	95.102	95.143	**94.989**
C5-Ge2-O4	**99.749**	99.524	101.019	99.156	100.198	99.397	**98.357**
Mean Dev.		1.179	1.146	1.095	0.679	0.985	**1.158**

The optimizations using revTPSS, PW6B95, revPBE display imaginary frequencies and therefore cannot be considered a ground state. As the mean deviation of those are considered quite high, no corrected optimization was performed to save computational cost. Since PBE0 shows the lowest mean deviation in bond lengths and an acceptable value in bond angles but is also overall the best in the optimization of **3** (Table S5) it was chosen as the overall functional to be used in all optimizations performed in this work.

Compound 3

For compound 3, a similar strategy as described above was applied.

Table S5. Comparison of experimental data and computational data using various functionals on bond lengths [Å] and angles [°] of **3** and the theoretical values for **3'** on the PBE0/def2-TZVPP/D3BJ/CPCM(THF) level of theory. Please remark that the atom indexing is different in **3'**.

Bond lengths [Å]	**XRD**	r2SCAN-3c	wB97x-3c	revTPSS	PW6B95	revPBE	**PBE0**	**3'**
P10-C5	**1.738**	1.748	1.743	1.732	1.725	1.738	**1.730**	**1.727**
P10-N11	**1.643**	1.677	1.656	1.667	1.648	1.672	**1.649**	**1.649**

P6-C5	**1.745**	1.755	1.749	1.738	1.734	1.743	**1.735**	**1.715**
C5-Ge1	**1.909**	1.922	1.919	1.913	1.909	1.926	**1.911**	**1.931**
N11-H45	**0.880**	1.025	1.016	1.033	1.016	1.033	**1.023**	**1.069**
Ge1-Cl2	**2.171**	2.199	2.193	2.188	2.175	2.203	**2.174**	**2.213**
Ge1-Cl3	**2.131**	2.180	2.175	2.170	2.162	2.185	**2.157**	**2.194**
Ge1-O4	**1.730**	1.745	1.742	1.750	1.734	1.763	**1.736**	**1.688**
O4-B16	**1.503**	1.507	1.498	1.513	1.494	1.520	**1.498**	**-**
Mean Dev.		0.028	0.028	0.030	0.030	0.029	**0.030**	**-**
Angles [°]								
Cl2-Ge1-Cl3	**102.536**	103.626	103.897	104.101	104.881	104.109	**104.280**	**100.446**
Ge1-O4-B16	**134.644**	131.355	133.442	129.642	131.411	130.568	**132.357**	**-**
C5-Ge1-O4	**112.859**	113.565	107.313	108.349	115.351	112.414	**113.289**	**113.594**
P6-C5-P10	**120.769**	121.460	121.415	121.539	121.618	121.354	**121.782**	**122.970**
P10-N11-H45	**118.492**	114.087	115.867	113.371	115.045	113.205	**114.932**	**112.085**
C5-Ge1-Cl2	**111.049**	110.715	111.119	110.719	111.328	110.012	**110.634**	**109.937**
C5-Ge1-Cl3	**111.391**	110.573	110.889	110.362	108.807	110.526	**110.481**	**110.093**
O4-Ge1-Cl2	**108.104**	108.325	107.313	108.349	106.537	109.101	**108.178**	**110.862**
O4-Ge1-Cl3	**110.374**	109.550	108.815	110.227	109.412	110.349	**109.570**	**111.139**
Mean Dev.		1.387	1.427	2.247	1.901	1.675	**1.214**	**-**

Imaginary frequencies for the revTPSS and PW6B95 functional were found. Because of PBE0 displaying significantly lower deviation in bond lengths and angles it was determined as the functional.

Compound 4

Calculations on **4a** were performed after the optimization for **2** and **3**. For the sake of comparability the same parameters as in **2** and **3** were chosen and no further benchmarking was performed. No imaginary frequencies were observed.

Table S6. Comparison of experimental data of **4a** and computational data for **2** and **4a** on PBE0/def2-TZVPP/D3BJ/CPCM(THF) level of theory.

Bond lengths [Å]	XRD (4a)	PBE0 (2)	PBE0 (4a)
Ge1-C4	**1.874**	**1.945**	**1.875**
Ge1-O2	**1.795**	**1.792**	**1.800**
Ge1-O3	**1.799**	**1.860**	**1.796**
Ge1-C4	**1.874**	**1.945**	**1.875**
Ge1-N5	**1.860**	**2.059**	**1.858**
N5-P7	**1.673**	**1.627**	**1.675**
C4-P7	**1.735**	**1.705**	**1.728**
C4-P11	**1.707**	**1.690**	**1.703**
Angles [°]			
Ge-1-O2-O3-Ge6	**180.000**	**176.314**	**179.997**
P7-C4-P11	**131.212**	**131.961**	**132.308**
N5-P7-C4	**93.329**	**94.989**	**92.788**
N5-Ge1-C4	**82.363**	**75.647**	**82.604**
Mean Dev.		**1.214**	**-**

The optimized structures are available in xyz format at the end of this chapter.

4.3 Molecular Orbitals

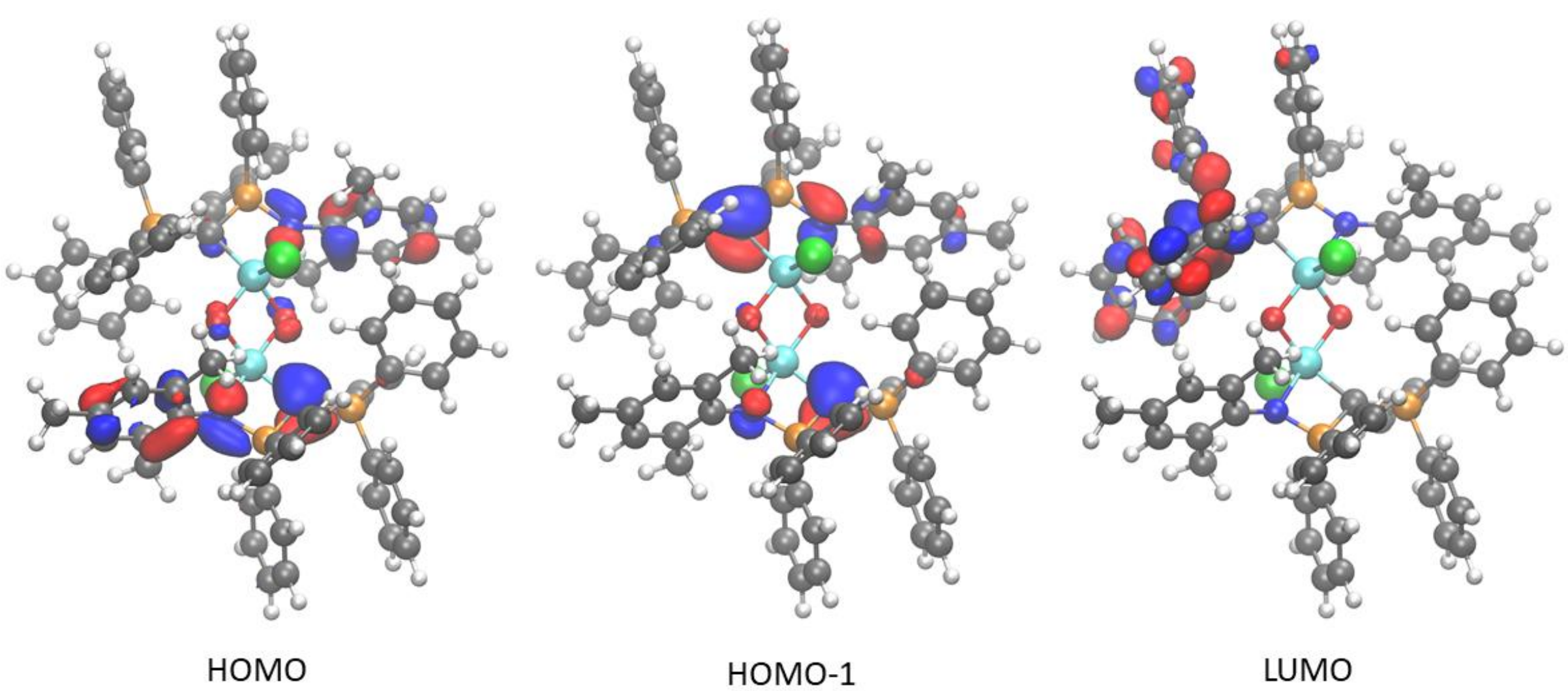

Figure S48. Frontier Orbitals of **2**.

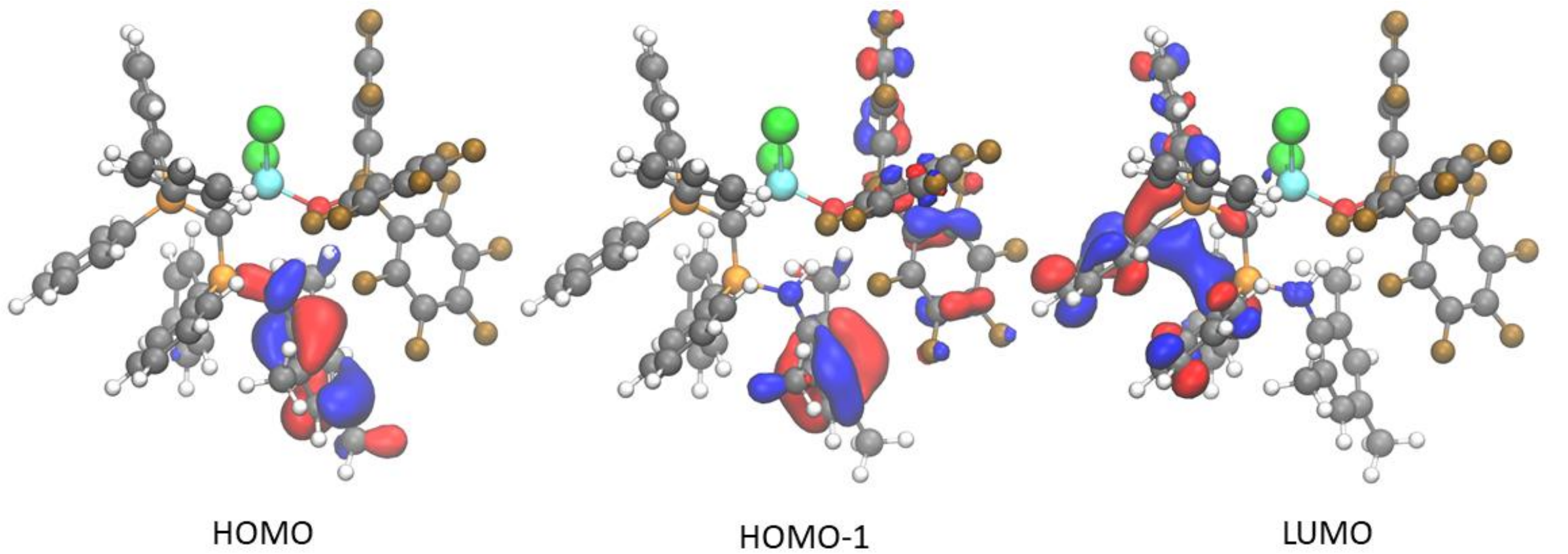

Figure S49. Frontier Orbitals of **3**.

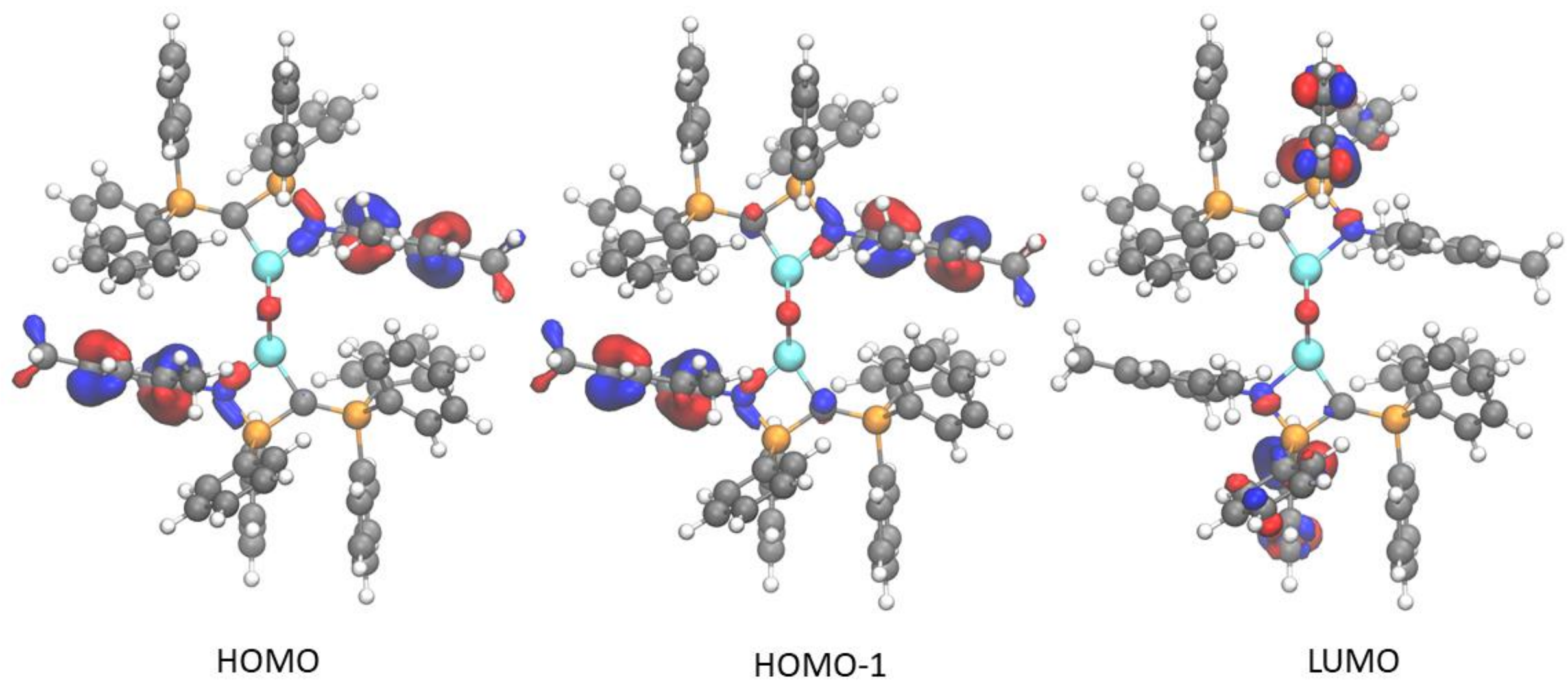

Figure S50. Frontier Orbitals of **4a** (*anti*).

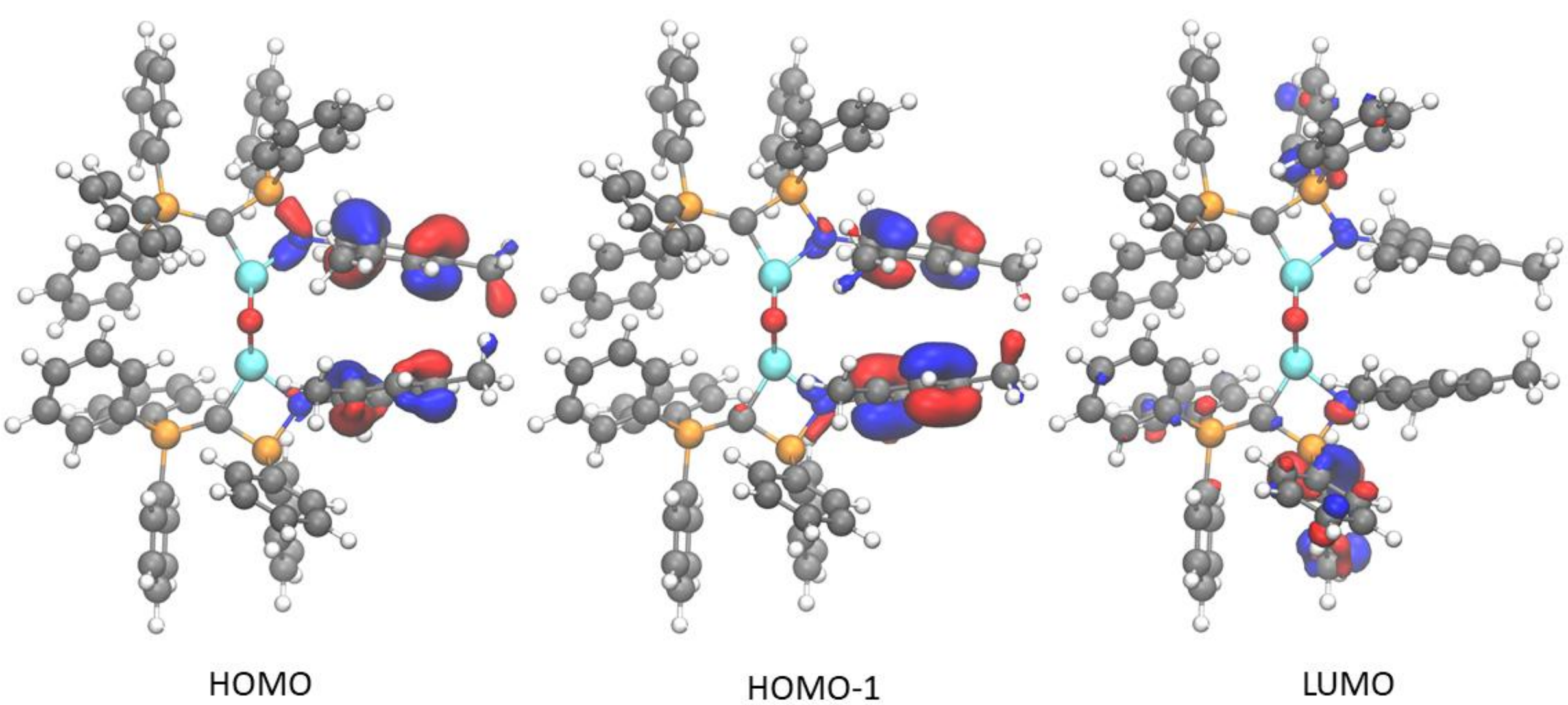

Figure S51. Frontier Orbitals of **4a** (*syn*).

The energetic difference between the *anti*- and the *syn*-conformer is 1.2 kcal/mol.

4.4 NBO analysis

The NBO analysis was performed on the ωB97M-V functional with the def2-TZVPP basis set. Because of technical limitations in the NBO program only the calculations for **3** could be performed on the higher basis set def2-QZVP. Please remark that MO and Atom numbers are 1-based in NBO.

Compound **2**

Table S7. Selected Natural Atomic Orbital (NAO) Partial Charges of **2** (lower atomic index)

Atom	Natural Atomic Orbital (NAO) Partial Charge
Ge	+2.16
Cl	-0.54
C	-1.69
O	-1.31

Bonding Orbitals with contribution of Germanium and bonds of interests as specified in the paper are considered relevant.

Table S8. Selected Natural Bonding Orbitals in **2**.

MO	Occupancy	Atom 1 Contribution [%]	Atom 2 Contribution [%]
165 (BD)	1.92776	Ge2	C5
		23.25	76.75
170 (BD)	1.93037	Ge7	C84
		23.44	76.56

Second order perturbations above 90 kcal/mol are considered relevant.

Table S9. Selected Second Order Perturbations of NBO analysis in **2**.

From unit 1 to unit 2		Energy [kcal/mol]
146. LP (4) Cl1	371. LV (1) Ge2	207.53
Within unit 2		
157. LP (2) N6	373. LV (3) Ge2	91.85
From unit 3 to unit 2		

150. LP (4) O3	372. LV (2) Ge2	144.10
From unit 3 to unit 5		
149. LP (3) O3	376. LV (3) Ge7	97.23
From unit 4 to unit 2		
153. LP (3) O4	373. LV (3) Ge2	96.47
From unit 4 to unit 5		
154. LP (4) O4	375. LV (2) Ge7	149.54
Within unit 5		
163. LP (2) N83	376. LV (3) Ge7	92.29
From unit 6 to unit 5		
161. LP (4) Cl81	374. LV (1) Ge7	209.71

Compound **3**

Table S10. Selected Natural Atomic Orbital (NAO) Partial Charges of **3**.

Atom	Natural Atomic Orbital (NAO) partial charge
C (ylidic)	-1.71
Ge	+1.88
Cl	-0.41
O	-1.10
B	+0.65

Table S11. Selected Natural Bonding Orbitals in **3**.

MO	Occupancy	Atom 1 Contribution [%]	Atom 2 Contribution [%]
167 (BD)	1.96204	Ge1	Cl2
		22.92	77.08
168 (BD)	1.96144	Ge1	Cl3
		23.43	76.57
169 (BD)	1.92922	Ge1	C5
		25.27	74.73
170 (BD)	1.95375	O4	B16
		81.09	18.91

Table S12. Selected Second Order Perturbations of NBO analysis in **3**.

From unit 2 to unit 1		Energy [kcal/mol]
119. LP (3) O4	319. LV (1) Ge1	190.75

Compound **4**

Table S13. Selected Natural Atomic Orbital (NAO) Partial Charges of **4a** (lower atomic index).

Atom	Natural Atomic Orbital (NAO) Partial Charge	
	anti	*syn*
Ge	+2.29	+2.29
N	-1.17	-1.17
C	-1.68	-1.68
O	-1.24	-1.24
P	+1.95	+1.95

Table S14. Selected Natural Bonding Orbitals in **4a**. Format: *anti* / *syn*

MO	Occupancy	Atom 1 Contribution [%]	Atom 2 Contribution [%]
147 (BD) /	1.94374 /	Ge1 26.42	C4 73.58
148 (BD)	1.94217	Ge1 26.27	C4 73.73
150 (BD)	1.96369	N5 72.34	P7 27.66
151 (BD)	1.96212	N5 72.18	P7 27.82

Second order perturbations above 50 kcal/mol representing the bonds of interests are considered relevant.

Table S15. Selected Second Order Perturbations in **4a**. Format: *anti* / *syn*

Within unit 1		Energy [kcal/mol]
143. LP (2) N5	353. LV (1) Ge1	109.95 / 108.29
143. LP (2) N5	353. LV (3) Ge1	87.29 / 98.30
From unit 2 to unit 1		
136. LP (4) O2	353. LV (1) Ge1	63.86 / 61.60
136. LP (4) O2	354. LV (2) Ge1	64.88 / 49.47
From unit 3 to unit 1		
140. LP (4) O3	353. LV (1) Ge1	66.17 / 62.19
140. LP (4) O3	355. LV (3) Ge1	72.17 / 65.32

4.5 IBO analysis

The IBO analysis was performed on the ωB97M-V functional with the def2-TZVPP basis set. Because of technical limitations in the NBO program only the calculations for **3** could be performed on the higher basis set def2-QZVP. Please remark that MO and Atom numbers are 0-based in IBO.

Compound **2**

The Intrinsic Atomic Orbital Partial Charges (IAO) are available in the main text. All bond-like orbitals with contribution of germanium or bonds of interests as specified in the paper are listed here.

Table S16. Selected Intrinsic Bonding Orbitals of **2**.

MO	Atom1	Contribution [%]	Atom2	Contribution [%]
150	1Ge	0.197047	0Cl	0.819100
151	2O	0.891306	1Ge	0.062389
152	2O	0.725550	1Ge	0.246200
153	3O	0.891120	1Ge	0.044076
154	3O	0.779966	1Ge	0.204401
155	4C	0.573748	1Ge	0.444975
156	5N	0.822142	1Ge	0.209884
157	5N	0.854196	1Ge	0.026856
158	6Ge	0.209745	2O	0.777053
159	6Ge	0.247087	3O	0.723194

241	80Cl	0.822035	6Ge	0.192950
242	82N	0.819739	6Ge	0.212083
245	83C	0.553784	6Ge	0.464858

Compound **3**

Table S17. Selected Intrinsic Bond Orbitals of **3**.

MO	Atom1	Contribution [%]	Atom2	Contribution [%]
161	1Cl	0.757048	0Ge	0.220626
162	2Cl	0.747446	0Ge	0.271608
163	3O	0.918893	0Ge	0.045930
164	3O	0.926580	0Ge	0.057118
165	3O	0.708831	0Ge	0.293853
166	4C	0.455870	0Ge	0.606609
178	15B	0.183209	3O	0.845165

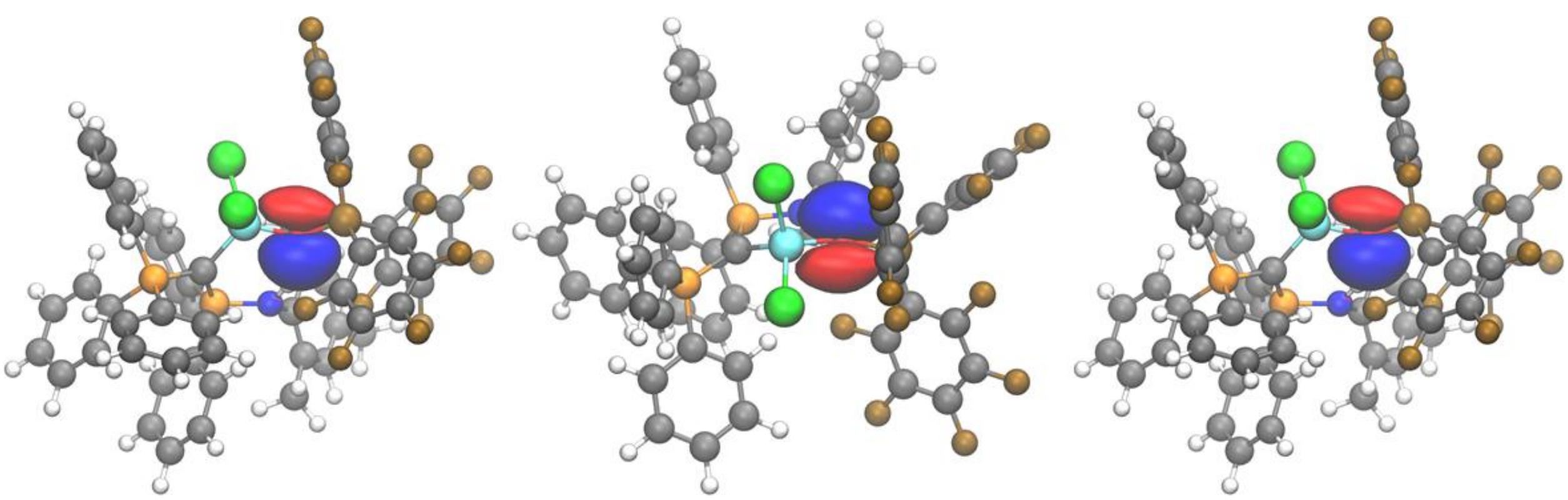

Figure S52. IBO orbitals with contribution of the Germanium and oxygen in **3**. 163 (left), 164 (middle), 165 (right).

Compound **4**

The Intrinsic Atomic Orbital (IAO) partial charges and the Mayer Bond Order indices (MBI) can be considered equivalent for the *syn*-conformer. They are shown in Figure 5 in the manuscript.

Table S18. Selected Intrinsic Bond Orbitals in **4**. The values in the *syn*-conformer can be considered as equivalent.

MO	Atom1	Contribution [%]	Atom2	Contribution [%]
134	1O	0.887554	0Ge	0.052203
135	1O	0.725746	0Ge	0.261319
136	2O	0.887555	0Ge	0.048998
137	2O	0.724036	0Ge	0.256271
138	3C	0.515890	0Ge	0.516738
139	4N	0.633380	0Ge	0.424766
140	4N	0.858733	0Ge	0.053441
144	6P	0.395116	4N	0.602090

4.6 QTAIM analysis

Please remark that the atom indexing of the crystal structure analysis is used for consistency.

Compound **2**

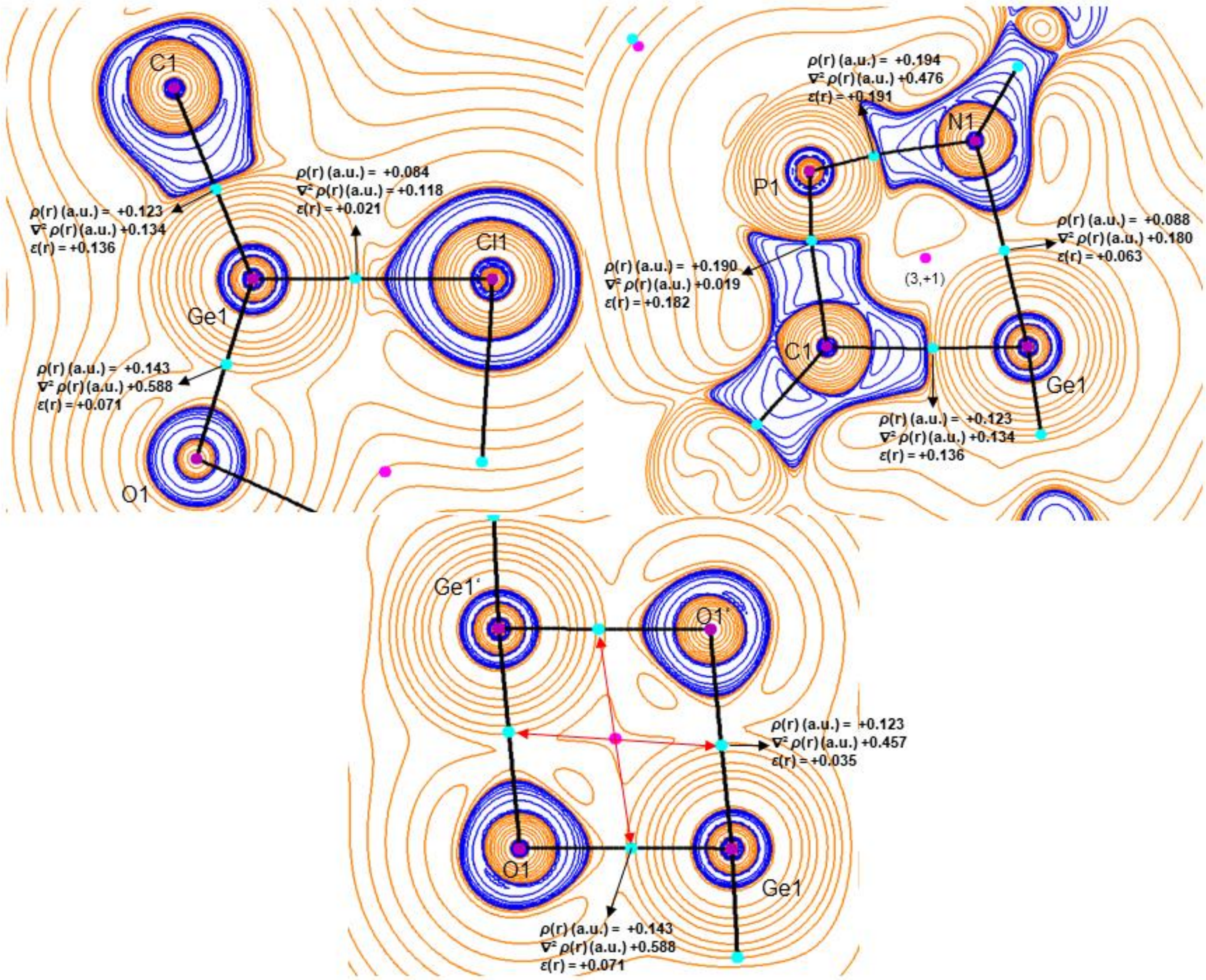

Figure S53. Topology Analysis of **2**. The red arrows indicate that the properties of the BCP are equal.

Compound **3**

The topology analysis of the Ge-O-B linkage can be found in the main paper.

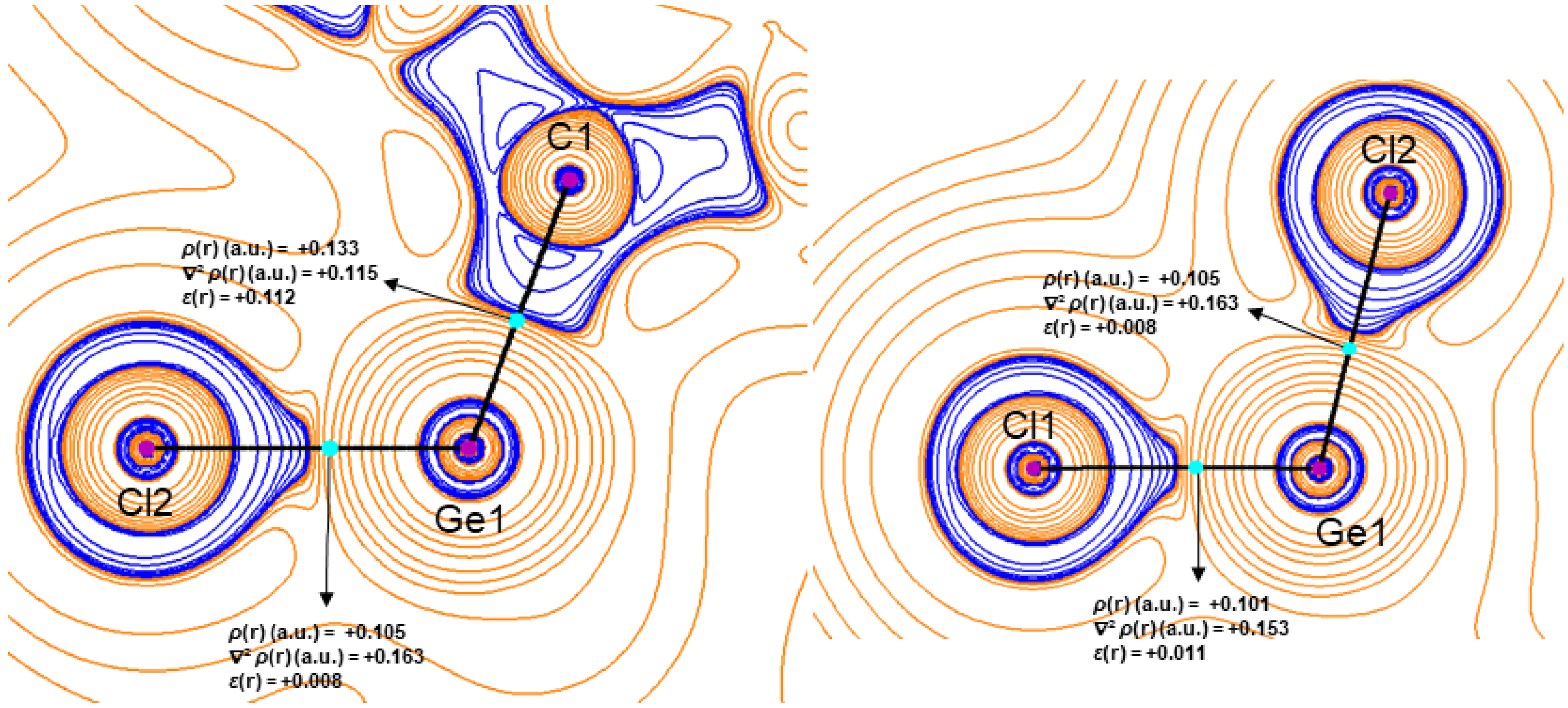

Figure S54. Topology Analysis of **3**.

Compound **4**

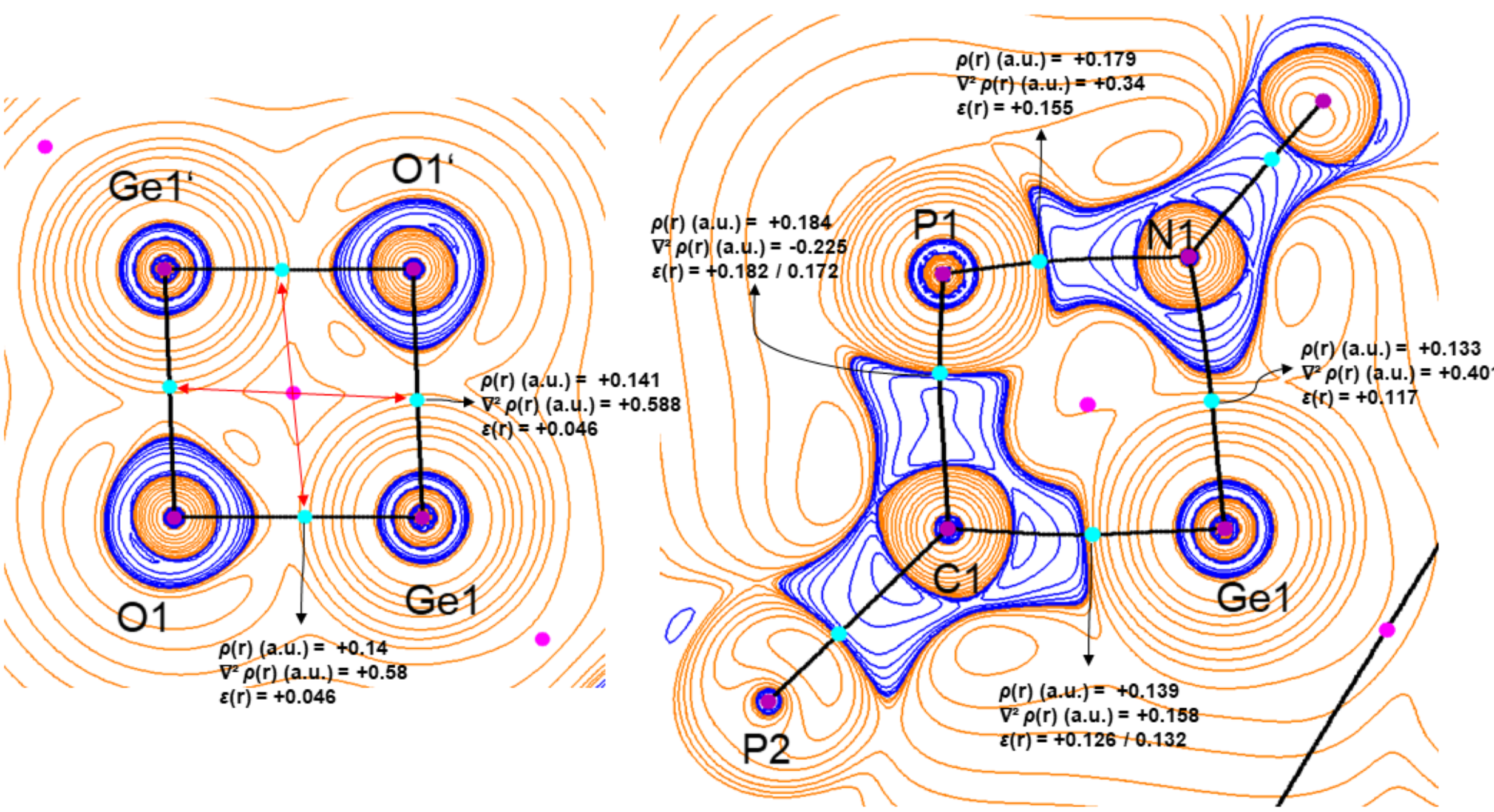

Figure S55. Topology Analysis of **4a**. The red arrows indicate that the properties of the BCP are equal. Values for the *syn*-conformer are given in the format *anti* / *syn* if the difference is larger than 0.02.

NBO and IBO versus QTAIM.

NBO and IBO versus QTAIM

The bonding analysis by NBO/IBO and QTAIM of compound **4** shows partly contradictory results. While IAO and Kohn–Sham molecular orbital analyses suggest the presence of lone pairs at nitrogen and carbon in the ligand backbone localized primarily at the carbon and nitrogen atoms, QTAIM (Figure S55) yielded unusually high ellipticities for all bonds in the Ge-N-P-C cycle, indicative of π-bonding. We attribute this observation to the different conceptual frameworks of the different methods for partitioning and interpreting the electron density. Owing to the diffuse nature of these π-symmetric lone pairs at N and C, the electron density extends toward the Ge and P centers, which may account for the unusually high ellipticities.

4.7 XYZ coordinates

The herein reported structures are the result of the geometry optimization in ORCA.

Compound **2**

```
164
-9788.220349358098
  Cl      0.65535696197615   -1.97105894695426   -2.21812790844192
  Ge     -0.63073885864886   -0.43691755721063   -1.17100381085364
  O       0.41110694023402    0.97781472534369   -0.81913710429445
  O      -0.20794631427109   -0.95008699477979    0.56603919466303
  C      -2.51229067761372   -0.92915336783475   -1.16113631326004
  N      -1.38283526037395    0.38601881871475   -2.90260736108709
  Ge      0.76064024975024    0.50290464969796    0.94504956284301
  P      -2.89070890681021   -0.16588735683466   -2.63811375769606
  C      -4.08552077329962    1.17871495242233   -2.57763694849581
  C      -3.50638364618775   -1.30442828761606   -3.89865234727171
  C      -0.95326954198393    1.44659062873085   -3.72198518105764
  P      -3.36803672972596   -2.03066188833222   -0.20638800956337
  C      -3.85169474529247   -1.41511081601565    1.41951728525524
  C      -2.43582278214729   -3.54630169562694    0.10235718049625
  C      -4.91520570778111   -2.50788410521678   -1.00845488796182
  C      -4.39030209013753    1.89782843381703   -3.73225264595182
  C      -4.60517502691002    1.58815628719554   -1.35468834681511
  C      -5.43676317514530    2.69443661259748   -1.28476413554128
  H      -4.33393618674550    1.05005038094791   -0.45446198705754
  C      -5.73883432203055    3.40724368137565   -2.43693990716677
  H      -5.84018736322765    3.00639171439854   -0.32910241192328
  C      -5.21241624905348    3.01072461241761   -3.65974705325246
  H      -6.38274091838039    4.27689332113685   -2.38263703498075
  H      -5.44070849969864    3.57144926485650   -4.55792057374623
  H      -3.97718498843453    1.59630737786135   -4.68759262783445
  C      -4.79127125132715   -1.25092010430688   -4.42600521795739
  C      -2.65196226886972   -2.33913262690537   -4.28012254244261
  C      -5.21162278978482   -2.21018168476896   -5.33737457678803
  H      -5.47458439775373   -0.47067099467610   -4.11853220861147
  C      -4.35435254012267   -3.22893300695482   -5.72112696622394
  H      -6.21605204048378   -2.16296219054344   -5.74019270194493
  C      -3.07215315960048   -3.29503624942716   -5.18681809188079
  H      -4.68470848768723   -3.97737900098518   -6.43150150131687
  H      -2.40011563208691   -4.09262675531619   -5.47985853338294
  H      -1.65138589665868   -2.38284299084289   -3.86582052570926
  C      -0.36228835485491    1.16518314190926   -4.96697560456838
  C      -1.04346319339276    2.78187874584500   -3.29376927811978
```

C	-0.55897772157633	3.79246994261857	-4.11949250047469
C	-1.63392821624065	3.17833816892563	-1.97712471840595
H	-2.63495370023339	3.59659617487325	-2.10873191979443
H	-1.01493175441414	3.94549237414167	-1.50856594795436
H	-1.69900762071928	2.33932227526148	-1.29233660366579
C	0.02931091938957	3.53324312779521	-5.34697618087388
H	-0.63185564220668	4.81903454944158	-3.77356167557548
C	0.11610540663400	2.20629323147205	-5.75150094707847
C	0.59549574385944	4.63960154584088	-6.18413159513672
H	0.05602549350547	5.57437887616391	-6.02386961009952
H	0.55675856426937	4.39527380564782	-7.24688385219986
H	1.64451290783351	4.82224695998385	-5.93033905496845
H	0.57409460496843	1.96997158705521	-6.70732258510281
C	-1.74694829398239	-4.11454139150727	-0.96598040076528
C	-2.40957131046501	-4.16724705374603	1.34731275351230
C	-1.05434141569443	-5.30160331996353	-0.79693175380684
H	-1.74288678948808	-3.61496321955502	-1.92508137861331
C	-1.04014298641596	-5.92480610571297	0.44323815712431
H	-0.51714448131616	-5.73357281190626	-1.63229502284283
C	-1.71418020444301	-5.35477302718669	1.51357098815602
H	-0.49322182840860	-6.85014997573778	0.57874476449639
H	-1.69040870145012	-5.82836460710143	2.48705628498657
H	-2.91562397379028	-3.72269492931840	2.19367919914095
C	-3.16900867213699	-0.33368004643208	1.96024311156358
C	-4.85698222008944	-2.04216128043371	2.15427739818327
C	-3.47773484763681	0.11426643567047	3.23534676699758
H	-2.38694287951566	0.14664732919402	1.38885367691513
C	-4.47242923161399	-0.51456129541746	3.96950458253632
H	-2.93304731163223	0.95256330141058	3.65167054423646
C	-5.16502977884251	-1.59046382015478	3.42715933488074
H	-4.71249142969717	-0.16586350940403	4.96687345337194
H	-5.94651668888275	-2.07819982134789	3.99692721362273
H	-5.39857450674982	-2.88150071983185	1.73404624217685
C	-5.02467077080334	-3.69641747985596	-1.72244833712843
C	-5.99743963403794	-1.63159342023218	-0.95775693470926
C	-7.17353580981302	-1.93985571990193	-1.62115371119526
H	-5.92761346064526	-0.71108304807279	-0.39282143548354
C	-7.27808699348555	-3.12588480178038	-2.33407842446841
H	-8.00972256453348	-1.25282670705254	-1.57760216092173
C	-6.20428595252349	-4.00236893473282	-2.38253437295177
H	-8.19828767377544	-3.36721066575027	-2.85230632328379
H	-4.19276300873013	-4.38694503366283	-1.76442254043899
H	-6.28130917498652	-4.92833232745749	-2.93882630260428
Cl	-0.57488722471988	2.12469290208557	1.81022643110908
P	2.82910609629778	0.25916738109218	2.67573276256485
N	1.27988008919088	-0.20131952474599	2.81909392155515
C	2.65149879739140	0.89484267724889	1.09956630038183
C	3.95014313449763	-1.13289355504614	2.88544722147958
C	3.36230641510638	1.49742281819047	3.87900351015928
C	0.68158011540092	-1.16011971984371	3.65468194074620
P	3.67886982002107	1.83593288479010	0.14314723524861
C	4.59961625712363	-1.68092109348820	1.78512732138905
C	4.06001236000613	-1.75195059755360	4.12994760138012
C	4.56375840748477	1.43512450731093	4.57473606675789
C	2.53208185303793	2.60668026706576	4.04231059007948
C	0.70443366235757	-2.52631018846145	3.32714358452232
C	-0.02721806721346	-0.74101116536082	4.79583487749295
C	2.83987973210756	3.29277711815798	-0.50835180023578
C	4.35895550079398	0.97197412738215	-1.28800040466041
C	5.11632498789565	2.39283449733659	1.08457653172135
H	4.48341883390697	-1.21995969642259	0.81195896868447
C	5.36592903386899	-2.82710061209335	1.92610173332575

C	4.81687770625378	-2.90419832569989	4.26751432331278
H	3.54503528356242	-1.34166847493536	4.99078097926476
H	5.22969061640797	0.59360414946416	4.43781653789817
C	4.92368877991193	2.46147749300130	5.43778653328489
C	2.89161825272148	3.62823056201570	4.90215883622102
H	1.59752687773531	2.65686446444953	3.49543024258515
C	1.44945499497997	-3.07083133752666	2.14823566392932
C	0.00542723563260	-3.42587424073733	4.12826194001560
C	-0.71521699568372	-1.67415315752966	5.55881605075636
C	2.03507689363390	4.02720455625209	0.35851981743599
C	2.97336692794936	3.69265220397135	-1.83443592188248
C	3.68953394579201	-0.14621522009248	-1.76823504687461
C	5.50797155849130	1.42641428342126	-1.93298497862052
C	6.16641948909477	1.50366644610947	1.30519611861479
C	5.16278065702654	3.66542617621367	1.64407483360193
H	5.87117495333009	-3.24704295372498	1.06485204355395
C	5.47263184012328	-3.44052593236599	3.16659357995915
H	4.89307393220407	-3.38647120989416	5.23439786111299
H	5.86388285059845	2.40692646496569	5.97305059816381
C	4.08895191494214	3.55464233911148	5.60567486491886
H	2.23856685462467	4.48373504540767	5.02606934101161
H	2.39744366279250	-3.51586582723943	2.46185426150879
H	0.86150910075305	-3.85268235162093	1.66405922331357
H	1.65619879208424	-2.30225698580168	1.41081921002032
H	0.02200748764107	-4.47670572257912	3.85697886816094
C	-0.72305543742211	-3.02647717521009	5.23701963397296
H	-1.26954036490027	-1.32975050779148	6.42676312706756
H	1.90301351308015	3.69521269746282	1.37977288448381
C	1.38401523105509	5.16280210089341	-0.09323456197222
C	2.31241003488768	4.82438368133489	-2.28521504442382
H	3.57660798576116	3.11515388334584	-2.52236211315375
H	2.79521102753356	-0.49398585594123	-1.26781212493356
C	4.16133595200105	-0.80800659329131	-2.89139742697991
C	5.98107025732744	0.75883494534697	-3.05081379350169
H	6.03501282545551	2.29725175638962	-1.56154241393806
H	6.14594297819237	0.51433761816481	0.86659418090731
C	7.24916424912778	1.88479455739075	2.08017108835877
H	4.35513768669503	4.36564858886974	1.47606260760946
C	6.24852290960575	4.04289120075962	2.41826998490716
H	6.06487181375853	-4.34108202684965	3.27642740140877
H	4.37198056699698	4.35494970156679	6.27902743229867
C	-1.52533024471734	-4.00843369354815	6.03552427294011
H	0.75491170276816	5.72646418135037	0.58463075338919
C	1.52241918085433	5.56198287612098	-1.41539104222435
H	2.40503022762678	5.12189824064588	-3.32225299557057
H	3.62811927462186	-1.67425621058209	-3.26308321493913
C	5.30746353915575	-0.35799210620635	-3.53055188324667
H	6.87698018305478	1.10966282379537	-3.54827768926055
H	8.06100569619390	1.18755180516062	2.24698760161682
C	7.29130891208371	3.15490179456944	2.63722287271680
H	6.27685656038647	5.03448179643052	2.85273181086245
H	-1.14778743958166	-5.02417773648313	5.90993552839855
H	-1.51245278903178	-3.76371376876166	7.09919890147544
H	-2.57220777261841	-4.00554450634199	5.71612178783560
H	1.00082354572710	6.44167806937381	-1.77281166599363
H	5.67999788060112	-0.87822360956102	-4.40489576530059
H	8.13827176192202	3.45290639484467	3.24332866210976
C	-0.04878454308263	0.69814005096741	5.20197120881540
H	0.92864645327075	1.01741152315009	5.57063594336280
H	-0.29096024863031	1.34169908726562	4.35649440511745
H	-0.77869494536484	0.86063289103091	5.99574647749979
C	-0.24568632712784	-0.24110900920594	-5.46344336266685

H	0.13203825764778	-0.90382419070283	-4.68487958582502
H	-1.21899504660069	-0.63366578397663	-5.76601450651265
H	0.41886776960444	-0.28804130441721	-6.32675585309553

Compound **3**

118
E -7561.907062940575

Ge	3.10431641874275	5.44267152030082	3.12672200491665
Cl	2.42509258514980	6.89364034156295	1.65767108191710
Cl	1.63613145944929	5.55449844833648	4.70333307384599
O	4.64628687826322	5.96406013391295	3.73005291030870
C	3.14256132083745	3.68715591606942	2.37176492735047
P	1.71256432163928	2.71046687040369	2.48156224600144
C	1.66874672764201	1.38970228821418	1.24263200289177
C	0.22798675323543	3.71537752535471	2.22144582370717
C	1.48414781766069	1.88525965325128	4.06815701562506
P	4.57506465441628	3.11982574808161	1.58603205275097
N	5.85325645840907	3.93112499390275	2.23868074958757
C	4.78629473290822	1.35494119235528	1.85302407049339
C	4.64224560196934	3.40310303868235	-0.19229363518093
F	4.78559300794039	4.06700542191078	5.64481378306931
C	5.03499050494434	4.98650025336722	6.58733421666637
B	5.03603781282069	6.97744365703274	4.76204282511582
F	5.09495739601569	3.17104855297905	8.09629950491500
C	5.20657451517645	4.47949896179860	7.86630758093024
F	5.67556227157178	4.86830032980206	10.13835007728717
C	5.50642749192918	5.33458992211231	8.90618564546105
F	5.93683364622115	7.52603495504757	9.63115332920023
C	5.63869136324154	6.68601104714304	8.64218869755110
F	5.61100856976551	8.45994048284045	7.16377501137671
C	5.45732407531949	7.14269282441891	7.35168289785094
F	2.82670386487135	7.85701164417645	6.69660344022289
C	2.97626932729336	8.54275752579320	5.55810339914449
F	1.21203754424239	11.28209026979899	4.00576169570745
C	2.09531722214093	10.31274352107488	4.21166195048449
F	3.12056222292840	10.74874040265305	2.14527944992674
C	3.07002440668265	10.04006487496583	3.27170459491408
F	4.86794139667526	8.80661564949158	2.53967209199289
C	3.97024257309712	9.02015413806715	3.50738488540251
F	1.10774293359970	9.79508723735155	6.27435853344128
C	2.04926920234950	9.55444300070403	5.36322998091148
F	6.38236571250797	9.76735346089735	4.58521602733342
C	7.14122991141068	8.68407568173704	4.38669599780209
F	8.91410613419859	10.20562495858555	4.05372019809254
C	8.47653870554542	8.94995482655174	4.12692339629539
F	10.64556955358279	8.14627487915553	3.69722930155649
C	9.36394035869912	7.90791228656569	3.94896262731546
F	9.74476286955641	5.59274256911461	3.93334784354855
C	8.89910450117557	6.61249484780267	4.05945045658263
F	7.20143428016347	5.11431896409398	4.47182419148864
C	7.55865744271340	6.39645869584336	4.31958725862195
H	5.60637064898053	4.62570724297691	2.94831953322215
C	7.10362615659790	4.14306339471070	1.57995552379537
C	1.50254637651786	1.70250914143259	-0.10557872855229
C	1.78561396803907	0.05531707103597	1.62241910517342
H	1.40657699774277	2.72745469629731	-0.42965634685437
C	1.45457243398508	0.69706974999378	-1.05549634644295
H	1.33023768207385	0.95567419339380	-2.09962374534677
C	1.55560913506690	-0.63172154479171	-0.66833242112505

H	1.51085148024572	-1.41831338046085	-1.41163995002576
C	1.71969785565960	-0.94858001774298	0.67088746670079
H	1.81539988511480	-1.98156885859413	0.98062664990440
H	1.95160447797330	-0.20856843666696	2.65673169671112
C	0.13036294693241	4.51430720921659	1.08439276487912
C	-0.82862693113615	3.69590017658475	3.12915206128502
H	0.95039591551638	4.58295018490321	0.38622298019789
C	-1.01083106339608	5.26085867823544	0.84613278104913
H	-1.06794224751105	5.88000363318802	-0.04027228008814
C	-2.06404564146420	5.22619747398542	1.74741721355718
H	-2.95448055796001	5.81524887603070	1.56480153722189
C	-1.96726365680318	4.44734959379688	2.89120132773605
H	-2.77714987180591	4.42937324361868	3.60973242950992
H	-0.76168078589086	3.11473692509384	4.03730290974212
C	0.43208707465593	0.98723055692825	4.26300987298762
C	2.35259459252097	2.16150361754724	5.11299482072219
H	-0.25480699537970	0.76183889948773	3.45666642447784
C	0.26588082825515	0.37540274243075	5.49263794603227
H	-0.54952776620530	-0.32203722301075	5.63825648552024
C	1.14205381659269	0.65530336362150	6.53494110663676
H	1.00853632262081	0.17359217509887	7.49596550967975
C	2.18393899793573	1.54925020065934	6.34551652390214
H	2.86818929746098	1.77568217927812	7.15367183126799
H	3.16126784571270	2.85897059103748	4.96326994762534
C	4.90704142918232	0.93626527972903	3.17736590226885
C	4.86291186074922	0.42307284215481	0.82496340296926
H	4.86870738633550	1.66242958294183	3.97866512492348
C	5.10145591807848	-0.40072195298458	3.46913213863075
H	5.19016757644791	-0.71934909479119	4.50027598364852
C	5.19719804445309	-1.32683943625896	2.43752431161285
C	5.08003951084079	-0.91382272880664	1.11937914154049
H	5.13995126282050	-1.63578327248784	0.31468098633482
H	5.35922140335466	-2.37366603014667	2.66426037730785
H	4.73301315690595	0.72797065871868	-0.20381165909099
C	3.69526814098658	4.22892588292508	-0.78729661454001
C	5.70497980896522	2.91903617773301	-0.95710343620950
H	2.89929082793889	4.64436279941448	-0.18493886473577
C	3.78524500623699	4.54927206979734	-2.13167295043000
H	3.03994561252628	5.19295197370141	-2.58195257076702
C	4.83506106348038	4.05294226367821	-2.89045302601182
H	4.90981592229021	4.30289422706617	-3.94182383698932
C	5.79596413978661	3.24277305087687	-2.30074385989934
H	6.62377816219567	2.86371479392893	-2.88693336642909
H	6.46878755128164	2.29674166744611	-0.50994575074670
C	8.12992791983513	3.20407568754896	1.70141584631236
C	7.30486724862243	5.32678612559288	0.85572262522631
C	7.99113587068495	1.97064288009476	2.53612258343860
C	9.33361420743127	3.44328847154502	1.04393749770867
H	7.39365687017766	2.16336220782956	3.42622913870902
H	8.97438984625822	1.61245937821502	2.84089293181631
H	7.50033379113543	1.16375920368748	1.98726197995567
H	10.13016378199698	2.71256928371424	1.13968219664670
C	9.55358600544092	4.59378012438360	0.30246671544132
C	10.87078789341290	4.84903286041294	-0.36311264372404
C	8.52692742352004	5.52850028435398	0.23078926990076
H	11.45946645862391	3.93471498169862	-0.44078854842322
H	11.45697273649326	5.57529495473806	0.20698927576152
H	10.73472701890913	5.26067681813654	-1.36484270945099
H	8.67867852374278	6.44572193521318	-0.32893826654414
C	6.24192255386699	6.37179782509818	0.76044940886842
H	5.33762785275365	5.98181354172199	0.29020892093426
H	6.59145513703070	7.21874547951597	0.17115272820428

H	5.95126174209058	6.73583824815590	1.74547058806987
C	5.13013416847560	6.33066065143889	6.27457147491775
C	3.97979441571696	8.23355435872003	4.65182703256675
C	6.61092898156986	7.40346408092435	4.45432867474826

Compound **4a-*anti***

162
E -8867.698125097235

Ge	-1.08518466482874	-0.15584673122134	-0.68857095743049
O	-0.45998850195100	1.05117832717085	0.49195749476853
O	0.45725791904197	-1.05389486877379	-0.48474077445725
C	-2.83131377097173	-0.83245874124118	-0.78388271366434
N	-1.48138213743318	0.44505249497100	-2.40196238191551
Ge	1.08247417856170	0.15315775083880	0.69572999557258
P	-3.04280625264324	-0.15863177829113	-2.36125772442627
C	-4.22030520023887	1.17142547673735	-2.53634478667512
C	-3.40512955890937	-1.37631541766551	-3.62214969384412
C	-0.83575640572209	1.41546635566687	-3.21230581350253
P	-3.76983521184650	-1.91672710773194	0.13476561807733
C	-4.38305123785205	-1.18600762306571	1.65965867032080
C	-2.82245342859803	-3.37141120736341	0.61102989073972
C	-5.17941391373430	-2.45564011617698	-0.83693740896233
C	-4.36835457046944	1.80446438124171	-3.77093817638958
C	-4.88816842222061	1.65647447221591	-1.41662645411678
C	-5.71548488293293	2.76139543924211	-1.53224236765104
H	-4.74547486794173	1.17985674943200	-0.45478253670322
C	-5.86319699879271	3.39057316889042	-2.76053378144885
H	-6.23907348235067	3.13563892770075	-0.66150568243012
C	-5.18806068944968	2.91498249400543	-3.87769260360159
H	-6.50553729044611	4.25824867454275	-2.84869671492266
H	-5.29889657164042	3.41155612505300	-4.83339697184843
H	-3.83757359319188	1.44180367019750	-4.64299823587081
C	-4.60307735955245	-1.37074467459839	-4.32996721353564
C	-2.49782907329421	-2.42068072286770	-3.80238193667679
C	-4.87885830721421	-2.39103374148816	-5.22786153605374
H	-5.32990621749029	-0.58572086307452	-4.17305558884418
C	-3.96703990577688	-3.41701957738694	-5.41686029641869
H	-5.81410477633258	-2.38496511814242	-5.77327916674748
C	-2.77588954211921	-3.43372373662135	-4.70008575404607
H	-4.18599484518185	-4.21284915779827	-6.11828350435590
H	-2.06569424949352	-4.23890650792940	-4.84057077631490
H	-1.57183048354863	-2.43811563563652	-3.24244331397974
C	0.04600312757708	0.97525698210142	-4.21127529546492
C	-0.98021274777980	2.78212245173190	-2.94507917682662
C	-0.26628692524545	3.68777191748862	-3.72547953626726
C	-1.84501077451524	3.31858807375212	-1.84692123416618
H	-2.85475757846593	3.52955782024368	-2.20425204455023
H	-1.42718442036008	4.25279396341425	-1.47265831554847
H	-1.93268833019937	2.62988705710578	-1.00841217173013
C	0.59816734055498	3.28226545435172	-4.72902769231364
H	-0.37529135564935	4.74622539784391	-3.51676299759501
C	0.74491273300394	1.91681912505770	-4.95086483178774
C	1.38464994312512	4.27643785020774	-5.52532618223734
H	1.08717342777978	5.29764436376899	-5.28772655693862
H	1.25000011772786	4.11886074222407	-6.59753462649005
H	2.45375263599986	4.17949388416774	-5.31812521773827
H	1.43401200668269	1.57121731427371	-5.71463080293514
C	-1.76823628643095	-3.79793554926484	-0.19070229264935
C	-3.18733764658576	-4.11499645264973	1.73214276441769
C	-1.08931900738563	-4.96532637396202	0.11965519874562
H	-1.48104748614809	-3.22098842325283	-1.05883312559558

C	-1.46101449597503	-5.70844057882187	1.22977674517427
H	-0.26829468667402	-5.29098631477489	-0.50655995666105
C	-2.50726684670277	-5.28138142969894	2.03619027779496
H	-0.92773431496921	-6.61892702008015	1.47436336522409
H	-2.78931089493882	-5.85365763190262	2.91078897461396
H	-3.99585288521216	-3.78502377370097	2.37140165398052
C	-3.64543839247444	-0.17254680868910	2.26565801972342
C	-5.53509179683443	-1.67224085660485	2.27427309629743
C	-4.05335349181407	0.34507452948062	3.48387013011635
H	-2.75615239072580	0.22074169497688	1.78817877249108
C	-5.20035527150881	-0.14135108688133	4.09594594040390
H	-3.47610586946054	1.13191914514500	3.95274617852191
C	-5.94188232302080	-1.14626007950349	3.49017914933945
H	-5.52075731882305	0.26794739243458	5.04624391021271
H	-6.84037123395164	-1.52169420038888	3.96372573540515
H	-6.11721405051604	-2.45602197468774	1.80502525161297
C	-5.13353724203665	-3.67191207269289	-1.51203199050569
C	-6.28379504626933	-1.61745223332913	-0.97667118854680
C	-7.33595797166389	-1.99734134276209	-1.79289608609973
H	-6.32806872509697	-0.67506847357670	-0.44583881316551
C	-7.28901290838092	-3.20989837373885	-2.46655201365706
H	-8.19418209749384	-1.34587483231808	-1.89984025728250
C	-6.19021244460041	-4.04525720434830	-2.32586256622461
H	-8.11345524298182	-3.50528727457054	-3.10388898770541
H	-4.27708543247335	-4.32510134620253	-1.40451950315289
H	-6.15280353531249	-4.99021059545959	-2.85288904501110
P	3.04025229864957	0.15612705835162	2.36824581038798
N	1.47894872233755	-0.44787981737996	2.40901308487464
C	2.82848933889707	0.83009982696209	0.79097220146862
C	4.21806002543397	-1.17369955880218	2.54304044435826
C	3.40241205597949	1.37368621751429	3.62930078771883
C	0.83357853040701	-1.41863662772353	3.21915541482996
P	3.76656240024239	1.91492894373230	-0.12747727537101
C	4.88594205672264	-1.65841462654806	1.42318801354569
C	4.36638451020288	-1.80688450614811	3.77752367874961
C	4.60053755369633	1.36837073398720	4.33682258761749
C	2.49480613354403	2.41770878547365	3.80997062487981
C	0.97837487436151	-2.78518756748189	2.95163937353645
C	-0.04838887614032	-0.97885275763875	4.21815100973230
C	2.81861176832891	3.36943452390132	-0.60312492164031
C	4.37981441287976	1.18487739023291	-1.65266840645990
C	5.17613653693172	2.45397537892563	0.84415709991162
H	4.74304255068217	-1.18167467664848	0.46143506492604
C	5.71353910483760	-2.76315214168870	1.53855822909372
C	5.18636938298044	-2.91722016807589	3.88403381681556
H	3.83560971652882	-1.44446937129538	4.64969027341104
H	5.32758008072623	0.58360956370919	4.17957849676936
C	4.87621050599560	2.38857896295303	5.23484082998420
C	2.77276152106947	3.43067384780691	4.70779759894563
H	1.56863956444263	2.43492871091163	3.25030784296804
C	1.84324313005594	-3.32123196646816	1.85333060827722
C	0.26466778855188	-3.69118494103453	3.73186176188245
C	-0.74708910323890	-1.92073463824369	4.95751170000904
C	1.76473986005046	3.79558489142930	0.19927308279260
C	3.18263831062617	4.11323510291723	-1.72436346498333
C	3.64256774589375	0.17124349514075	-2.25881930500170
C	5.53154706712191	1.67178860015979	-2.26732249329294
C	6.28071711614553	1.61598605913693	0.98352506863206
C	5.13013554520267	3.67011248834511	1.51948397865036
H	6.23713525577737	-3.13714155250340	0.66771517164030
C	5.86151408933670	-3.39248262456862	2.76673852461317
H	5.29741224255192	-3.41391637275890	4.83964983104233

H	5.81158989132950	2.38270862921276	5.78003396179418
C	3.96409808498157	3.41422547766737	5.42427007983383
H	2.06232537633110	4.23558330327240	4.84863247836587
H	2.85299739615474	-3.53225487021540	2.21061746435265
H	1.42548974927722	-4.25535172241809	1.47875823966030
H	1.93090544200336	-2.63225408314535	1.01504506812818
H	0.37387501454067	-4.74956297873125	3.52287203515782
C	-0.59987689883581	-3.28611606482693	4.73549228275704
H	-1.43640369570774	-1.57545403554095	5.72122930410379
H	1.47819157915302	3.21841977048725	1.06746613076607
C	1.08535621662523	4.96284615451552	-0.11052058255161
C	2.50207144514313	5.27947156361023	-2.02787864105590
H	3.99084731960144	3.78353699517688	-2.36415382011246
H	2.75352379866300	-0.22256128792221	-1.78131463826815
C	4.05054101689528	-0.34587784841968	-3.47722591492373
C	5.93839533823108	1.14631319778887	-3.48342943208800
H	6.11338741334090	2.45570682336914	-1.79795297204365
H	6.32508530264486	0.67370971080079	0.45250835158974
C	7.33294840436668	1.99593599825860	1.79962989023503
H	4.27353027442055	4.32314765085596	1.41226260693272
C	6.18688428977710	4.04352327834723	2.33319041929404
H	6.50408057017110	-4.26001029850848	2.85471327206147
H	4.18297438564902	4.21000244148272	6.12577719750773
C	-1.38554344786889	-4.28066837868757	5.53213141136042
H	0.26461259798143	5.28822348470201	0.51621189169709
C	1.45620682105811	5.70618445609482	-1.22078027186406
H	2.78342386627680	5.85190369732423	-2.90260043246127
H	3.47358630319274	-1.13287302818351	-3.94621463230090
C	5.19723184018396	0.14122555868661	-4.08934588848453
H	6.83664487787600	1.52227722997877	-3.95701294866088
H	8.19131986719313	1.34462106016886	1.90629936573994
C	7.28587940729874	3.20836204765851	2.47352273833930
H	6.14938062190195	4.98837247928128	2.86040168542935
H	-1.09319380160718	-5.30195533523893	5.28860401877321
H	-1.24389649412897	-4.12817294800917	6.60421255473757
H	-2.45545692679072	-4.17878269101991	5.33177371036871
H	0.92255552204146	6.61656587036318	-1.46494029911233
H	5.51767492400239	-0.26767680768915	-5.03980211003650
H	8.11038283549357	3.50380455211145	3.11075468062891
C	-0.26562001465289	0.47744063432975	4.47835652042841
H	0.61466196068273	0.93466583079092	4.93344529801402
H	-0.46820407371965	1.02282288511830	3.55443506940561
H	-1.10726988662004	0.61977779378289	5.15519042924139
C	0.26280455290195	-0.48113354679981	-4.47126320079535
H	0.46529693296185	-1.02641066606952	-3.54726286486005
H	-0.61762395892975	-0.93820633784318	-4.92622201676758
H	1.10437359349252	-0.62381234929023	-5.14812409350592

Compound ***4a-syn***

162
E -8867.697417752981

Ge	-1.09384346751628	0.28460407770786	-1.35855707029924
O	-0.00592780425796	0.37363253025839	0.07606525000745
O	0.14877222741554	1.21678266269195	-2.26307776894948
C	-2.93652588566089	0.56339514439088	-1.56332096384518
N	-1.56173453397086	-1.47004996391974	-1.76792784079638
Ge	1.26491983749886	1.24006782633673	-0.85126130885738
P	-3.18789200218606	-1.10377768891814	-1.93635254482359
C	-3.72256631787075	-1.55499898527171	-3.57936752470432
C	-4.26527308342246	-1.91722737025488	-0.76024253033755
C	-0.81205841090063	-2.60779785948156	-2.16507988558259

P	-3.98373627673291	1.89025664214658	-1.36977664698359
C	-4.03813595078953	2.96192905325654	-2.81466821808237
C	-3.41117256564497	2.91241168945846	-0.00515182098848
C	-5.65220392958620	1.31066822636949	-1.05104211339907
C	-3.72681219138977	-2.90396403506921	-3.93806439362678
C	-4.00353291330751	-0.57833179308128	-4.52973170470653
C	-4.30256395677099	-0.94781270337966	-5.83096712373979
H	-3.96977224295753	0.46885833475157	-4.25902625862041
C	-4.30468521498718	-2.28902332002764	-6.18753510985368
H	-4.52538356758686	-0.18662190519419	-6.56811333873131
C	-4.01421361694284	-3.26531715196738	-5.24335140584318
H	-4.53103546333317	-2.57620215299097	-7.20709709728264
H	-4.01013412149930	-4.31094044550081	-5.52427697189070
H	-3.49669956459232	-3.66953028556302	-3.20668802479081
C	-5.42023327580456	-2.58876698715824	-1.14734747189330
C	-3.96306778835641	-1.77885449553643	0.59511330344390
C	-6.25446131690551	-3.13802176954934	-0.18428204937174
H	-5.68010008844887	-2.67551360986939	-2.19367934851849
C	-5.93889899877417	-3.01728113702980	1.15945287069868
H	-7.15463030660228	-3.65690283407429	-0.48880812957190
C	-4.79244489316046	-2.33409428859950	1.54981793218661
H	-6.59174688830285	-3.44806010676001	1.90879535717535
H	-4.55028775596767	-2.23087316033001	2.60011924045267
H	-3.07580852607621	-1.23858923603805	0.90074898792098
C	-0.62573778079182	-3.66397563610161	-1.26588302242417
C	-0.20513832238791	-2.63105431364495	-3.42815717944809
C	0.54730263450699	-3.74363922648187	-3.78347377025070
C	-0.31959223862617	-1.48301495538369	-4.38089856279616
H	-1.35759556446633	-1.25703471142355	-4.62620663101454
H	0.20827803804790	-1.70856748399658	-5.30663554414723
H	0.11818423416149	-0.57379365446904	-3.96234713154049
C	0.72906540893875	-4.81675538365490	-2.92163266642929
H	1.01515083880431	-3.76291474835541	-4.76185572236207
C	0.13829028345708	-4.75271009717305	-1.66628273321167
C	1.57637722251678	-5.98752246479799	-3.31124393984736
H	1.64389927732207	-6.08574258199115	-4.39496909594631
H	1.18105816330723	-6.91729411913390	-2.89984331039920
H	2.59408025062589	-5.86815212051761	-2.92869133533996
H	0.28177154920807	-5.57122329021798	-0.96837878210129
C	-2.76729691162498	2.32082611503517	1.07976162321520
C	-3.62007627629626	4.28937799748659	-0.02133001969174
C	-2.33377060747705	3.10401585716969	2.13646282937333
H	-2.60575228230260	1.25085570251058	1.10026850269765
C	-2.54581568858277	4.47497648999122	2.11797132250118
H	-1.82486577837400	2.64296228002698	2.97269833276503
C	-3.18953413130426	5.06598418091936	1.04098599806153
H	-2.20058848534749	5.08480618869779	2.94320189409760
H	-3.34509624015868	6.13712446969217	1.01893119334980
H	-4.10304282218381	4.76145261337503	-0.86644241073186
C	-2.93298115101954	2.99466485711008	-3.66063080671172
C	-5.12586159960476	3.79885064674720	-3.05564736800635
C	-2.91495076209219	3.86170825723095	-4.74109919647658
H	-2.09043667939033	2.33819631516589	-3.47929017081036
C	-3.99994116135064	4.69360194189756	-4.98102379345727
H	-2.05469618568214	3.88335184407598	-5.39843167900620
C	-5.10417552591665	4.66121676717377	-4.14004022009232
H	-3.98745625955800	5.36693394259418	-5.82937913690312
H	-5.95243695306200	5.30693841989050	-4.32961785415155
H	-5.98908018707024	3.77707066890426	-2.40162800928451
C	-6.07086840242711	1.11186545098720	0.26156811021372
C	-6.48290890718718	0.95680228792474	-2.11214528371999
C	-7.72778914935417	0.40600821237464	-1.85669085921998

H	-6.16411980161528	1.11090557525272	-3.13525701877922
C	-8.14388934527894	0.20752019572423	-0.54800482632623
H	-8.37228700388910	0.13134185659945	-2.68223590180634
C	-7.31710449938336	0.56112666107835	0.50917686187592
H	-9.11729826678084	-0.22449491840020	-0.35069818421627
H	-5.42725546050954	1.38500493315513	1.08839338682203
H	-7.64158980390929	0.40574544089855	1.53034968567671
P	3.76639650871670	1.81976286391809	-0.66058345854100
N	2.92695127402150	0.46643490838950	-1.18457857323273
C	2.32141247399661	2.65289485875086	-0.21067422691709
C	4.71154039138423	2.51340727982098	-2.00652500874428
C	4.90458276131899	1.52733972845712	0.68889235856923
C	3.32613451012655	-0.59863580159796	-2.03743903556085
P	2.05544483586383	3.99994541545401	0.79808437251985
C	4.25242181268086	3.64996526100577	-2.66336701253538
C	5.84510889971400	1.84449419765778	-2.46863675202192
C	6.15532022793134	2.13714728884928	0.72945483227600
C	4.46637109488695	0.77071592311171	1.77568407602929
C	3.34793325098434	-0.44251872974396	-3.43106957020627
C	3.62610049620569	-1.83965324670464	-1.45938143688737
C	1.30471875896147	3.55469627549899	2.37730249457201
C	0.97503021397728	5.20270489965637	0.00606754586684
C	3.61723824780060	4.79718939220016	1.17618083454202
H	3.35299732172786	4.14329633657166	-2.31754661869307
C	4.93420022720166	4.12903940002192	-3.76978369344869
C	6.51511720458768	2.32078566617791	-3.58248931711831
H	6.19856512548926	0.95079930537111	-1.96854478751755
H	6.49234102746825	2.75331442799869	-0.09287950425896
C	6.96724868455131	1.97399332903282	1.84134761697019
C	5.27858941234370	0.61259631418377	2.88274710911001
H	3.49326867072654	0.29902577690298	1.75253808062246
C	2.95885389227076	0.82223735413703	-4.13277034250765
C	3.73734084052254	-1.52543152968073	-4.21134914861721
C	4.00582071303956	-2.89072906314880	-2.28310112846298
C	1.23054511454050	2.22166246422141	2.76030515075723
C	0.85917277307071	4.54939113196223	3.24684633994466
C	0.11606598782047	4.79203447262446	-1.00931556414450
C	0.96249773875201	6.53108434865332	0.42799520914545
C	4.22183040335471	5.61864871129707	0.22656153593718
C	4.25520025788730	4.53512689683805	2.38504058196907
H	4.58015372588480	5.01706879895645	-4.27819523953730
C	6.06259495361704	3.46404554332631	-4.22924409659698
H	7.39097826432301	1.79915010201962	-3.94718742713646
H	7.93793174559842	2.45265271612048	1.86974198318967
C	6.53209778378967	1.21303258861080	2.91465365980483
H	4.93576975870190	0.02028078355813	3.72181926602165
H	3.84085881502578	1.39173473675466	-4.43278486527058
H	2.40172626668920	0.58202354139339	-5.03917608958616
H	2.33792899688578	1.47277879322698	-3.52276376784286
H	3.75578975542231	-1.40265415358305	-5.28970640942292
C	4.08168578650879	-2.75183765341312	-3.66261311053995
H	4.23595890722286	-3.84988693870137	-1.83134653699773
H	1.57024083233092	1.44643771923027	2.08934952709525
C	0.72028600216222	1.88012758507008	4.00378484905603
C	0.35451267629752	4.20661548557843	4.48868448391102
H	0.90574442042722	5.59261556839174	2.96276040389197
H	0.13156596492240	3.77129676602357	-1.36852153464614
C	-0.75898547798478	5.69648333062115	-1.58752150547734
C	0.08791839551035	7.43304340239054	-0.15627652089590
H	1.64664013342559	6.87074862444595	1.19603125733949
H	3.71996864381878	5.83795604683114	-0.70705183195421
C	5.46559256364199	6.16793371591817	0.48662758416432

H	3.78898936427126	3.89395129301912	3.12195146628132
C	5.49726808664914	5.09271954729872	2.64012254970472
H	6.59176721234409	3.83590492508710	-5.09813916168727
H	7.16763412496406	1.08928352547378	3.78301338796865
C	4.47516494971417	-3.89921711624083	-4.53962788185187
H	0.66430480494238	0.83807972937695	4.29260233992485
C	0.28611326900723	2.87182311613667	4.86952815741062
H	0.01182616637540	4.98299460331953	5.16118966152359
H	-1.42640270975747	5.36701962042688	-2.37403671174070
C	-0.77494883955432	7.01607284143390	-1.16046836963824
H	0.08677275700657	8.46566661375994	0.16956674814111
H	5.93524646113144	6.80671334676277	-0.25075498475065
C	6.10240152420793	5.90497102241522	1.69247001381028
H	5.99467088830753	4.88602497826865	3.57914616545853
H	3.60715527589700	-4.28847671511875	-5.07839325300356
H	5.20759382617566	-3.59055868443781	-5.28780382628481
H	4.89899474173897	-4.71698822433943	-3.95688615889952
H	-0.11026149938689	2.60723858086987	5.84217629518376
H	-1.45677558464403	7.72398496040779	-1.61561361786029
H	7.07432332575038	6.33839491921002	1.89425460287644
C	3.52748499113537	-2.05746133486882	0.01692442032979
H	4.35342515659101	-1.57773024231622	0.54544983248232
H	2.60296273956799	-1.64171590483655	0.42166785258688
H	3.55733626528369	-3.12250247557122	0.24463716393123
C	-1.21139408156097	-3.63594565375497	0.10930346132868
H	-1.06495434065591	-2.66367970379598	0.58218571131317
H	-2.28654357676257	-3.82297068490033	0.08974226947730
H	-0.75048517738617	-4.40098920949558	0.73325397820429

5. References

(1) Bailey, P. J.; Coxall, R. A.; Dick, C. M.; Fabre, S.; Henderson, L. C.; Herber, C.; Liddle, S. T.; Loroño-González, D.; Parkin, A.; Parsons, S. The first structural characterisation of a group 2 metal alkylperoxide complex: comments on the cleavage of dioxygen by magnesium alkyl complexes. *Chem. Eur. J.* **2003**, *9* (19), 4820–4828.

(2) Jörges,M., Mondal, S., Kumar,M., Duari P., Krischer,F., Löffler, J., Gessner, V. H. *Organometallics* **2024** *43* (4), 585-593.

(3) Gazis, Theodore A.; Mohajeri Thaker, Bayan A. J.; Willcox, D.; Ould, Darren M. C.; Wenz, J; M. Rawson, J.; Hill, Michael S.; Wirth, T.; L. Melen, R.; 1,3-Carboboration of iodonium ylides. *Chem. Commun.* **2020**; *56* (22): 3345–3348.

(4) Bergquist, C.; Bridgewater, Brian M.; Harlan, C. J.; Norton, J. R.; Friesner, R. A.; Parkin, G.; Aqua, Alcohol, and Acetonitrile Adducts of Tris(perfluorophenyl)borane: Evaluation of Brønsted Acidity and Ligand Lability with Experimental and Computational Methods. *J. Am. Chem. Soc.* **2000**, *122,* 43, 10581–10590.

(5) Kumar, M.; Remm, H.; Gessner, V. H. A Purely σ-Aromatic, Planar $\{Ge_4\}^{2+}$-Ring. *J. Am. Chem. Soc.* **2026**, *148* (4) 4496–4506.

(6) Sheldrick, G. M. A short history of SHELX. *Acta Cryst A* **2008**, *64* (Pt 1), 112–122.

(7) Sheldrick, G. M. Crystal structure refinement with SHELXL. *Acta Cryst C* **2015**, *71* (Pt 1), 3–8.

(8) Sheldrick, G. M. SHELXT - integrated space-group and crystal-structure determination. *Acta Cryst A* **2015**, *71* (Pt 1), 3–8.

(9) Thorn, A.; Dittrich, B.; Sheldrick, G. M. Enhanced rigid-bond restraints. *Acta Cryst A* **2012**, *68* (4), 448–451.

(10) R. Dennington, T. A. Keith, J. M. Millam **2019**.

(11) a) F. Neese, F. Wennmohs, U. Becker, C. Riplinger *J. Chem. Phys.* **2020**, *152*, 224108. b) F. Neese *Wiley Interdisciplinary Reviews: Computational Molecular Science*, **2025**, 15:e70019.

(12) a) C. Adamo, V. Barone, *J. Chem. Phys.* **1999**, *110,* 6158–6169. b) M. Ernzerhof., G. E. Scuseria *The Journal of Chemical Physics* **1999**, *110*, 5029–5036.

(13) a) F. Weigend, R. Ahlrichs, *Phys. Chem. Chem. Phys.* **2005**, *7*, 3297–3305. b) F. Weigend, Phys. Chem. Chem. Phys. **2006**, *8*, 1057–1065.

(14) S. Grimme, A. Hansen, S. Ehlert, J.-M. Mewes, *J. Chem. Phys.* **2021**, *154*, 064103.

(15) V. Barone, M. Cossi, *J. Phys. Chem. A* **1998**, *11*, 1995–2001.

(16) M. Müller, A. Hansen, S. Grimme, *J. Chem. Phys.* **2023**, *158*, 014103.

(17) A. V. Marenich, C. J. Cramer, D. G. Truhlar, *J. Phys. Chem. B* **2009**, *113*, 6378–6396.

(18) NBO 7.0. E. D. Glendening, J. K. Badenhoop, A. E. Reed, J. E. Carpenter, J. A. Bohmann, C. M. Morales, P. Karafiloglou, C. R. Landis, and F. Weinhold, Theoretical Chemistry Institute, University of Wisconsin, Madison, WI (2018).

(19) G. Knizia, *J. Chem. Theory Comput.* **2013**, *9*, 4834–3843.

(20) R. F. W. Bader, "A quantum theory of molecular structure and its applications" *Chem. Rev.* **1991**, *91*, 893–928.

(21) T. Lu, F. Chen, "Multiwfn: A multifunctional wavefunction analyzer" *J Comput Chem* **2012**, *33*, 580–592.

(22) W. Humphrey, A. Dalke, K. Schulten, *J. Mol. Graphics.* **1996**, *14*, 33–38.

(23) S. Grimme, A. Hansen, S. Ehlert, J.-M. Mewes, *J. Chem. Phys.* **2021**, *154*, 064103.

(24) J. P. Perdew, A. Ruzsinszky, G. I. Csonka, L. A. Constantin, J. Sun, "Workhorse Semilocal Density Functional for Condensed Matter Physics and Quantum Chemistry" *Phys. Rev. Lett.* **2009**, *103*, 026403.

(25) Y. Zhao, J. Phys. Chem. A 2005, 109, 25, 5656–5667

(26) Y. Zhang, W. Yang, "Comment on 'Generalized Gradient Approximation Made Simple'" *Phys. Rev. Lett.* **1998**, *80*, 890–890.
